# Hypoxia-triggered ERRα acetylation enhanced its oncogenic role and promoted progression of renal cell carcinoma by coordinating autophagosome-lysosome fusion

**DOI:** 10.1038/s41419-025-07345-1

**Published:** 2025-01-16

**Authors:** Chun Feng, Demin Kong, Binghua Tong, Yonghui Liang, Fuyi Xu, Yangyang Yang, Yingying Wu, Xiaodong Chi, Pengfei Wei, Yang Yang, Guilong Zhang, Geng Tian, Zhaowei Xu

**Affiliations:** 1 Shandong Technology Innovation Center of Molecular Targeting and Intelligent Diagnosis and Treatment, School of Pharmacy, Binzhou Medical University, Yantai, China; 2 The Second Medical College, Binzhou Medical University, Yantai, China; 3 School of Basic Medicine, Binzhou Medical University, Yantai, China

**Keywords:** Macroautophagy, Transcription factors, Renal cell carcinoma

## Abstract

Estrogen-related receptor α (ERRα) is dysregulated in many types of cancer and exhibits oncogenic activity by promoting tumorigenesis and metastasis of cancer cells. However, its defined role in renal cell carcinoma (RCC) has not been fully elucidated. To reveal the biological function of ERRα and determine the underlying regulatory mechanism in RCC, the quantitative proteomics analysis and mechanism investigation were conducted. The results demonstrated that ERRα promoted the proliferation and tumorigenesis of RCC cells by maintaining lysosome-dependent autophagy flux. ERRα inhibition impaired the transcriptional expression of LAMP2 and VAMP8 and blocked the fusion of autophagosomes with lysosomes, causing the impairment of the autophagy-lysosome pathway and tumor repression in RCC. Moreover, *VHL* mutant-induced hyperactive hypoxia signaling in RCC triggered p300/CBP-mediated acetylation at the DNA-binding domain of ERRα, and this acetylation promoted its affinity toward targeting DNA and Parkin-mediated ubiquitination and proteasome-dependent degradation. This regulatory model enhanced ERRα transactivation on the expression of LAMP2 and VAMP8, which then maintained autophagy flux and RCC progression. Pharmaceutical inhibition on ERRα acetylation-mediated autophagy-lysosome pathway led to growth repression and sunitinib sensitivity of RCC cells. Taken together, this study uncovered a novel regulatory mechanism of acetylation contributing to the transcriptional performance and the oncogenic role of ERRα in RCC progression by modulating the autophagy-lysosome pathway. These findings might provide a novel approach for the clinical diagnosis and resolution of sunitinib resistance of RCC.

## Introduction

Renal cell carcinoma (RCC) is a malignant tumor with high morbidity and mortality rates worldwide. Thus far, no ideal biomarker is available for the early diagnosis of RCC, and patients with early-stage RCC rarely present with apparent symptoms and rapidly develop into the advanced stage involving distant metastases [1]. Hence, identifying reliable markers and developing a systematic scheme for the early diagnosis of RCC are meaningful. RCC often co-exists with genetic *von Hippel-Lindau (VHL)* mutation, which triggers the aberrant activation of hypoxia/hypoxia-inducible factors (HIFs) and downstream signaling pathways. The VHL/HIF regulatory axis is widely recognized as a tumor-promoting contributor to the pathogenesis, distant metastasis, and angiogenesis of RCC [2]. Despite advances in diagnosis and therapy, surgery remains the optimal curative therapy for RCC; however, 30% of patients experienced relapse after the surgery. For advanced-stage RCC, complete removal of the tumor by surgery is highly challenging; therefore, targeted therapy with small-molecule inhibitors is frequently used as the first-line treatment of choice in clinical practice. Sunitinib, a specific inhibitor targeting receptor tyrosine kinase receptors including vascular endothelial growth factor receptor and platelet-derived growth factor receptor, has been approved by the United States Food and Drug Administration as a standard treatment for advanced-stage RCC [3]. Although an effective response is elicited during early application, patients frequently experience loss of sensitivity and develop resistance to sunitinib [4]. Therefore, identifying more promising biomarkers as drug targets or developing effective combined therapies to enhance sunitinib efficacy is an urgent issue for the clinical treatment of RCC.

Macroautophagy/autophagy is a lysosome-dependent dynamic cellular process that maintains the catabolism of redundant proteins or dysfunctional organelles. It exerts an adaptive response and plays a protective role in enhancing the survival of cells under sublethal stimulation such as short-term starvation or a low levels of oxidative damage [5]. As such, accumulating evidence suggests that autophagy acts as a protective guard for tumors in response to clinical treatment including radical therapy, chemotherapy, anti-angiogenesis, and inhibitor-based targeted therapy [6, 7]. Hence, aberrant activation of the autophagy flux in tumor cells is almost correlated with high rates of metastasis and drug resistance [8]. Therefore, it is meaningful to impair the autophagy-mediated survival-promoting effect in tumor cells for increasing the sensitivity of the tumor cell to medical treatment. Although autophagy inhibitors including chloroquine (CQ) and hydroxychloroquine have been used as monotherapy or in combination with chemo- or radiotherapy for cancer treatment including RCC (NCT01550367), the weak targeting ability and low biocompatibility of nonspecific autophagy inhibitors largely limit their clinical application [6]. Investigation on the molecular mechanism of the autophagy flux and identification of reliable drug targets in a specific type of carcinoma will notably improve the strategy of autophagy inhibition in clinical therapies.

Estrogen-related receptor α (ERRα), which belongs to the orphan nuclear receptor superfamily, was first identified by hybridization probed with the DNA-binding domain (DBD) of estrogen receptor α (ERα) [9]. Similar to most nuclear receptors, ERRα acts as a transcription factor by recognizing the conserved estrogen-related response element (ERRE) present on the promoter of target genes. ERRα regulates various physiological events including energy metabolism, bone homeostasis, and immune response [10–12]. Selective inverse agonist of ERRα (such as XCT-790 and compound 29) are widely applied for metabolic or bone disorders in clinical therapy [13]. As for its carcinogenic role, ERRα is dysregulated in many types of cancer including breast, prostate, and colon cancers, and functionally, it exerts oncogenic activity and facilitates tumorigenesis and bone metastasis of cancer cells [14, 15]. However, the defined role of ERRα in RCC remains unexplored and requires elucidation. Furthermore, the functional performance of ERRα is largely dependent on its co-regulators or post-translational modifications (PTMs) in a specific cellular context [16]. Therefore, a comprehensive mechanistic investigation on ERRα in a certain type of cancer will aid its application in clinical diagnosis and targeting therapy.

This study investigated the regulatory role of ERRα acetylation in RCC progression and evaluated its clinical significance in the sensitivity of RCC cells to sunitinib. The label-free quantitative proteomics analysis revealed that ERRα promoted lysosome-dependent autophagy flux by maintaining the fusion of autophagosomes with lysosomes in RCC; this regulation of ERRα on autophagy and tumorigenesis was dependent on hypoxia-induced ERRα acetylation, which was mediated by p300/CREB-binding protein (CBP) and enhanced its transcriptional activity and protective effect of autophagy in RCC. And dual inhibition of ERRα acetylation and autophagy repressed angiogenesis, tumorigenesis, and sunitinib resistance in RCC.

## Materials and methods

### Cell culture and transfection

The cells were obtained from Procell Life Science &Technology Co.,Ltd. (Wuhan, CN). HK2 (Procell #CL-0109) cells were cultured in DMEM/F12 (Gibco #11320033) medium supplemented with 10% fetal bovine serum (FBS) (Gibco #10099) and 1% penicillin/streptomycin (Solarbio #P1400). ACHN (Procell #CL-0021) and A498 (Procell #CL-0254) cells were grown in MEM (Gibco #51985091) medium supplemented with 10% FBS and 1% penicillin/streptomycin.769-P (Procell #CL-0009), 786-O (Procell CL-0010) and OS-RC-2 (Procell #CL-0177) cells were grown in complete RPMI-1640 medium. Caki-1 cells were cultured in McCoy’s 5 A medium (Gibco #16600082) supplemented with 10% FBS and 1% penicillin/streptomycin. HEK293T cells (Procell #CL-005) were cultured in complete DMEM (Gibco #C11965500BT) medium. All cell cultures were carefully maintained in a specific medium at 37 °C with 5% CO_2_. Cell culture in hypoxia condition was carried out with BioSpherix I-Glove Incubator & Oxygen Glovebox at 37 °C with 1% O_2_. Cell transfection assays were conducted according to the manufacturer’s instructions of jetPRIME (Polyplus #114-15) or PEI (Polysciences #23966-1).

### Western blot and immunoprecipitation (IP)

The procedure of Western blot, and IP were described previously [17]. Briefly, the cells were collected and lysed with cold TNE buffer (20 mM Tris-HCl pH 7.4, 100 mM NaCl, 1 mM EDTA, 0.5% NP-40 and 10% glycerol) pre-mixed with protease inhibitor (Biomake #B14001). Protein samples were separated into SDS-PAGE gels and transferred to a PVDF membranes (Millipore #IPVH0010). Membranes were blocked with 5% milk and immunoblotted with the indicated antibodies. The bands were visualized using chemiluminescence. Nuclear and cytoplasmic fractions were isolated according to the procedure in our previous study [18]. For IP, the cell extract was incubated with the relevant antibody overnight at 4 °C, and then 50 μL of Protein A/G magnetic beads (Biomake #B23201) were added into the mixture for 4 h at 4 °C. After being washing thrice with washing buffer, the immunoprecipitated complexes were subjected to SDS-PAGE and immunoblotted with the indicated antibodies. For IP with FLAG or GFP, FLAG-affinity magnetic beads (Biomake #B26101) or GFP-selecctor (Iba-lifescience #2-9131-020) were introduced to perform IP assay. The primary antibodies and reagents are listed in the Table S1 and S2.

### Apoptosis measurement by flow cytometry

The Caki-1 cell were stained with Annexin V-FITC/PI Apoptosis Kit (APExBIO, K2003) and then subjected to flow cytometry to evaluation the apoptosis rates.

### Immunohistochemistry and immunofluorescence staining assays

IHC and IF assays were performed as described in our previous study [17]. The kidney tumor tissue microarray (#HKidE085CS01) was purchased from Shanghai Outdo Biotech Company. Another 19 cancer tissue samples were collected at Yantai Affiliated Hospital of Binzhou Medical University. All individuals who donated tissues for this study provided written informed consent. The samples were incubated with indicated primary antibodies. The IHC staining was performed according to the manufacturer’s instructions (ZSGB-BIO #ZLI-9017). Whole-mount assays for vessels staining with CD31 were carried out as previously described [19]. Briefly, Paraffin sections of matrigel plugs were stained with a primary antibody against CD31 at 4°C overnight. Subsequently, the slides were incubated with Alexa Fluor 647 conjugated with goat-anti-rabbit antibody (Jackson ImmunoResearch #111-605-144) for 1 h at room temperature. The slides were photographed by the laser scanning confocal biological microscope (Zeiss). Antibodies used in IHC and IF are listed in Table S1. IHC scores were determined as previously described [17].

### Identification of acetylation site by LC-MS/MS analysis

Flag-ERRα and HA-P300, as well as Flag-ERRα and MYC-CBP, were co-transfected for 48 h in HEK293T cells, and then lysed using TNE buffer. Flag-ERRα protein was isolated using Flag magnetic beads (Biomake #B26101) and subjected to SDS-PAGE. The appropriate bands containing Flag-ERRα protein were excised and captured after SDS-PAGE and Coomassie blue staining. Samples were rinsed three times with 50 mM NH_4_HCO_3_ in 30% acetonitrile for 20 min. The samples were then incubated for 10 min in 300 μL acetonitrile. After removing the acetonitrile, the gels were reduced by culturing for 30 min at 56 °C with 20 mM dithiothreitol. The gels were then alkylated in the dark for 20 min with 100 mM iodoacetamide. The dried gels were digested overnight with trypsin, and the protein digest was extracted twice with 85 percent acetonitrile and 0.1 percent trifluoroacetic acid. The remaining solution was dissolved in 30 μL of 0.1% formic acid before analysis using an LC-MS system (Thermo Fisher Scientific #EASY-nLC 1200) coupled with an ion trap spectrometer (Thermo Fisher Scientific #LTQ Velos Pro). The obtained MS/MS data were used to search the Swiss-Prot database with Proteome Discoverer (Thermo Fisher Scientific) and MASCOT search engine software (Matrix Science). The acetylated sites of ERRα identified by MS when co-expressed with p300 or CBP are provided in Table S6 and S7, respectively.

### Label-free quantitative proteomics analysis

The detailed procedure of label-free quantified proteomics based on MS has been previously described [20]. Briefly, cells stably expressing shCtrl or shERRα were collected with lysis buffer containing cooktail inhibitor (Applygen #P1265), and then centrifuged at 12,000 rpm at 4 °C for 15 min. The supernatant was collected and the protein concentration of the sample was determined using a BCA protein determination kit (Takara #T9300A). Then 35 µg of protein was added into the digest solution (6 M urea, 100 mM TEAB) to total 100 µL, 10 µL of 100 mM IAA solution was added, and the mixture was incubated for 15 min in the dark at room temperature. Next, 5% (w/w) Tripsin/Lys-C (Wako Chemicals, Osaka, Japan) was added to each sample and incubated overnight at 37 °C. The samples were diluted 1:1 with trifluoroacetic acid (TFA) in acetonitrile (ACN) and MilliQ water (1/5/94, v/v). 20 µg of digested protein were thoroughly dried with a vacuum centrifuge after desalting with C18 Stage-tips with EmporeDisksC18 from Varian (Palo Alto, CA, USA). A QExactive plus Orbitrap mass spectrometer (Thermo Fisher Scientific, Germany) fitted with a nano-electrospray ion source was used for all studies. A measured mass spectrum with a resolution of 70,000 and a continuous high collisional dissociation (HCD) fragmentation spectrum of the ten most common ions were obtained when the mass spectrometer was run in positive ion mode. Peptides were identified using the full MS/dd MS2 (Top10) model, with a scan range of 400–1700 m/z. MaxQuant (version 2.0.0.1) was used to process the raw data using the Xcalibur software (Thermo Xcalibur, RRID:SCR_014593). The UniProtKB database release (April 2021) was used as the forward database. Label-free quantification was carried out with run-to-run matching option.

### Bioinformatics analysis

The relative protein abundance was analyzed based on *p* value and foldchange (shERRα/shCtrl), and protein expression (*p* < 0.05, FC > 1.5 or <0.75) were marked as differentially expressed proteins (DEPs). The genes encoding these DEPs were then subjected to KEGG signaling pathways enrichment. The heatmap and KEGG analysis of proteomics were performed in Hiplot (https://hiplot.org/), a comprehensive and easy-to-use web service for boosting the publication-ready biomedical data visualization [21]. The ERRα binding motif and potential binding sites on the promoters of LAMP2 and VAMP8 were predicted and downloaded from the JASPAR dataset (https://jaspar.elixir.no/) [22].

### Cell Counting Kit-8 (CCK8) and colony formation assays

Cell growth were monitored by CCK8 and colony formation assays, CCK8 was purchased from Biomake (#B34302) and the procedures for CCK8 and colony formation were described previously [17].

### Transwell and scratch wound-healing assays

The protocols for Transwell and scratch wound-healing assays have been mentioned in our previous study. The RCC cells were firstly starved with culture medium containing 2% FBS for 24 h to eliminate the proliferative effect on migration and invasion of cells, which were then re-suspended in minimum culture medium, counted and seeded in apical chambers pre-packaged with or without Matrigel Matrix (Corning #356230) for migration or invasion monitoring, respectively. Basolateral chambers were incubated with complete culture medium. After incubation for 48 h, cells that migrated through the pores were fixed with methanol for 10 min and stained with 0.1% crystal violet. Image were captured using an inverted microscope (Olympus, Japan).

### RNA isolation and qPCR assay

Total RNA was isolated from the cells using RNAiso Plus reagent (Takara #9108). A PrimeScript RT reagent Kit with gDNA Eraser was used to perform a reverse transcription reaction to produce cDNAs (Takara #RR047A). TB Green® Premix Ex TaqTM II (Takara #RR82LR) was conducted for qPCR assays according to the manufacturer’s instructions. The qPCR reaction and raw data were obtained with Thermal Cycler DiceTM Real Time System III (Takara #TP970), and the specific primers for targeting genes are listed in Table S4.

### Chromatin immunoprecipitation (ChIP) assay

ChIP assay was conducted using the SimpleChIP® Plus Enzymatic Chromatin IP Kit (Cell Signaling Technology #900) according to the manufacturer’s instructions. qPCR followed by ChIP was conducted to evaluate ERRα binding enrichment on the promoter regions of *LAMP2* and *VAMP8*. Primers for ChIP are listed in Table S5.

### Luciferase reporter assay

The procedure of luciferase reporter assays and ERRE-luc was mentioned in our previous study [14]. Relative luciferase activity were measured with Bio-Lumi™ II Firefly Luciferase Reporter Gene Assay Kit (Beyotime #RG043S). β-galactosidase was used to normalize the relative activity.

### In vivo mouse xenograft model

All animal experiments were conducted in accordance with guidelines established by the Ethics Committee of Binzhou Medical University’s for Biology and Medical Science, the ethics approval number is #2023-35, the maximum tumor volume did not exceed 2000 mm^3^, and the heaviest tumor weigh was not allowed to over 1/10 of body weight of mice. GemPharmatech Inc. (Nanjing, China) supplied athymic nude mice that were fed at the SPF Animal Center of Binzhou Medical University. Based on body weight, the animals were sorted into the indicated groups, with at least five animals in each group. A human renal cancer xenograft model was created by subcutaneously injection of 5 × 10^6^ RCC cells suspended in 100 μL of PBS (combined with Matrigel at a 1:1 ratio) into the groin. To investigate the effect of various inhibitors on the development and progression of renal carcinoma in vivo, mice were treated with the indicated inhibitors every two days. XCT790 and CBP30 were administered via tail intravenous injection at 10 mg/kg and 15 mg/kg respectively, CQ was intratumorally injected at 10 mg/kg and sunitinib was intragastrically administratered at 60 mg/kg. Every two days after the injection, body weight and tumor volume were assessed every two days after injection. The mice were sacrificed and the tumors were excised, documented with photographs, and weighed under ethical requirements.

### Lentivirus infection and stably cell line construction

siRNAs targeting *ESRRA* were obtained from Tsingke BioTech (Table S3). They were introduced into the cells in accordance with the manufacturer’s instructions. OBiO Technology (shanghai) created lentiviruses expressing Flag-ERRα WT and Flag-ERRα 4KR. shERRα lentiviruses supernatant were provided by Genechem Biotech (Shanghai). Cell culture was infected with concentrated lentivirus supernatant supplemented with 1 g/ml puromycin (MCE #HY-B1743A) to select stable cells as mass pools.

### Autophagy flux measurement by mRFP-GFP-LC3 or colocalization of LC3 and LAMP2

The protocol for monitoring autophagy were conducted according to the 4^th^ guidelines published previously [23]. Cells were transfected with adenovirus containing mRFP-GFP-LC3 vector for 24 h, and then treated with 100 nM chloroquine (CQ) for 24 h. Finally, cells were fixed and photographed using confocal fluorescence microscope. Yellow dots in merged images indicate autophagosomes, and the number of autophagosomes were counted in each cells to reflect the autophagic flux. The relative number of autolysosomes was determined by analyzing coefficient of co-localization between LAMP2 and LC3 in each cell, the relative number of autolysosomes in shCTRL was normalized into 1.

### Lysosomal acidity measurement

Caki-1 with shCTRL or shERRα were planted in a black-walled, clear-bottomed 96-well plate, lysosomal pH were incubated with 5 μM LysoSensor Yellow/Blue DND-160 (Yeasen #HB220308) for 5 min at 37 °C, light emitted at 540/440 nm in response to excitation at 384 and 329 nm were collected by SPARK Cyto300 microplate reader, and the ratio was compared to evaluate the effect of ERRα knockdown on lysosomal acidification.

### Statistical analysis

Statistical analyses were performed by GraphPad Prism 8. Three separate experimental data points were provided as mean ± SD, and two sets of independent samples were compared using the Student’s t-test (unpaired, two-tailed). When comparing various groups one-way ANOVA statistical test was used applying for multiple comparisons. Statistical significance was set at *p* < 0.05. Significant difference were denoted by the notations *p < 0.05, **p < 0.01, ***p < 0.001, and ns indicates *p* > 0.05.

## Results

### Inhibition of ERRα repressed the proliferation and invasion abilities of renal cancer cells

**Fig. 1:**
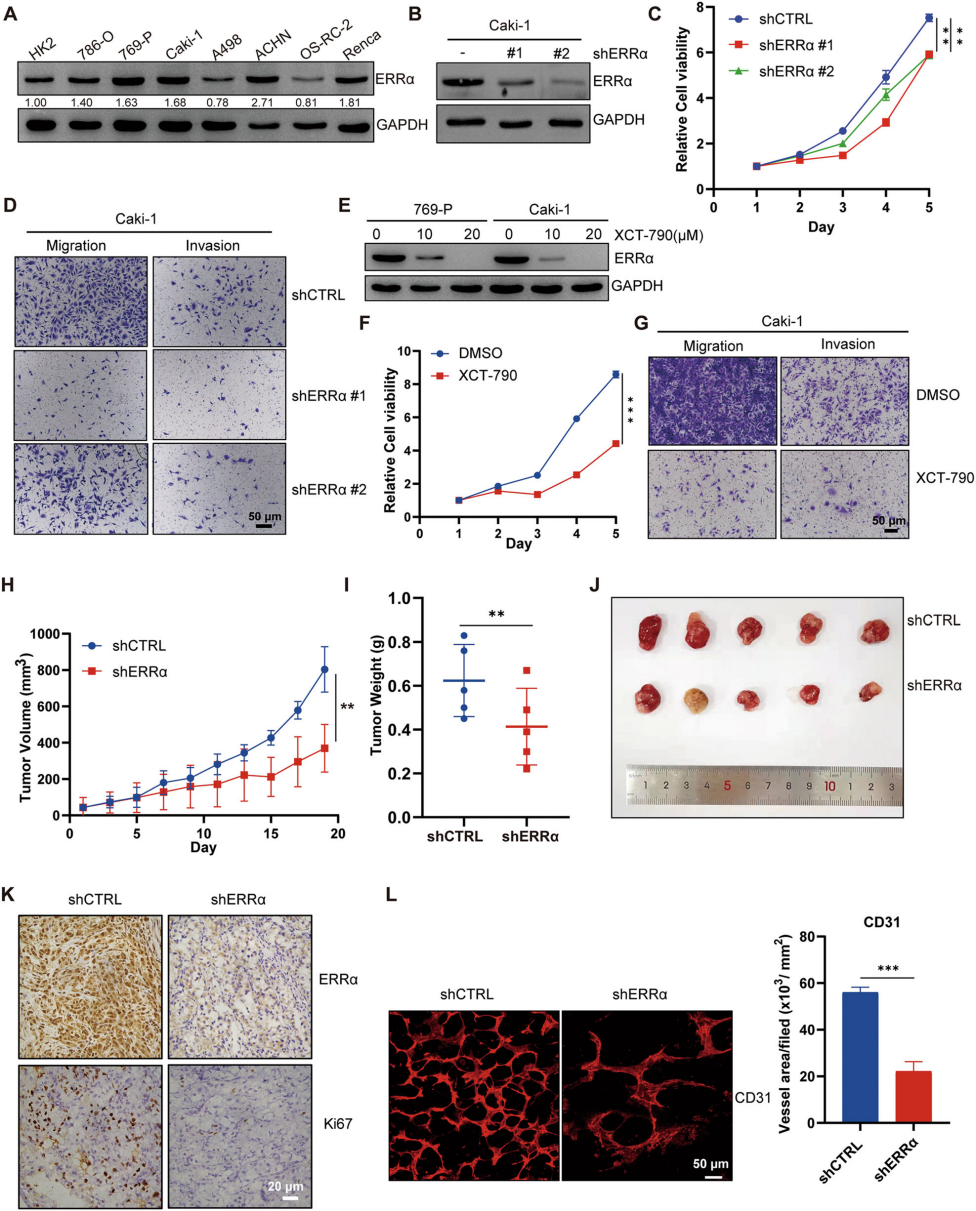
Inhibition of ERRα impaired the proliferation and metastasis of RCC cells in vitro and in vivo. **A** Western blot determined the protein levels of ERRα in the normal kidney cell and RCC cell lines. **B** Western blot confirmed the knockdown efficiency of shERRα in Caki-1. **C** CCK8 assay showed the effect of shERRα on the cell growth of Caki-1, **p < 0.01. **D** Transwell assay determined the migration and invasion of Caki-1 with or without stable knockdown of ERRα. **E** Western blot tested the inhibitory effect of XCT-790 on the ERRα expression in 769-P and Caki-1 cells. **F** CCK8 assay showed the effect of XCT-790 on the cell proliferation of Caki-1 cells, ***p < 0.001. **G** Transwell assay determined the effect of XCT-790 on the migration and invasion of Caki-1. **H** Tumor volume monitoring determined the tumorigenesis of Caki-1 with or without stable knockdown of ERRα by shERRα, *n* = 5, **p < 0.01. **I** Scatter *p*lot showed the tumor weights from shCTRL and shERRα, *n* = 5, **p < 0.01. **J** The images of tumors removed from the indicated two groups of mice. **K** ERRα and Ki67 expression in tumor tissues from two indicated groups were detected by IHC. **L** Whole-mount assay staining with CD31 showed the vascular intensity of tumor tissues from the two indicated groups. Column graph indicated the vessel area statistically analyzed in the tumor tissues from two group, ***p < 0.001.

To determine the endogenous expression of ERRα in normal kidney and RCC cells, protein samples were collected from common cell lines and western blotting assay was performed. Almost all RCC cells possessed a higher abundance of ERRα than the normal renal cortex cell line, HK2 (Figs. 1A). We then performed stable ERRα knockdown with a small interfering RNA lentivirus to explore ERRα function in RCC cells (Figs. 1B). Growth curves and colony formation monitoring showed that ERRα knockdown significantly inhibited the proliferation of RCC cells (Figs. 1C and S1A). The results of scratch wound-healing and Transwell assays indicated that the migration and invasion abilities of the RCC cells were largely impaired owing to ERRα downregulation (Figs. 1D, S1B and S1C). Moreover, XCT-790, a selective antagonist of ERRα, was introduced to confirm the effect of ERRα inhibition on the growth and metastasis of RCC cells. The results of western blotting assay confirmed a significant decrease in ERRα expression after XCT-790 treatment (Figs. 1E). Furthermore, the results of functional investigations demonstrated that ERRα inhibition by XCT-790 significantly repressed the proliferation and invasion of RCC cells in vitro (Figs. 1F, G, and S1D-1G). Additionally, a xenograft mouse model was constructed. The results of in vivo assays revealed that shERRα exhibited a smaller volume and a lesser weight of tumors (Figs. 1H–J and S1H), and histopathologic staining showed that tissues with ERRα knockdown had lower expression of Ki67, the primary marker of proliferation (Figs. 1K and S1I). As vascular endothelial growth factor A (VEGFA), the downstream gene of ERRα, facilitates tumor angiogenesis and plays a role in drug targeting in RCC, whole-mount staining were performed to determine the effect of ERRα knockdown on vessel regeneration ability in RCC. The results showed that shERRα diminished the vessel intensity in the tumor tissues (Figs. 1L). Collectively, these results demonstrated that ERRα exhibited oncogenic activity in facilitating the proliferation and invasion of RCC cells.

### Proteomics analysis revealed that ERRα regulated the fusion of autophagosomes with lysosomes in RCCs

**Fig. 2:**
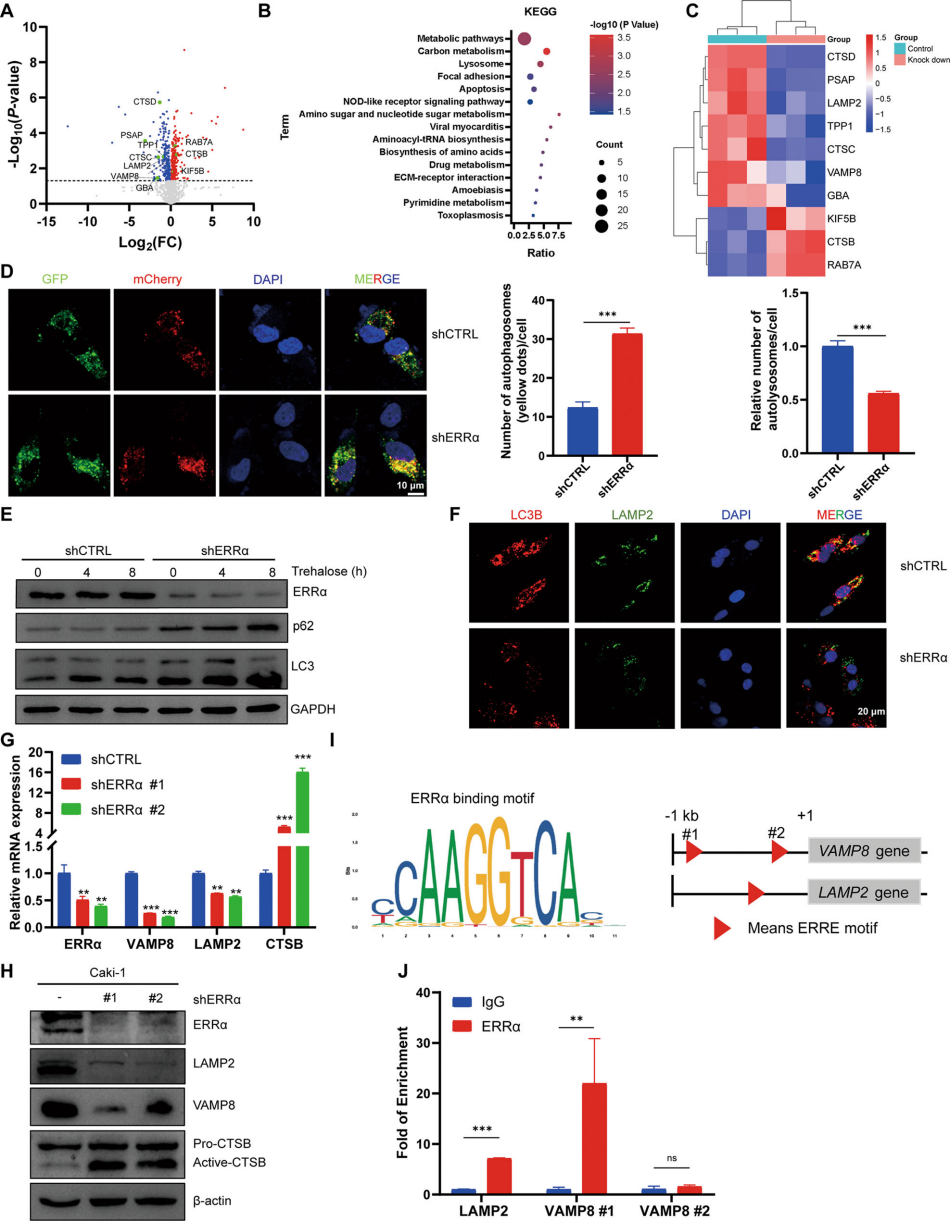
ERRα maintained the autophagy flux and fusion of autophagosome with lysosome in RCC cells through modulating the transcription expression of VAMP8 and LAMP2. **A** Volcano plot showed the 572 proteins presented significant difference between shERRα and shCtrl base on the *p* value, and the lysosome associated proteins were marked up. **B** 209 DEPs (*p* < 0.05, FC > 1.5 or <0.75) were collected to undergo KEGG enrichment analysis to reveal the associated pathways of ERRα in RCC. **C** Heatmap revealed the expression pattern of lysosome related proteins in shCTRL and shERRα groups. **D** Representatives images of IF assay using with mCherry-GFP-LC3B showed the blockage of autophagy flux induced by shERRα. Column graph showed the average number of autophagosomes (Yellow dots counted in merged images) in Caki-1 with siCTRL or siERRα, ***p < 0.001. **E** Western blot verified the changes of LC3 and p62 with or without ERRα knockdown in Caki-1, the autophagy flux was activated by Trehalose. **F** IF staining with LC3B and LAMP2 indicated the decreased fusion of autophagosome with lysosome under the knockdown of ERRα in Caki-1. Co-localization between LC3B (red) and LAMP2 (green) were analyzed to reflect the relative number of autolysosomes in cell, ***p < 0.001. **G** qPCR assay determined the effect of shERRα on the mRNA levels of VAMP8, LAMP2 and CTSB. **H** Western blot determined the protein levels of VAMP8 and LAMP2 with or without ERRα knockdown in Caki-1 cells. **I** The diagram showed the potential ERRα binding sites (ERRE, upper part) within the promoter regions of *VAMP8* and *LAMP2* (bottom part). **J** ChIP assay confirmed the enrichment of ERRα on the promoters of *VAMP8* and *LAMP2* in Caki-1.

To investigate the molecular mechanism of ERRα in RCC, label-free quantitative proteomics analysis was performed based on the total protein samples extracted from Caki-1 cells with shERRα or shCtrl. Among the 1715 identified proteins, 572 showed a significant difference between the shERRα and shCtrl groups (*p* < 0.05). A volcano plot showed that 328 proteins were overexpressed in the shERRα group, whereas 244 proteins were downregulated following ERRα knockdown (Figs. 2A). Proteins that showed differential expression between the two groups and reached a cutoff (Foldchange >1.5 or <0.75) were selected and termed as differentially expressed proteins (DEPs). Overall, 209 DEPs were selected for bioinformatics analysis to explore the potential pathways associated with ERRα in RCC cells. KEGG enrichment analysis revealed that ERRα participated in energy metabolism, apoptosis, focal adhesion, lysosomal pathways, and drug metabolism (Figs. 2B). Previous studies have confirmed the regulatory role of ERRα in metabolism, cell survival, and extracellular matrix (ECM) organization [10, 24, 25]; therefore, the present study focused on the potential role of ERRα in lysosome-related pathways. A heatmap showed that 7 lysosome-related proteins were downregulated and 3 proteins were upregulated with ERRα knockdown (Figs. 2C). As the lysosome regulates the autophagy flux and recycling of biomolecules, circulation of abnormal masses, and aberrant autophagy-lysosome signaling are closely associated with tumor progression and drug resistance [26]. Therefore, this study evaluated the autophagy flux in Caki-1 with shCTRL or shERRα. The results showed that LC3-II/I levels were increased, but SQSTM1/p62 degradation were decreased with ERRα knockdown, and blocking lysosomal function by pre-treatment with CQ and Baf-A_1_ reinforced the accumulation of LC3II/I and SQSTM1/p62 (Figure S2A). mCherry-GFP-LC3B dual fluorescence staining showed that ERRα knockdown displayed defective quenching of green fluorescent protein signals and increased yellow puncta (Figs. 2D). Additionally, autophagy flux induced by trehalose in Caki-1 cells was monitored, the results showed that shERRα increased the accumulation of LC3II/I and SQSTM1/p62 (Figs. 2E), these results suggested that autophagy flux was blocked during the autolysosome formulation. IF assay staining with LC3B (the marker of autophagosomes) and LAMP2 (marker for lysosomes) also confirmed the impairment on the fusion of autophagosomes with lysosomes in the shERRα group (Figs. 2F). Furthermore, changes in the mRNA levels of key regulatory genes involved in the autophagy-lysosome pathway were detected by qPCR. The results indicated that the mRNA expression of LAMP2, VAMP8, CTSC, and CTSB were consistent with proteomics results [26–28], while that of PPT1, RAB7A, KIF5B, PASP, and CTSD was only slightly affected by shERRα (Figs. 2G and S2B). Western blot and IHC were conducted to detect the protein levels of VAMP8 and LAMP2, which are the key regulatory genes involved in autolysosome maturation and fusion between autophagosomes and lysosomes. The results showed a decreased intensity of LAMP2 and VAMP8 in cells and tumor tissues of shERRα (Figs. 2H and S2C). These results indicated that ERRα knockdown impaired the autophagy flux and expression of VAMP8 and LAMP2 in RCC. Furthermore, investigation of the underlying mechanism revealed the presence of a conserved ERRα binding motif (ERRE) on the promoter regions of the two genes (Figs. 2I), and ChIP assays also confirmed the enrichment of ERRα on the promoter regions of *VAMP8* and *LAMP2* (Figs. 2J), suggesting that ERRα served as transcriptional activator and promoted the transcriptional expression of VAMP8 and LAMP2 and maintained the fusion of autophagosomes with lysosomes. Additionally, ERRα knockdown induced a significant increase in CTSB expression at the mRNA and protein levels (Figs. 2G, H). CTSB is a cysteine cathepsin involved in the autophagy-lysosome pathway and lysosome-dependent cell death. Qi et al. reported that lysosomal CTSB cleaved MCOLN1/TRPML1 and reduced TFEB-mediated expression of lysosomal and autophagy-related proteins [27, 29]. This finding was consistent with our result that ERRα knockdown induced an increase in CTSB and a decrease in lysosome-related genes expression, lysosome biogenesis, and impairment of the autophagy-lysosome pathway (Figure S2D). To eliminate CTSB-mediated lysosomal acidification on autophagy flux, LysoSensor probes were introduced, and the results found that shERRα did not induce significant changes in lysosomal acidification (Figure S2E). In conclusion, these results indicated that ERRα regulated the autophagy flux and fusion of autophagosomes with lysosomes by promoting the transcription of VAMP8 and LAMP2 in RCC cells.

### ERRα maintained autophagy-lysosome homeostasis to facilitate the tumorigenesis of RCC

**Fig. 3:**
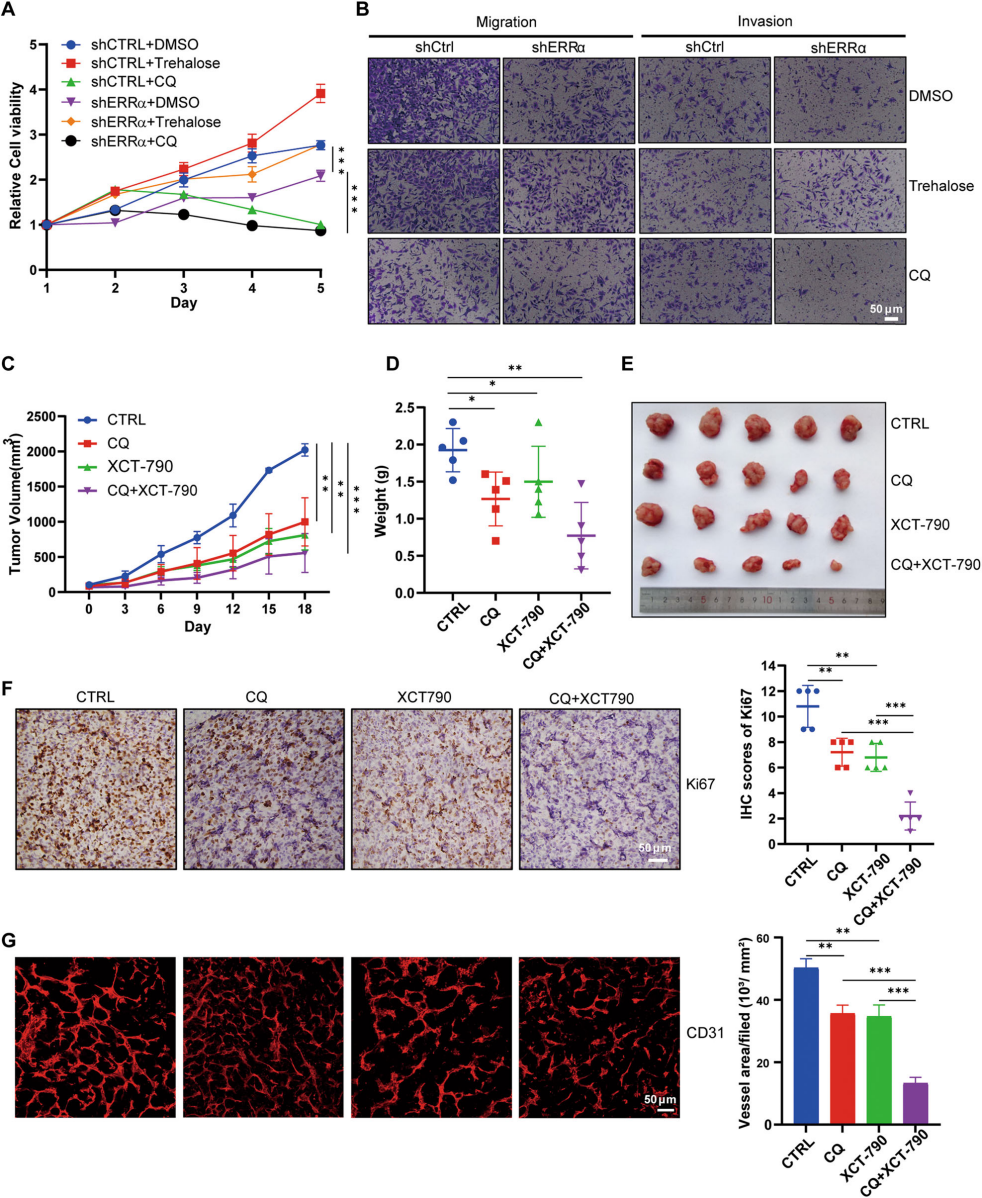
Inhibition on ERRα activity and autophagy flux impaired the progression of RCC cells in vitro and in vivo. **A** CCK8 assay elucidated that the inhibitory effect of shERRα could be rescued by autophagy inducer Trehalose in Caki-1, ***p < 0.001. **B** Transwell assay tested the promotive effect of Trehalose and inhibitory efficiency of CQ on the migration and invasion of Caki-1 with or without shERRα. **C**, **D** Mouse model demonstrated that inhibition on ERRα and autophagy with XCT-790 and CQ repressed the tumor volume (**C**) and weights (**D**) of RCC cells in vivo, *n* = 5, *p < 0.05, **p < 0.01, ***p < 0.001. **E** Tumor images of four groups removed from the mice. **F**, **G** Ki67 and CD31 staining to evaluate the protein expression and vascular intensity of tumor tissues from four indicated groups. Scatter diagram showed the IHC scores of Ki67 in four indicated groups. Bar chart showed the statistical analysis on the relative vessel area of tumor tissues from four groups treated with different inhibitors. **p < 0.01, ***p < 0.001.

To confirm the significance of ERRα-mediated autophagy-lysosome homeostasis in RCC progression, we performed cell growth monitoring and Transwell assays, and the results revealed that trehalose-induced release of autophagy flux, which was blocked by shERRα, partially rescued the suppression of the proliferation and metastasis of RCC cells (Figs. 3A, B). Moreover, we introduced XCT-790 and CQ to effectively inhibit ERRα activity and block lysosome-dependent autophagy. In vivo investigation using a mouse model showed that dual inhibition of ERRα and autophagy by XCT-790 and CQ resulted in the smallest tumor volumes and weights (Figs. 3C–E). Furthermore, IHC staining results revealed that treatment with XCT-790 and CQ significantly repressed the expression of Ki67 (Figs. 3F), accompanied by a decrease in vascular intensity within tumors (Figs. 3G), suggesting that ERRα-mediated autophagy-lysosome pathway might also affect angiogenesis in RCC as reported previously [7, 30, 31]. Moreover, targeting ERRα and autophagy by XCT-790 and CQ did not cause any evident weight loss of mice (Figure S3A), indicating the suitable biocompatibility of this combined therapy. In conclusion, these results indicated that pharmaceutical inhibition of the ERRα-mediated autophagy-lysosome pathway impaired tumorigenesis and angiogenesis in RCC cells.

### Hypoxia induced ERRα transactivation and its ubiquitination-mediated degradation by regulating its acetylation in RCC

**Fig. 4:**
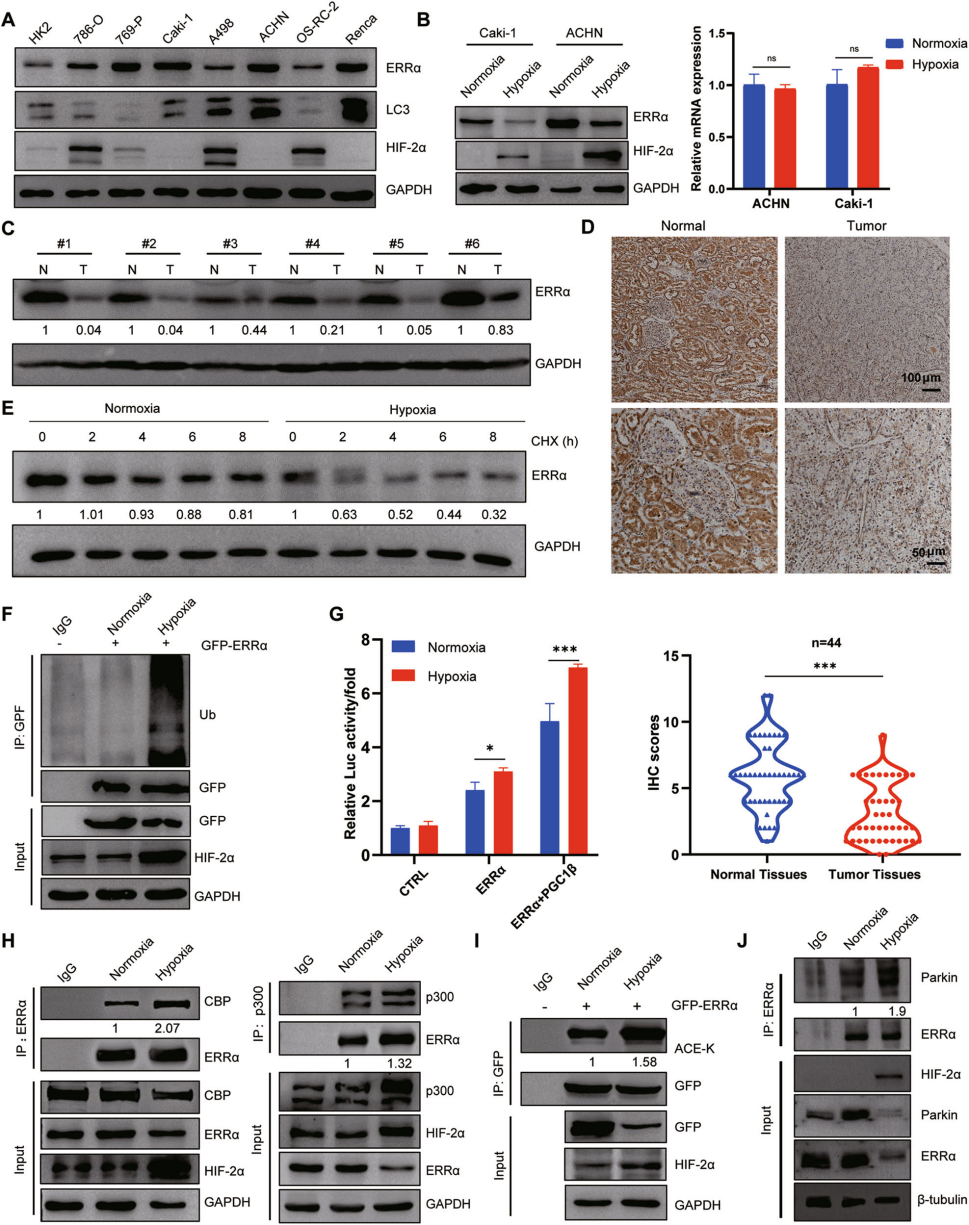
Hypoxia induced transcriptional activity and proteasome-dependent degradation of ERRα via enhancing its acetylation in RCC cells. **A** Western blot detected the protein levels of ERRα and HIF-2α in normal kidney cell and RCC cells. **B** Western blot and qPCR revealed the protein and mRNA levels of ERRα in Caki-1 and ACHN cells under hypoxic or normoxic conditions, ns *p* *>* 0.05. **C** Western blot found a decreased protein abundance of ERRα in RCC tissues compared with normal tissues. **D** Tissue microarry staining showed a significant downregulation of ERRα in renal tumor tissues when compared to normal kidney tissues, ***p < 0.001. **E** Cycloheximide chase assays revealed the half-life of ERRα proteins in Caki-1 under hypoxic or normoxic condition. **F** CoIP assays indicated the discrepant poly-ubiquitination of ERRα under hypoxic or normaxic condition. **G** Reporter luciferase assay showed the transactivation of ERRα on the luciferase activity in hypoxic or normoxic condition. *p < 0.05, ***p < 0.001. **H** IP assays showed the interaction between ERRα with p300 or CBP in Caki-1 under hypoxia or normoxia. **I** IP assay found that hypoxia enhanced the acetylation of ERRα in Caki-1 cells. **J** IP assays revealed that the interaction between ERRα and Parkin was increased in hypoxic condition.

As the *VHL* mutation caused aberrant activation of hypoxia/HIFs signaling, which is frequently observed in the pathogenesis of RCC [1, 2], the effect of hypoxia on the functional performance of ERRα was then determined. Protein expression levels of ERRα and HIF-2α showed that 786-O, A498, and OS-RC-2, which are coupled with the *VHL* mutation [32], had stronger HIF-2α abundance but lower ERRα expression (Figs. 4A). The hypoxic condition was mimicked with 1% O_2_ in *VHL* wild-type RCC cells, ACHN and Caki-1. The data obtained showed that hypoxia induced a decrease in protein expression but not in the mRNA expression of ERRα (Figs. 4B). Moreover, ERRα expression in renal tumor tissues and adjacent normal tissues was evaluated using IHC and western blot, and the results suggested that ERRα had a lower intensity in tumor tissues, as they had a characteristic hypoxic microenvironment (Figs. 4C, D). Based on this phenomenon, these finding led to the speculation that hypoxia might regulate ERRα abundance at the post-translational level by facilitating its ubiquitination and degradation. To confirm this hypothesis, cycloheximide chase and ubiquitination detection assays were conducted, and the results showed that ERRα had a shorter half-life time and stronger ubiquitination under hypoxic condition than under the normoxic condition (Figs. 4E, F). Furthermore, the transcriptional activity of ERRα was evaluated using reporter luciferase assays under both hypoxic and normoxic conditions. The results showed that hypoxia significantly enhanced the transactivation of ERRα when co-expressed with its co-activator PGC1β (Figs. 4G). This interesting phenomenon prompted us to investigate the regulatory mechanism underlying the effect of hypoxia on the decrease in stability but enhancement of the transcriptional activity of ERRα. As the initiation of the transcriptional complex containing ERRα and PGC1β is dependent on the recruitment of p300/CBP, the acetyltransferases activity of p300/CBP is activated by hypoxia [33, 34], and ERRα acetylation mediated by acetyltransferases largely affects its transcriptional activity [35, 36]. Therefore, we proposed that hypoxia might activate the acetyltransferases activity of p300/CBP and subsequently, promote the recruitment of p300/CBP to ERRα and enhance ERRα acetylation, this effect consequently increased the transcriptional activity of ERRα. To test this hypothesis, the interaction between ERRα and p300/CBP and the acetylation levels of ERRα under hypoxic and normoxic conditions were determined. The results revealed that hypoxia promoted the interaction between ERRα and p300/CBP and caused a higher degree of acetylation of ERRα than normoxia (Figs. 4H, I). Another noteworthy issue is that hypoxia promoted the ubiquitination-dependent degradation coupled with increased transcriptional activity of ERRα in RCC, this unconventional model provoked us to identify the ubiquitin E3 ligase that could response to hypoxia signaling and facilitate transcriptional performance of ERRα. Shires et al. reported that Parkin transferred into nucleus under hypoxia and then increased ubiquitination-dependent degradation and activated the transcriptional activity of ERRα in HeLa cell [37]. Based on this, IP assays were conducted and the results showed that hypoxic treatment increased the interaction between Parkin and ERRα in Caki-1 cells (Figs. 4J), and Parkin knockdown significantly increased basal ERRα and decreased ERRα ubiquitination (Figure S3B), which might account for the stronger ubiquitination and transcriptional activity of ERRα under hypoxia. In conclusion, these data indicated that hypoxia prevailing in RCC triggered a higher transcriptional activity of ERRα by modulating p300/CBP-mediated acetylation and Parkin-mediated ubiquitination.

### ERRα was acetylated by p300/CBP at K100, K125, K138, and K146 in RCC

**Fig. 5:**
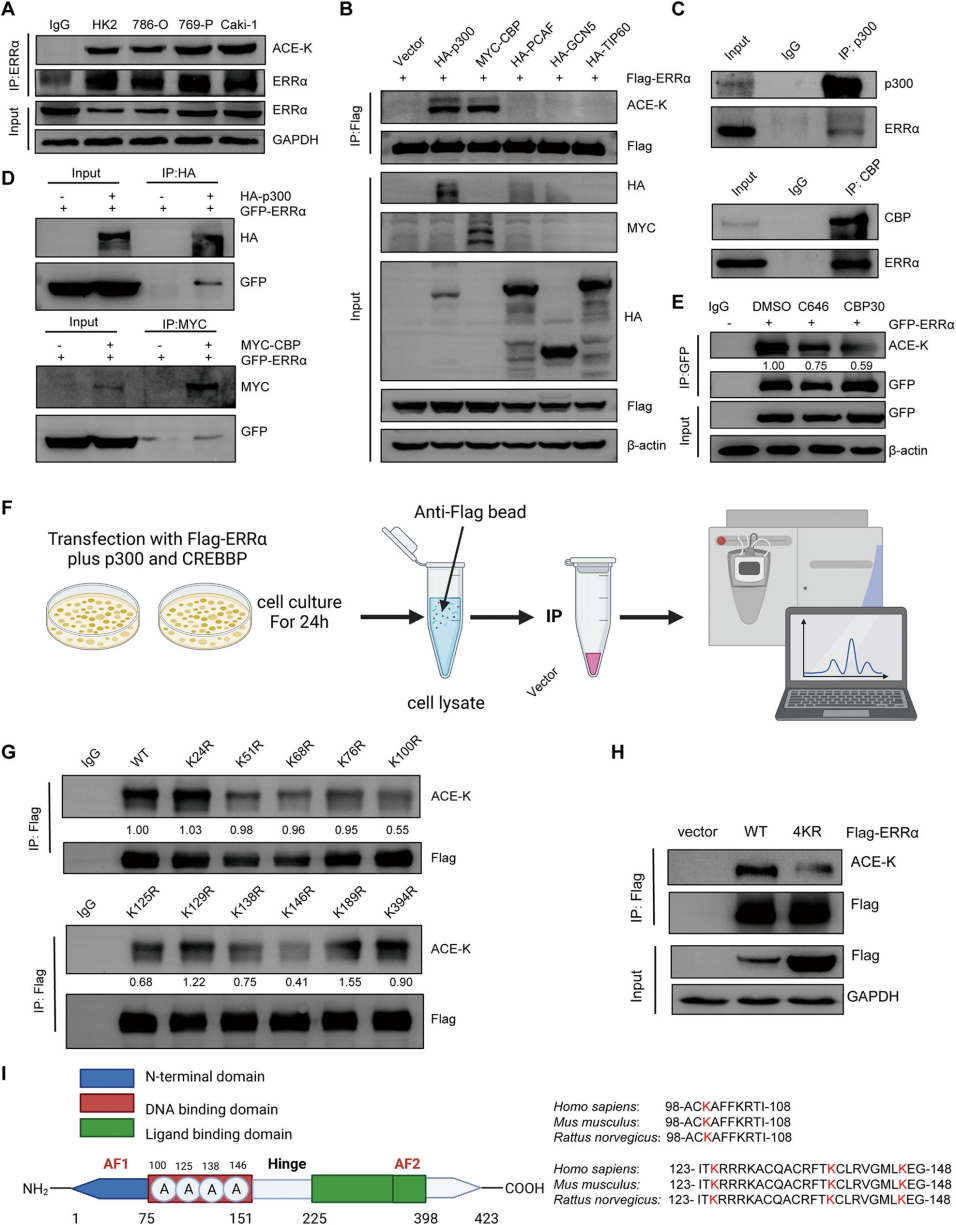
ERRα was acetylated by p300/CBP at K100, K125, K138, K146 residues. **A** CoIP assays showed the acetylation of endogenous ERRα in renal normal cell and RCC cells in presence with TSA and NAM. **B** IP assay revealed the acetylation of ERRα when ERRα coexpressed with different acetyltransferases. **C**, **D** CoIP assay confirmed the endogenous (**C**) and exogenous (**D**) interaction between ERR and p300/CBP. The endogenous IP was conducted in Caki-1 and exogenous IP was conducted in HEK-293T cells. **E** IP assay evaluated the effect of p300/CBP inhibitors on the acetylation of ERRα. **F** The diagram illustrated the procedure of identification for acetylated sites of ERRα by IP and MS. **G** IP assay showed the main acetylated sites of ERRα were located at K100, K125, K138 and K146. **I** The diagrams stimulated the domain location (left section) and evolutionary conservation (right section) of four acetylated sites of ERRα. **H** The result of IP found the acetylation levels of WT or 4KR ERRα in presence with TSA and NAM.

A previous study demonstrated that ERRα acetylation mediated by p300/CBP-associated factor (PCAF) at Lys-129, Lys-138, Lys-160, and Lys-162 crippled its transcriptional activity [35], but the present study data indicated that hypoxia induced a higher degree of acetylation coupled with stronger transactivation of ERRα in RCC cells. Therefore, these finding led to the speculation that a novel mechanism of acetylation on ERRα activity might exist in RCC cells. To support this possibility, endogenous acetylation levels were confirmed by immuoprecipitation (IP) in different RCC cells (Figs. 5A). Next, the acetyltransferase responsible for ERRα acetylation was screened using IP assays. The results showed that positive acetylation bands could be detected only when ERRα was co-expressed with p300 and CBP (Figs. 5B). Furthermore, the interaction between ERRα with p300/CBP in RCC cells was confirmed by endogenous and exogenous IP assays (Figs. 5C, D). Selective inhibitors of p300 (C646) and CBP (CBP30) largely repressed the acetylation levels of ERRα (Figs. 5E). Overall, these data indicated that the acetyltransferase p300/CBP mainly catalyzed ERRα acetylation in RCC cells. Next, acetylated ERRα was collected, purified, and assessed through IP and SDS-PAGE assays, after which they were subjected to mass spectrometry analysis to identify the acetylated residues within ERRα (Figs. 5F and S3C). The results showed that 11 lysine residues within ERRα were acetylated when co-expressed with p300 or CBP (Figure S3D). To explore the important acetylation residues of ERRα, single lysine KR mutants (lysine was mutated with arginine to mimic acetylation deficiency) were constructed, and the results showed that K100R, K125R, K138R, and K146R significantly decreased the acetylation levels of ERRα (Figs. 5G and S3E). Structural and conservation analyses indicated that these four residues were located in the DBD of ERRα and had a high evolutionary conservation between species (Figs. 5I). Moreover, the 4KR mutant was constructed and subjected to acetylation examination, and the results indicated that mutants of the four lysine residues largely impaired ERRα acetylation (Figs. 5H). Collectively, these data demonstrated that ERRα could be acetylated in RCC cells by p300/CBP at K100, K125, K138, and K146.

### Acetylation increased the ubiquitination and degradation of ERRα but enhanced its transcriptional activity

**Fig. 6:**
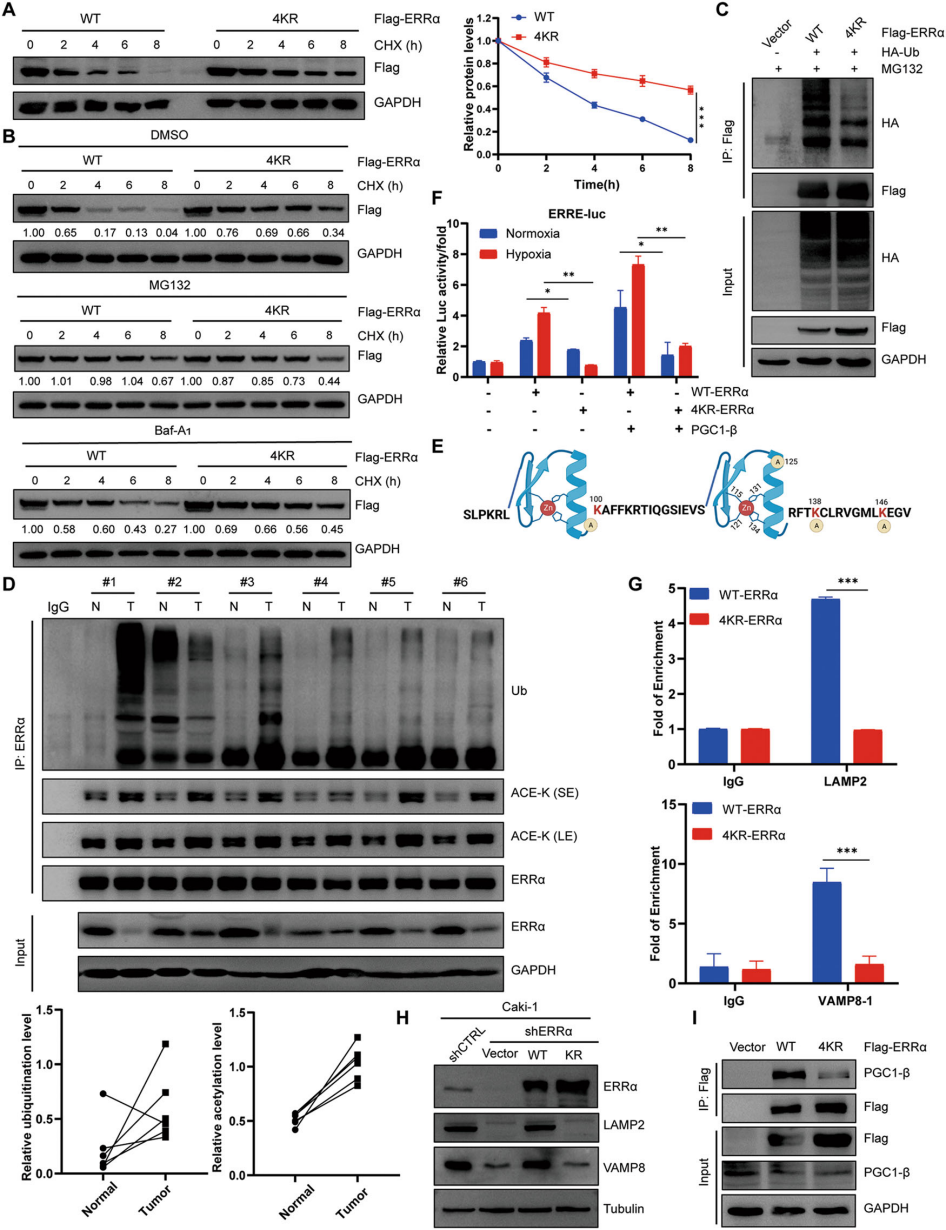
Acetylation of ERRα facilitated its transactivation and proteasome-dependent degradation. **A** Western blot determined the half-life times of WT or 4KR ERRα under treatment with CHX. Line chart indicated the relative protein intensity of WT or 4KR normalized with GAPDH. **B** Cycloheximide chase assays revealed the effect of MG-132 and Baf-A_1_ on the stability of WT or 4KR proteins. **C** IP assay showed the different ubiquitination modification of WT and 4KR under the treatment with MG132. **D** Six kidney tumor tissues and their paired conjugated normal tissues were analyzed to show a reverse tendency between acetylation and ubiquitination of ERRα. **E** The schematic diagram illustrated the major acetylated sites of ERRα located in its DBD domain that contains two Zinc-finger domains. Green cycle labeled-A indicated the lysine modified with acetyl. **F** Reporter luciferase assay tested the transcription activity of WT or 4KR ERRα under hypoxic or normoxic conditions. *p < 0.05, **p < 0.01. **G** ChIP evaluated the enrichment of WT, 4KR and 4KQ on the promoters of LAMP2 and VAMP8, ***p < 0.001. **H** Western blot revealed the effect of rescued expression of ERRα WT and 4KR on the protein levels of LAMP2 and VAMP8 in Caki-1 with or without ERRα knockdown. **I** The interaction between endogenous PGC1β and exogenous WT or 4KR ERRα were determined by IP.

Considering the regulatory effect of hypoxia on the acetylation and ubiquitination of ERRα in RCC, we tested whether there is a crosstalk between acetylation, ubiquitination and transcriptional performance. Firstly, the cytoplasmic and nuclear proteins under nomoxia or hypoxia were isolated and then IP assays were conducted, the results found that hypoxic treatment increased transition of Parkin and ERRα into nucleus, coupled with stronger acetylation and ubiquitination modifications of ERRα than normoxia (Figure S4A), which indicated that hypoxia-induced ERRα acetylation increased its nuclear location and might boost its transcriptional activity, which reversely enhanced its ubiquitination and degradation. Moreover, we found that 4KR-ERRα had a higher protein expression level than wild-type (WT) ERRα (Figs. 5H). These results suggested that ERRα acetylation was essential for protein stability and transcriptional performance. Hence, the half-life of WT ERRα and KR mutant was evaluated and the results demonstrated that the acetylation deficient KR mutant had a longer half-life time and higher stability than WT (Figs. 6A). The degradation-dependent pathway was determined using proteasome and lysosome inhibitors, and the results revealed that the proteasome inhibitor MG132 could rescue the degradation of WT ERRα, whereas lysosome inhibition by Baf-A_1_ had only a minimal effect (Figs. 6B). Furthermore, the IP assay results revealed that WT ERRα had a stronger ubiquitination modification than the KR mutant (Figs. 6C); indicating that acetylation mainly increased ERRα degradation in an ubiquitination and proteasome-dependent manner. The crosstalk between acetylation and ubiquitination of ERRα was also confirmed in histopathological renal tumors, and the results showed stronger acetylation and ubiquitination levels in tumor tissues than in normal tissues (Figs. 6D). These data indicated that ERRα acetylation promoted its ubiquitination and proteasome-dependent degradation in RCC. The aforementioned data revealed that the four acetylated sites of ERRα were mainly located in the DBD domain, and structural analysis indicated that K100 and K125 were located within two zinc-finger domains (P-Box and D-box), which are responsible for binding to the major groove of the DNA helix, whereas K138 and K146 were located within the C-terminal extension of the DBD, and this sequence aimed to stabilize the interaction between the DBD and the DNA (Figs. 6E). Therefore, the acetylation of ERRα might affect its DNA affinity and transcriptional performance. Reporter luciferase monitoring assay revealed that WT ERRα had higher activity than the KR mutant under hypoxic and normoxic conditions (Figs. 6F). ChIP assays confirmed that acetylation maintained the enrichment of ERRα on the promoters of *LAMP2* and *VAMP8*, while the acetylation-deficient mutant showed minimal accumulation on the promoters of the two genes (Figs. 6G). Rescued expression of the WT and KR mutant after ERRα knockdown also revealed that acetylation deficiency impaired the transactivation of ERRα on LAMP2 and VAMP8 (Figs. 6H). Non-acetylation in the KR mutant weakened its interaction with the co-activator PGC1β (Figs. 6I). Additionally, a KQ mutant of ERRα (lysine was mutated with glutamine to mimic the acetylation status) was constructed to perform protein stability assays. Cycloheximide chase and ubiquitination detection assays demonstrated that the KQ mutant had an insignificantly shorter life-time and stronger ubiquitination than the WT (Figure S4B-S4D). Reporter luciferase assays also showed that the KQ mutant had comparative transcriptional activity to WT ERRα (Figure S4E). Because KQ didn’t exhibited expectant stability and transcriptional activity, this study used the WT and KR mutants in the following functional investigation to elucidate the effect of acetylation on ERRα function in RCC cells. Overall, ERRα acetylation increased its ubiquitination and transcriptional activity in RCC cells.

### Acetylation of ERRα maintained the lysosome-dependent autophagy flux and promoted the tumorigenesis of RCC

**Fig. 7:**
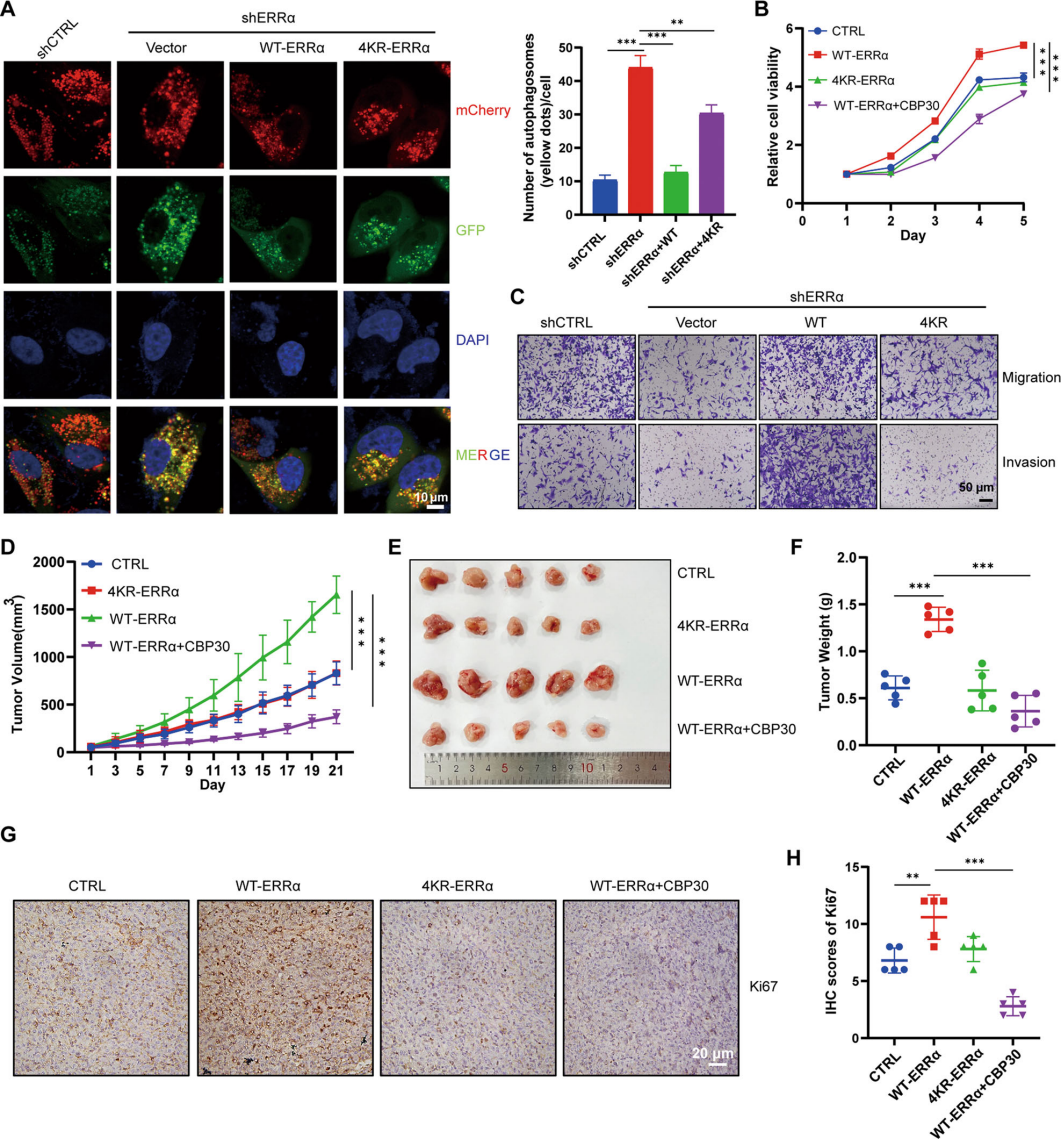
Acetylation of ERRα facilitated the tumorigenesis of RCC. **A** IF results showed that overexpression of WT ERRα but not 4KR could resuced the blockage effect of ERRα on autophagy flux in Caki-1 cells, column chart revealed the relative autolysosomes in different cells, ***p < 0.001, ns *p* *>* 0.05. **B** CCK8 assays evaluated the growth curves of OS-RC-2 cells overexpressed with Ctrl (Vector), WT, KR or WT treatment with CBP-30, ***p < 0.001. **C** Transwell assays tested the migration and invasion of OS-RC-2 cells from four groups indicated. **D–F** OS-RC-2 cells stably overexpressing the Ctrl (Vector), WT or KR were subcutaneously injected into mouse and one WT group were treated with CBP-30, the tumor volume were monitored every two days, the results were shown using with line chart (**D**), 21 days post injection, the mice were sacrificed and the tumors were removed, captured and weighed, the image and tumor weights were showed in **E** and **F**. Data were shown with means ± SD, *n* = 5 for each group, ***p < 0.001. **G** IHC assays revealed the expression of Ki67 in tumor tissues from four indicated groups. **H** Scatter diagram showed the IHC socres of Ki67 in four groups of mice. **p < 0.01, ***p < 0.001.

In terms of the protective role of autophagy in RCC cells and the regulation of acetylation on the functional performance of ERRα, the effect of ERRα acetylation on lysosome-dependent autophagy flux and progression of RCC were evaluated. As shown in Fig. 6H, the acetylation-deficient mutant could not maintain the expression of LAMP2 and VAMP8; therefore, we speculated that ERRα acetylation modulated the autophagy flux of RCC cells by governing the transcriptional activation of LAMP2 and VAMP8. Indeed, autophagy flux monitoring using mCherry-GFP-LC3B confirmed that the rescued expression of KR could not recover the ERRα knockdown–induced blockage on the fusion of autophagosomes with lysosomes (Figs. 7A). Functionally, WT ERRα overexpression significantly increased the proliferation of RCC cells, whereas the KR mutant showed only a weaker effect on cell growth (Figs. 7B). Treatment with CBP30, which largely repressed the p300/CBP-mediated ERRα acetylation (Figs. 5E), impaired the enhancing effect of WT ERRα (Figs. 7B). The rescued expression of WT ERRα also increased the migration and invasion of RCC, whereas the KR mutant showed a lesser effect (Figs. 7C). Moreover, mouse model showed that WT ERRα overexpression promoted the tumorigenesis of RCC cells in vivo, while the 4KR mutant had only a slight promotion effect on tumor growth, and WT ERRα coupled with CBP30 also impaired ERRα acetylation-mediated tumorigenesis of RCC cells (Figs. 7D–F). IHC staining results showed that WT ERRα had the highest expression of Ki67, LAMP2 and VAMP8, followed by 4KR, control, and WT + CBP30 (Figs. 7G, H and Figure S5A); these results indicated that ERRα maintained lysosome-dependent autophagy flux in vivo. Treatment with CBP30 did not result in any significant changes in mouse body weight (Figure S5B), indicating the excellent biocompatibility of CBP30 in vivo. Taken together, these results suggested that ERRα acetylation maintained the homeostasis of lysosome-autophagy pathway in RCC by enhancing its transactivation on LAMP2 and VAMP8.

### Pharmaceutical inhibition on ERRα acetylation impaired the lysosome-dependent autophagy flux and enhanced the sunitinib sensitivity of RCC

**Fig. 8:**
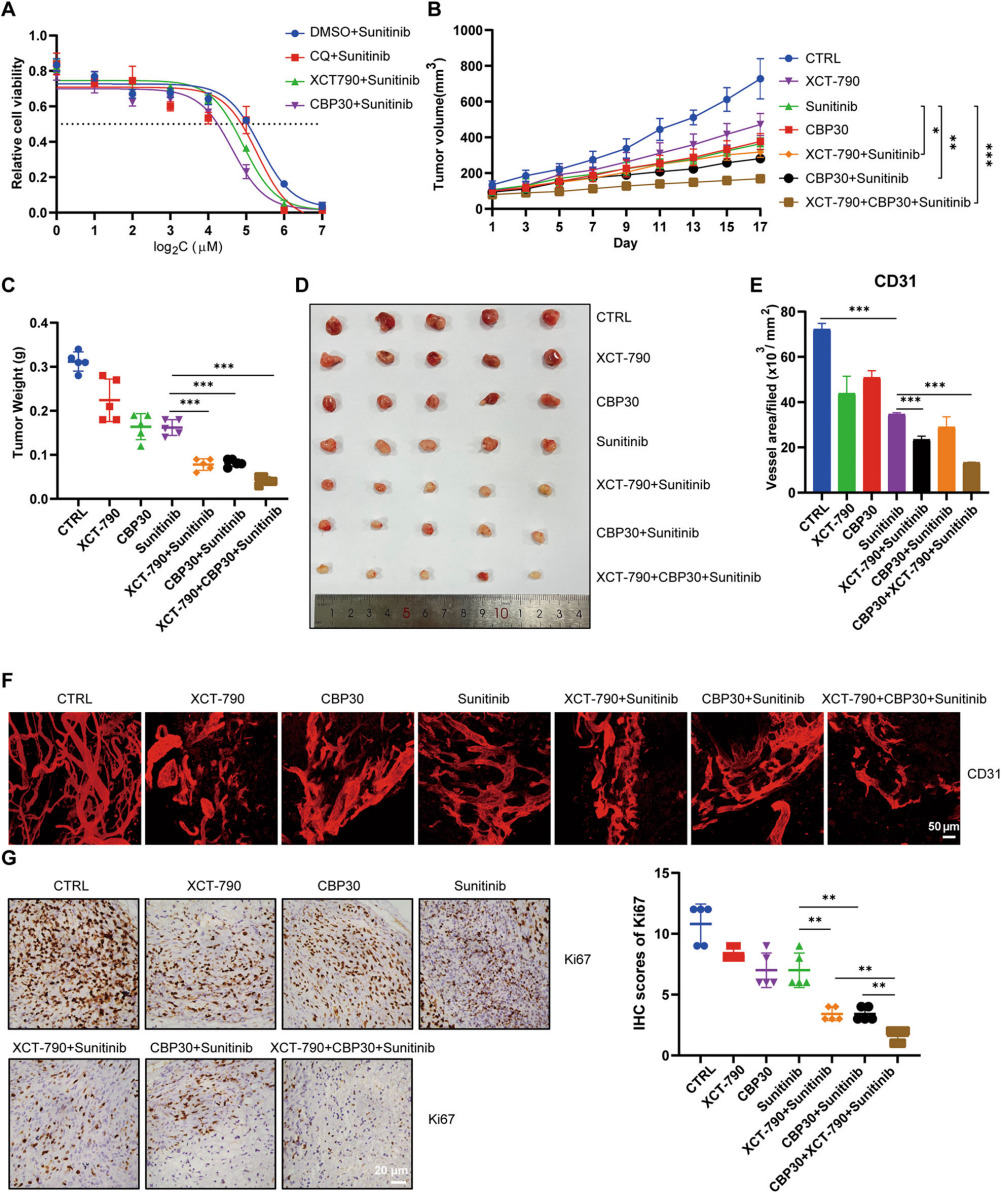
ERRα acetylation mediated autophagy-lysosome pathway regulated the sunitinib sensitivity of RCC. **A** CCK8 assay evaluated the IC50 of ACHN to the sunitinib with or without presence of CQ, XCT790 or CBP30. **B**–**D** The tumorigenesis of ACHN in vivo were measured using with mouse model, the 7 groups of mice were treated with solvent, CBP30, sunitinib, XCT790, suninib+XCT790, sunitinib+CBP30, XCT790 + CBP30+sunitinib. Tumor volume (**B**), tumor weights (**C**) and tumor image (**D**) of each groups were monitored and captured. The vessels in tumor tissues were staining with CD31 by whole-mount (**F**) and the relative area or intensity of vessels were statistically analyzed in column-bar chart (**E**). **G** The relative expression of Ki67was stained by IHC assays, scatter diagram showed the IHC scores of Ki67 in indicated groups. **p < 0.01.

Targeted therapy with the tyrosine kinase inhibitor sunitinib is the first-line choice for high-grade RCC, but drug resistance frequently occurs in the advanced stage of treatment [4]. Autophagy is reported to play a protective role in the survival of tumor cells under targeted therapy or chemotherapy [4, 6]; therefore, the role of the ERRα acetylation-mediated autophagy-lysosome pathway in the sunitinib sensitivity of RCC cells was evaluated. First, XCT-790 and CBP-30, the ERRα and p300/CBP inhibitors respectively, were introduced to determine the drug sensitivity of ACHN to sunitinib, and the results indicated that sunitinib combined with ERRα inhibition with XCT-790 decreased the IC_50_ of ACHN from 41.01 to 30.31 μM, and when combined with CBP-30, it decreased the IC_50_ to 24.79 μM (Figs. 8A). Furthermore, the tumor-repressing effects of sunitinib when combined with XCT-790 or CBP30 were evaluated in the mouse model. Tumor volume and weight monitoring revealed that inhibition of ERRα acetylation enhanced the pharmacological effect of sunitinib on tumor growth in vivo (Figs. 8B–D). Given the anti-angiogenic effect of sunitinib and the ERRα-VEGFA signaling on vessel regeneration, whole-mount staining assay for CD31 was performed and the results showed that XCT-790 and CBP30 had a synergistic repressive effect with sunitinib and exhibited an anti-angiogenic function (Figs. 8E, F). IHC results confirmed that inhibition of ERRα acetylation repressed the expression of Ki67 in tumor tissues (Figs. 8G), the expression of LAMP2 and VAMP8 were also inhibited by XCT-790 and CBP-30 (Figure S6A). Moreover, body weight of mice did not showed evident side-effects of combined therapy in vivo (Figure S6B). In conclusion, these data indicated that inhibition of ERRα acetylation reinforced the antitumor effect of sunitinib by impairing the protective autophagy-lysosome pathway in RCC cells.

## Discussion

ERRα, an orphan nuclear receptor, acts as a vital regulator of various events in solid tumors including breast cancer, prostate adenocarcinoma, cervical cancer, and hepatocellular carcinoma, and its expression and activity are largely correlated with tumor aggression and pathological stage [25]. Selective inhibitors and natural small-molecule inhibitors of ERRα, such as XCT-790, C29 and kaempferol, exhibit excellent antitumor and anti-angiogeneic effects both in vitro and in vivo [15]. However, the function of ERRα in RCC has not been fully investigated. The present study demonstrated that ERRα plays an oncogenic role and facilitates the proliferation and invasion of RCC cells, and knockdown or pharmaceutical inhibition of ERRα induced remarkable growth regression in RCC cells, and caused a decrease of vessel intensity in tumor tissues. More importantly, treatment with XCT-790 in mice did not show any evident side effects, indicating that targeting ERRα might be an applicable strategy for the clinical therapy of RCC.

The autophagy-lysosome pathway participates in many important physiological and pathological events, and its dysfunction occurs in intractable diseases including neurodegenerative disorders, amyotrophic lateral sclerosis, and malignancies [26]. Regarding tumor progression, an equilibrium between autophagy flux and lysosomal function is vital to the survival and drug resistance of cancer cells, and blockage on the autophagy-lysosome pathway is a promising strategy in cancer therapy [8]. However, directly inhibition of key factors such as ATG5, ATG7, or LAMP1 may cause serious side effects and systemic toxicity in patients [38]. Therefore, it is valuable to identify an operable drug-targeting biomarker that can repress the aberrant activity of the autophagy-lysosome pathway in carcinomas. In the present study, proteomics and mechanistic investigation uncovered that ERRα modulates autophagy flux by maintaining the fusion of autophagosomes with lysosomes in RCC cells, characterized by abnormal activation of autophagy corresponding to its high metabolic activity and substance cycling. As for detailed regulation, ERRα directly bonded to the promoter regions and consequently enhanced the transcription of *LAMP2* and *VAMP8*, two important factors that modulate the fusion of autophagosomes with lysosomes in the late stage of autophagy flux [28]. Consistently, immunofluorescence assay and western blot assay results confirmed that ERRα knockdown induced a decrease in the fusion of autophagosomes with lysosomes and increased the accumulation of SQSTM1/p62 proteins (Figs. 2D–F). Although ERRα was previously reported to regulate the innate host defense and clearance of misfolded protein aggregates through modulating autophagy, the conclusion are inconsistent with each other. Kim et al. reported that ERRα operated with SIRT1 and formed a feed-forward loop in macrophages under *Mycobacterium tuberculosis* infection, which was required for autophagosome formation by enhancing the transcriptional activation of Atg5, Becn1, and Atg16l1 [39]. While Suresh et al. and Zhang et al. demonstrated that ERRα inhibited autophagosome formation in a AMPK/mTOR dependent manner, and ERRα inhibition by XCT-790 cleared toxic protein aggregates by inducing autophagy as demonstrated in a Parkinson’s disease mouse model [40, 41]. Therefore, the defined function of ERRα in autophagy might depend upon the specific cellular environment and crosstalk between signaling pathways.

This study also revealed that ERRα knockdown–mediated dysfunction of autophagy flux caused growth repression of RCC, and impairment of the protective autophagy restored the sensitivity of RCC cells to sunitinib, a first-line drug used for treating high-grade RCC, these findings indicated that ERRα might contribute to the survival and drug resistance of cancer cells under therapeutic stress, which was consistent with the previous studies [36, 42, 43]. Therefore, uncovering the regulatory mechanism modulating ERRα activity in a specific cellular environment and finding suitable molecules to repress its action will provide more confidence in applying the strategy for tumor therapy.

The present study also revealed a crosstalk between PTMs and ERRα activity in RCC. We found that the stability and transcriptional activity of ERRα were tightly modulated by acetylation and ubiquitination. On the one hand, hypoxic microenvironment of RCC triggered a higher degree of acetylation and transcriptional activity of ERRα through inducing a stronger interaction between p300/CBP and ERRα. p300/CBP-mediated acetylation of ERRα at the DBD (K100, K125, K138, and K146) promoted its nucleus transition, affinity toward targeting DNA and enhanced the transcriptional performance, which maintained the autophagy flux and tumorigenesis of RCC cells by increasing the expression of LAMP2 and VAMP8 (Figs. 6G, H and S4A). On the other hand, hypoxia triggered dynamic acetylation increased the ubiquitination and degradation of ERRα, this phenomenon was seemingly contradictory to the increased transcriptional activity of ERRα induced by acetylation (Figs. 4J and 6C). Similar results were obtained with its structurally similar protein, ERα [44]. ERα can exhibit transactivation and its enrichment on the promoters of targeting genes partially dependent on its ubiquitin E3 ligase-mediated ubiquitination-dependent degradation [45], which facilitates the circular occupation of ERα on the promoters and achieves sustained transcriptional activation of the genes [46]. And we found that hypoxia could increase the nuclear location of Parkin and the interaction between Parkin and ERRα (Figs. 4J and S4A), and hypoxia treatment triggered higher acetylation and ubiquitination of ERRα in nucleus (Figure S4A). Based on ERRα shares high similarity with ERα in terms of structure and functional modeling [9], and Shires et al. reported that Parkin could response to hypoxia and translocate into nucleus, then facilitated ERRα ubiquitination coupled with increased its transcriptional activity [37], we speculated that acetylated ERRα might be a transcriptionally active form, this active form in the nuclear had higher affinity with DNA and needed faster circular occupation on the promoters of targeting genes, Parkin-mediated ubiquitination and degradation could achieved the continuous transactivation of ERRα. Functional investigation also showed that the blockade of ERRα acetylation resulted in a synergetic effect with sunitinib, and this treatment effectively repressed the tumorigenesis and angiogenesis of RCC. Taken together, the results indicate that detection of the active form of ERRα such as acetylated ERRα, but not total raw protein levels, might have a better clinical significance in RCC diagnosis, and inhibition of the ERRα acetylation-mediated autophagy-lysosome pathway could enhance the targeting therapy efficiency of sunitinib. However, applying it into RCC diagnosis and determining the efficiency of this promising treatment requires more clinical testing.

In addition, our proteomics data revealed that multiple metabolism pathways (such as carbon metabolism, amino acids biogenesis, and pyrimidine metabolism) and apoptosis were associated with ERRα in RCC (Figs. 2B and S7A), so we verified many metabolic enzymes and apoptosis-related proteins using WB and IHC. The results found that shERRα decreased MDH2 and ACO2 expression, and increased the levels of IDH3B, CTSB, and Cytochrome C in RCC cells and tumor tissues (Figure S7B and S7C), and promoter analysis revealed that ERRα might directly bind to promoter regions and modulate transcription of CTSB, Cytochrome C, ACO2 and MDH2 (Figure S7D). As these proteins played vital role in TCA cycle, OXPHOS and electron transportation in mitochondria, these results indicated that ERRα regulated energetic and material metabolism through multiple pathways (Figure S7E). And ERRα was reported to regulate mitochondrial biogenesis and function through PGC1-α [47], metabolism reprogramming and mitochondrial function are essential for tumorigenesis and metastasis of RCC [48], so ERRα-mediated metabolism might be meaningful target of RCC. Moreover, we also evaluated the effect of ERRα on the apoptosis using flow cytometry and the results found that shERRα induced slightly increase in apoptotic rates but not caused large scale of cell death (Figure S7F), which was consistent with the increase expression of Cytochrome C and CTSB. Disruption on autophagy-lysosome pathway and metabolism homeostasis by shERRα mighty induced this pro-apoptosis effect in RCC. Moreover, ERRα have been wide documented to crosstalk with hypoxia/HIFs signaling in different carcinomas through directly interacting with HIFs to increase HIFs stability or activating hypoxia-related genes in HIFs-independent manner [49], but whether ERRα could interact with HIFs is unclear, we performed IP in Caki-1 and didn’t detect the interaction between ERRα and HIF-2α (data not shown), but we found that VEGFA intensity were largely decreased in the tumor tissues of shERRα (Figure S7G), indicating that ERRα might participate with hypoxia signaling in HIFs-independent manner. More research are deserved to be conducted to resolve this issue. Another issue needs to be investigated is that we mainly focused on the ERRα/PGC1β in our model, ERRα could also interact with PGC1α (Figure S7H), whether ERRα prefer to interact with PGC1α or PGC1β, these two types of combination exhibit same or different transcriptional function in ALP and RCC, it needs more comprehensive investigation in the future. What’s more, the specific deacetylase responsible for ERRα deacetylation in RCC is worth to be explored.

In conclusion, our data identified a novel mechanism that the hypoxic microenvironment of RCC enhanced p300/CBP-mediated acetylation of ERRα and triggered an increased transcriptional performance of ERRα on the transactivation of LAMP2 and VAMP8, which maintained lysosome-dependent autophagy flux and the fusion of autophagosomes with lysosomes in RCC cells (Figure S8). Pharmaceutical inhibition of this activity caused growth repression and enhanced sunitinib sensitivity in RCC cells. Therefore, the ERRα acetylation-mediated autophagy-lysosome pathway presents a promising diagnostic biomarker and therapeutic target for RCC.

## Supplementary information

Supplementary Figures

Supplementary Table 1-5

Supplementary Table 6

Supplementary Table 7

Original images of WB

## Data Availability

Raw and processed proteomics data from this study were deposited in ProteomeXchange (http://www.proteomexchange.org/) via PRIDE dataset, the project accession number was PXD041739. The original data or materials used in this study are available upon reasonable request from the corresponding author.
