# Correction: Hypoxia-triggered ERRα acetylation enhanced its oncogenic role and promoted progression of renal cell carcinoma by coordinating autophagosome-lysosome fusion

**DOI:** 10.1038/s41419-025-07493-4

**Published:** 2025-04-04

**Authors:** Chun Feng, Demin Kong, Binghua Tong, Yonghui Liang, Fuyi Xu, Yangyang Yang, Yingying Wu, Xiaodong Chi, Pengfei Wei, Yang Yang, Guilong Zhang, Geng Tian, Zhaowei Xu

**Affiliations:** 1 Shandong Technology Innovation Center of Molecular Targeting and Intelligent Diagnosis and Treatment, School of Pharmacy, Binzhou Medical University, Yantai, China; 2 The Second Medical College, Binzhou Medical University, Yantai, China; 3 School of Basic Medicine, Binzhou Medical University, Yantai, China

**Keywords:** Macroautophagy, Transcription factors, Renal cell carcinoma

Correction to: *Cell Death and Disease* 10.1038/s41419-025-07345-1, published online 16 January 2025

After the completion of this study, we proceeded to implement an archiving initiative for the pertinent experimental raw data. However, while organizing the original data presented in Fig. S1C, we found that an unrelated image had unintentionally been used in the group of shERRα #3 in 0 h when organizing figures due to a copy-pasting error among the original images. The correct figure that was generated from this experiment have been provided in the revised supplementary figures in the published version of the article.

Corrected Supplementary Figure 1
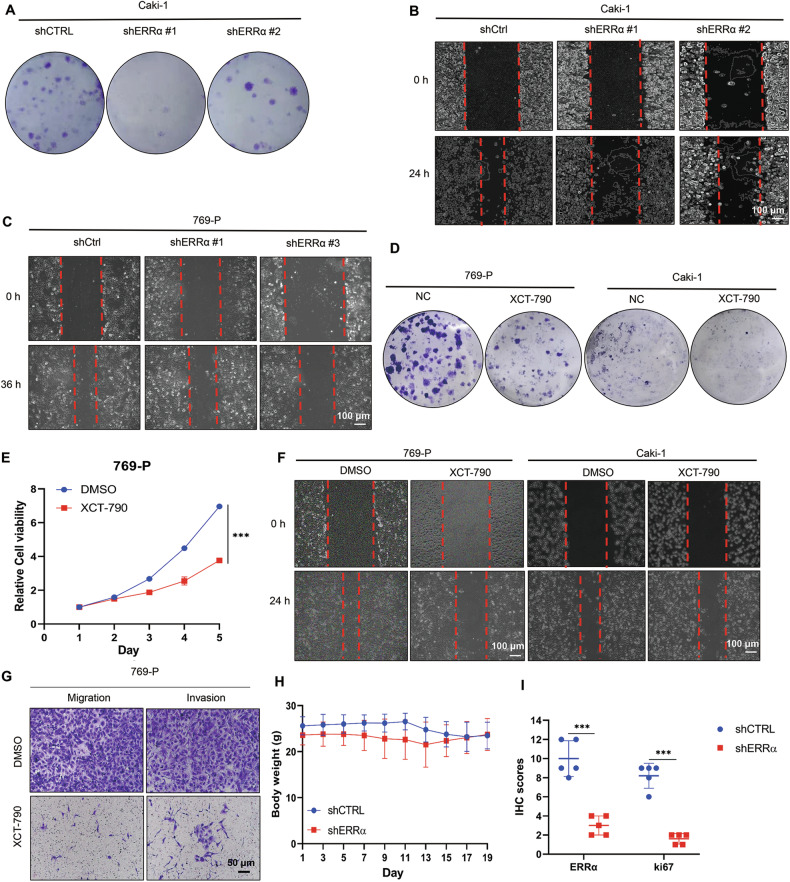


Original Supplementary Figure 1
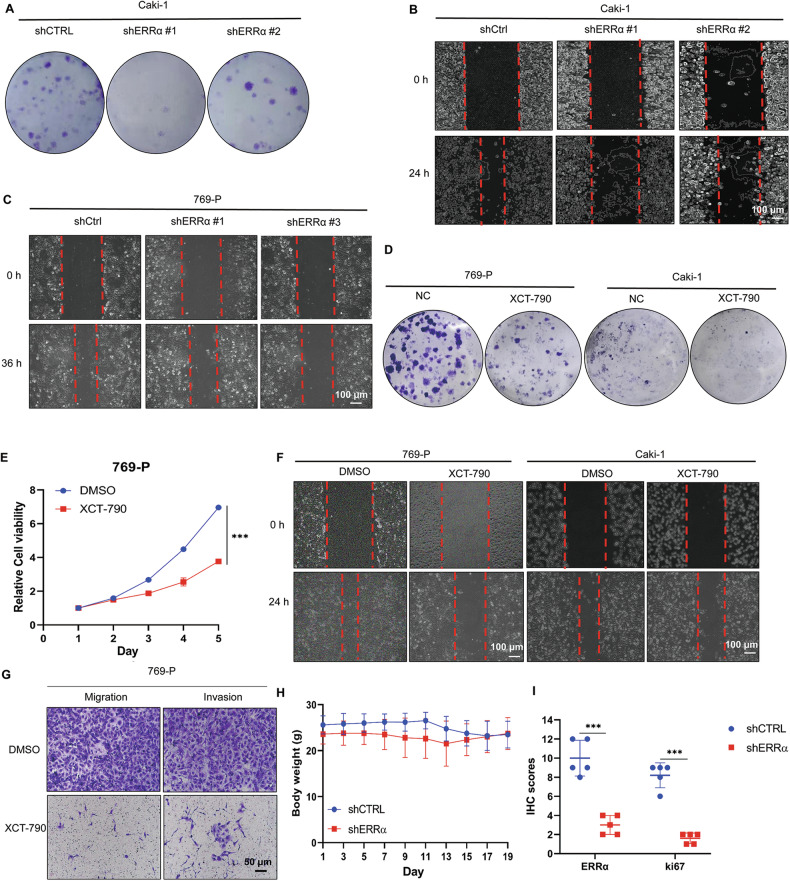


The original article has been corrected.

