# Supplementary material for: Hypoxia-triggered ERRα acetylation enhanced its oncogenic role and promoted progression of renal cell carcinoma by coordinating autophagosome-lysosome fusion
Source: Cell Death Dis. 2025 Jan 16;16(1):23. doi: 10.1038/s41419-025-07345-1 (PMC11739407; doi:10.1038/s41419-025-07345-1)
Supplement: Supplementary file 1 — Supplementary Figures [file 41419_2025_7345_MOESM1_ESM.docx]

**Hypoxia-triggered ERRα acetylation enhanced its oncogenic role and promoted progression of renal cell carcinoma by coordinating autophagosome-lysosome fusion**

**Running title:** Role of ERRα acetylation in renal cell carcinoma.

Chun Feng^1,2^, Demin Kong^1^, Binghua Tong^1^, Yonghui Liang^1^, Fuyi Xu^1^, Yangyang Yang^3^, Yingying Wu^1^, Xiaodong Chi^1^, Pengfei Wei^1^, Yang Yang^1^, Guilong Zhang^1*^, Geng Tian^1*^, Zhaowei Xu^1*^

^1^Shandong Technology Innovation Center of Molecular Targeting and Intelligent Diagnosis and Treatment, School of Pharmacy, Binzhou Medical University, Yantai, China

^2^ The Second Medical College, Binzhou Medical University, Yantai, China

^3^ School of Basic Medicine, Binzhou Medical University, Yantai, China

**Corresponding authors：**

Zhaowei Xu, E-mail: [zhaoweixv@bzmc.edu.cn](mailto:zhaoweixv@bzmc.edu.cn). ORCID: 0000-0001-8608-576X. Guilong Zhang, E-mail: [glzhang@bzmc.edu.](mailto:glzhang@bzmc.edu.)cn. Geng Tian, E-mail: [tiangengbmu@163.com](mailto:tiangengbmu@163.com). Address: School of Pharmacy, Binzhou Medical University, No. 346 Guan Hai Road, Lai Shan Zone, Yantai, Shandong Province, 264003, China. Tel: 86-535- 6913395

**Supplementary figures**


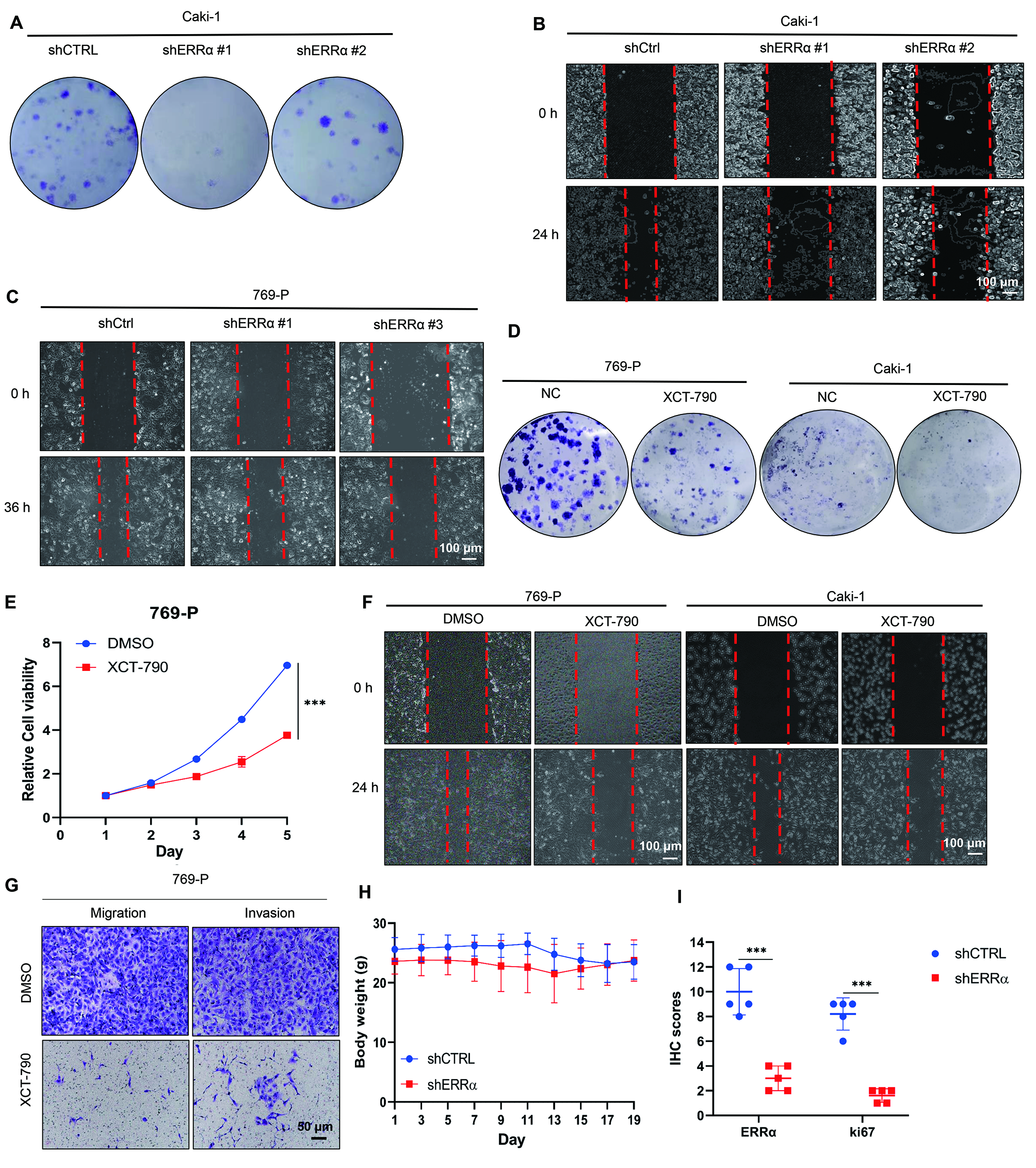


**Figure S1. (A)** Colony formation assay showed the effect of shERRα or shCTRL on the colony number of Caki-1. **(B and C)** Scratch wound-healing assays were performed to investigate the effect of ERRα knockdown on the migration of Caki-1 (B) and 769-P (C). **(D)** The colony formation assay were conducted in 769-P and Caki-1 to explore the inhibition of XCT-790 on the proliferation of RCC cells. **(E)** The growth curves of 769-P treated with DMSO or XCT-790 were measured by CCK8, ****p* < 0.001. **(F and G)** The inhibitory efficiency of XCT-790 on migration and invasion were determined by scratch wound-healing (F) and transwell assays (G), respectively. **(H)** Line chart was drawn to show the body weight changes of mice from two indicated groups. **(I)** Scatter diagram showed the IHC socres of ERRα and Ki67 in indicated groups. ****p* < 0.001.

.


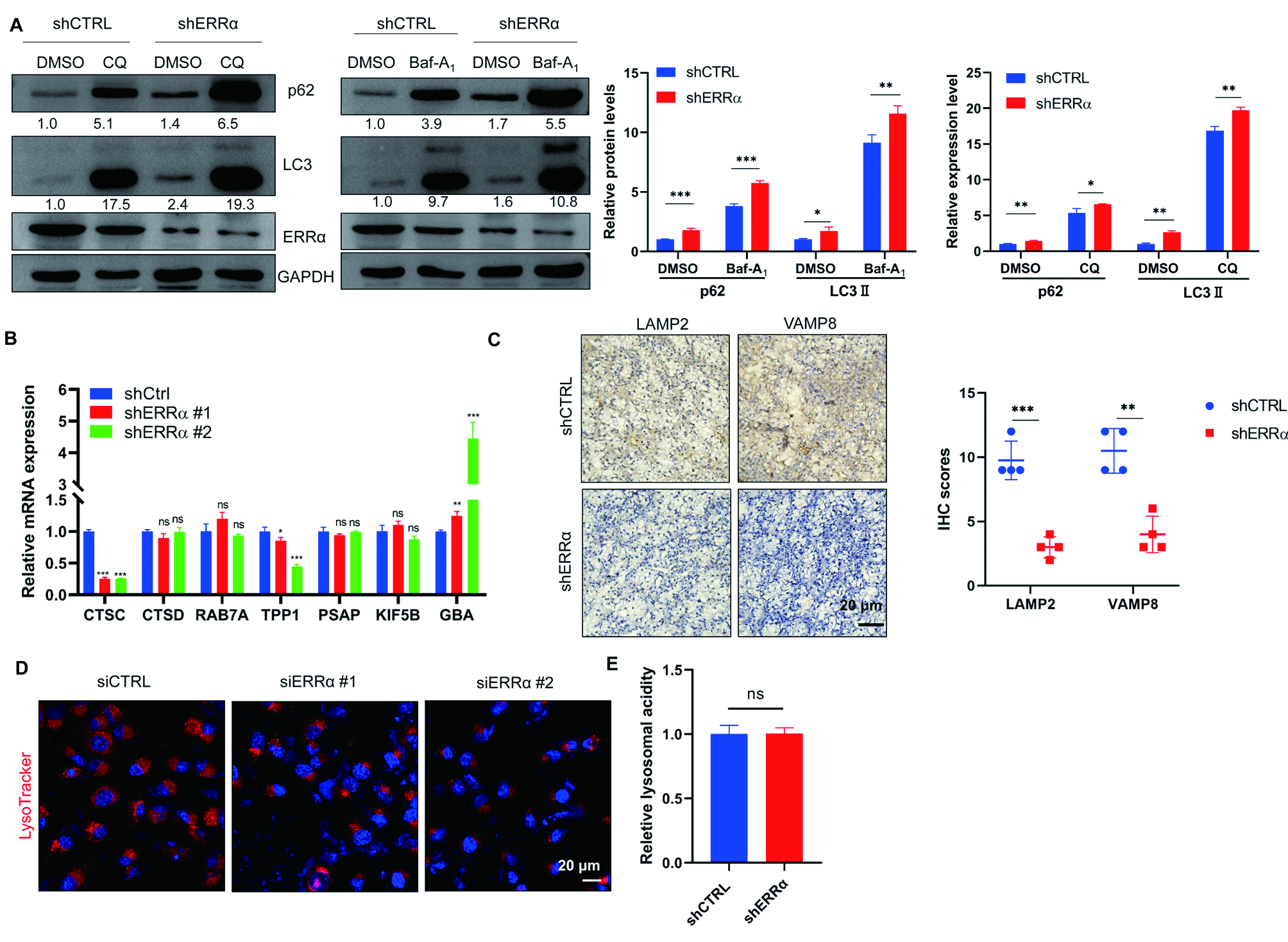


**Figure S2.** **(A)** The expression of p62 and LC3 were determined by Western blot in Caki-1 with shCTRL and shERRα, the autophagy flux were blocked with CQ or Baf-A_1_.Column graph showed the relative protein levels (gray values of bands normalized by GAPDH) in indicated groups, data were showed with mean±SD that came from three independent repeats, **p* < 0.05, ***p* < 0.01, ****p* < 0.001. **(B)** qPCR assay evaluated the mRNA levels of lysosome associated genes with or without ERRα knockdown in Caki-1, **p* < 0.05, ***p* < 0.01, ****p* < 0.001. **(C)** IHC assays evaluated the expression of LAMP2 and VAMP8 in tumor tissues from indicated groups. ***p* < 0.01, ****p* < 0.001. **(D)** Confocal images showed a decreased population of lysosome in ERRα knockdown RCC cells. **(E)** Lysosomal acidification in Caki-1 with shCTRL or shERRα were determined by LysoSensor Yellow/Blue DND-160 probe.


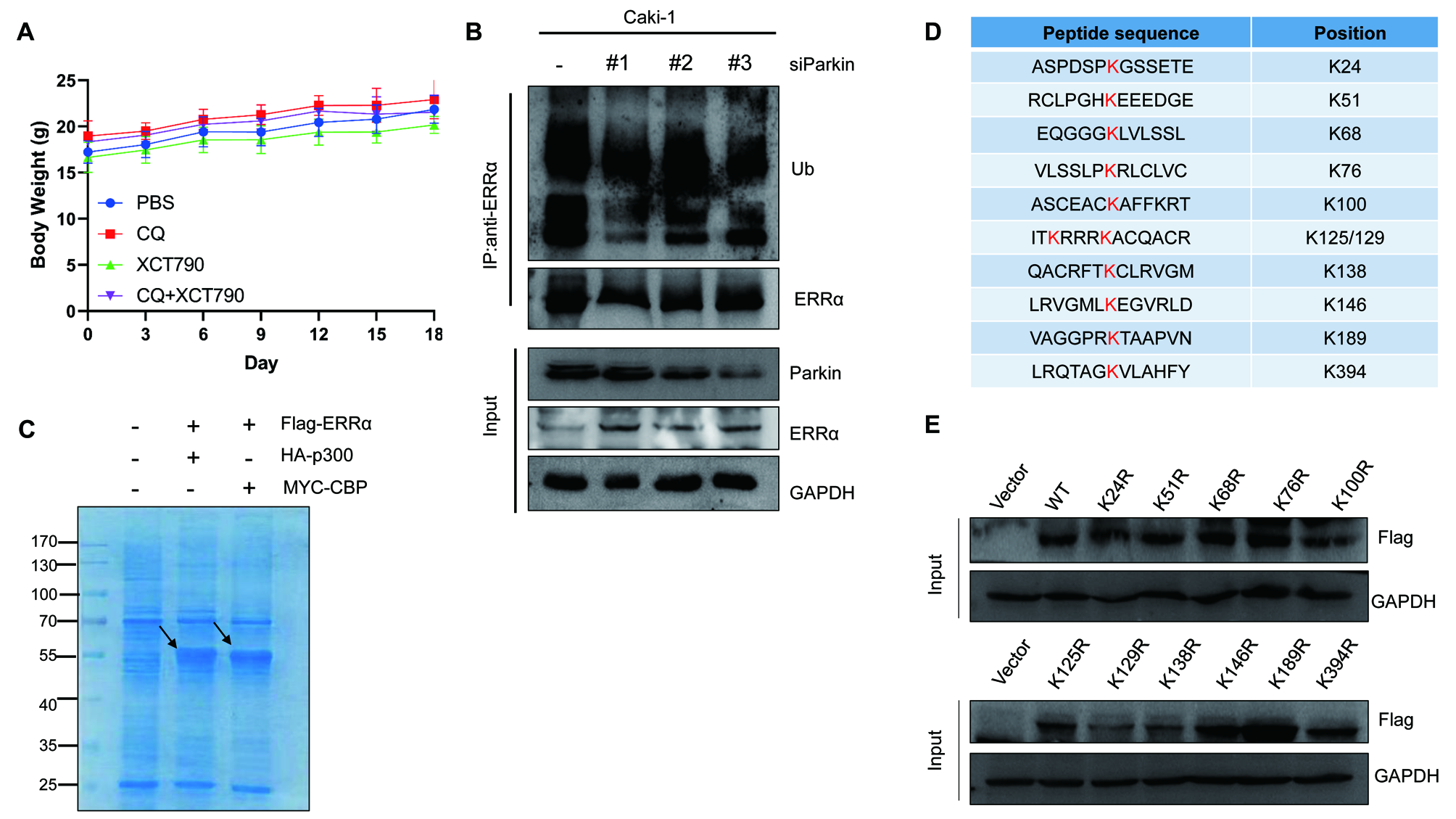


**Figure S3. (A)** The line chart showed the body weights of mice treated with indicated different inhibitors. **(B)** IP assays were conducted to evaluate the ubiquitination of ERRα with or without Parkin knockdown. **(C)** Coomassie brilliant blue staining showed the bands of acetylated ERRα purified IP. **(D)** The acetylated sites and peptides of ERRα identified by MS were listed as a table. **(E)** Western blot showed the protein expression of WT or single lysine mutated ERRα corresponding to the Input of IP assay mentioned in Figure 5G.


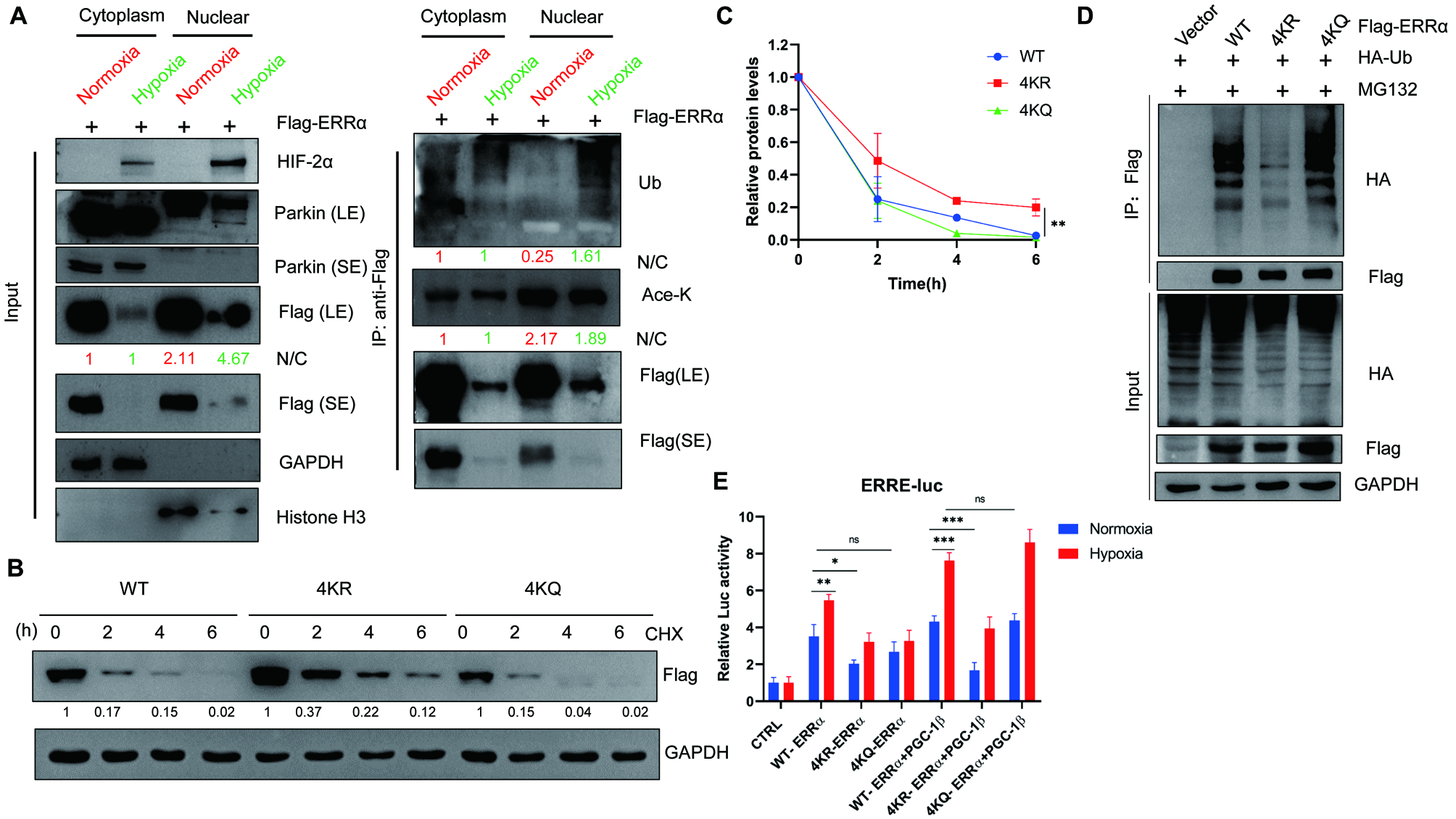


**Figure S4.** **(A)** IP were conducted in cytoplasmic and nuclear fraction revealed that the effect of hypoxia on the subcellular location, acetylation and ubiquitination of ERRα. N/C means the ratio of relative protein intensity in nucleus and cytoplasm, the protein levels in cytoplasm was normalized as 1. Red and green numbers indicated the data in normoxia and hypoxia groups respectively. **(B)** Cycloheximide chase assays revealed the different half-life times of WT, 4KR and 4KQ in presence of CHX. **(C)**The line chart showed the relative protein levels of three constructions of ERRα. **(D)** The ubiquitination of WT, 4KR and 4KQ were detected with IP assay. **(E)** The transcriptional activity of WT, 4KR and 4KQ on the ERRE luc were determined under hypoxic and normoxic conditions. **p* < 0.05, ***p* < 0.01, ****p* < 0.001, ns *p*＞0.05.


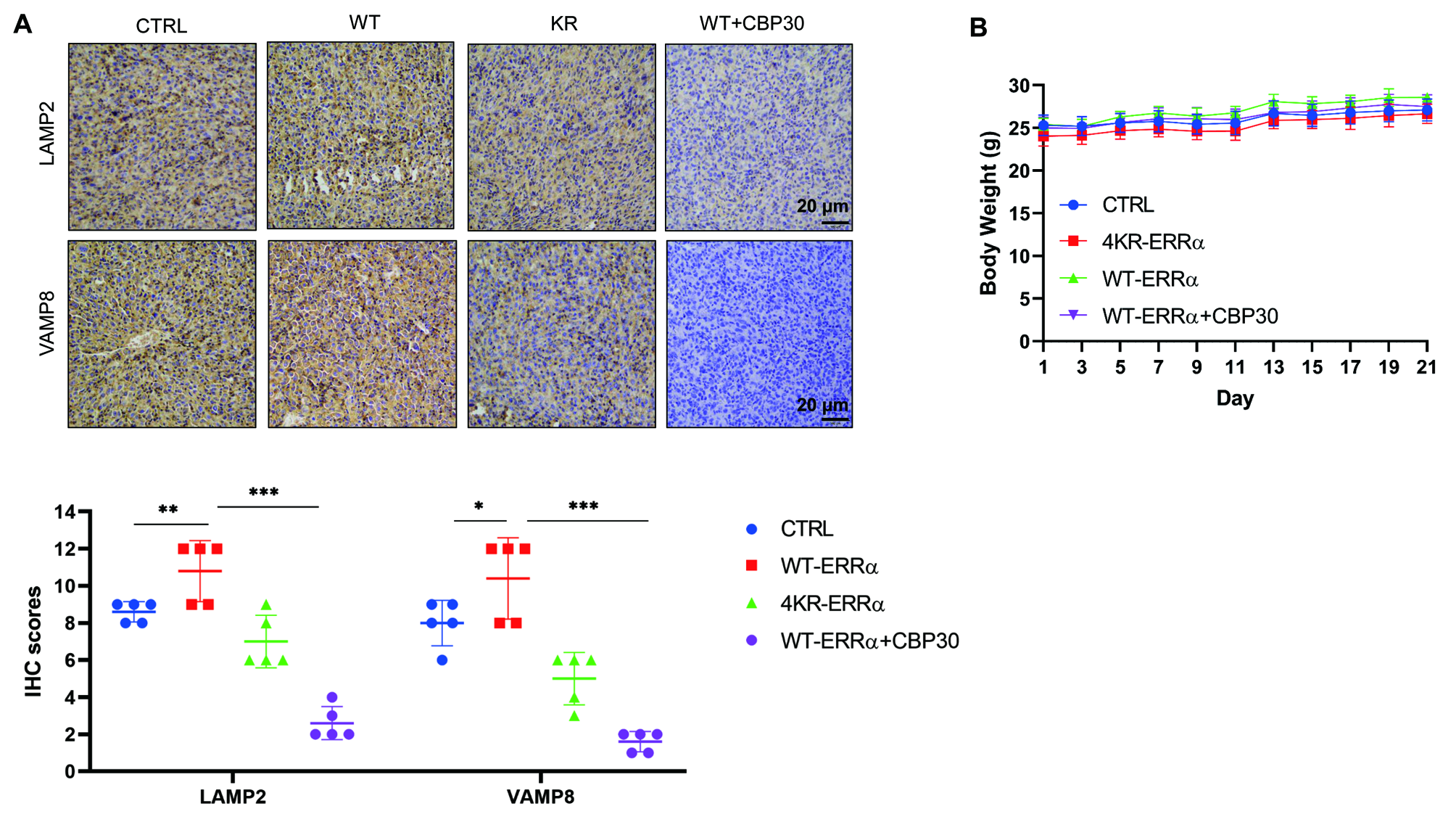


**Figure S5.** **(A)** IHC assay showed the relative intensity of LAMP2 and VAMP8 in four indicated groups. Scatter diagram showed the IHC socres of ERRα and Ki67 in indicated groups. **p* < 0.05, ***p* < 0.01, ****p* < 0.001, ns *p*＞0.05. **(B)** Line chart elucidated the body weights of four indicated groups of mice. **(C)** Line chart elucidated the body weights of seven indicated groups of mice.
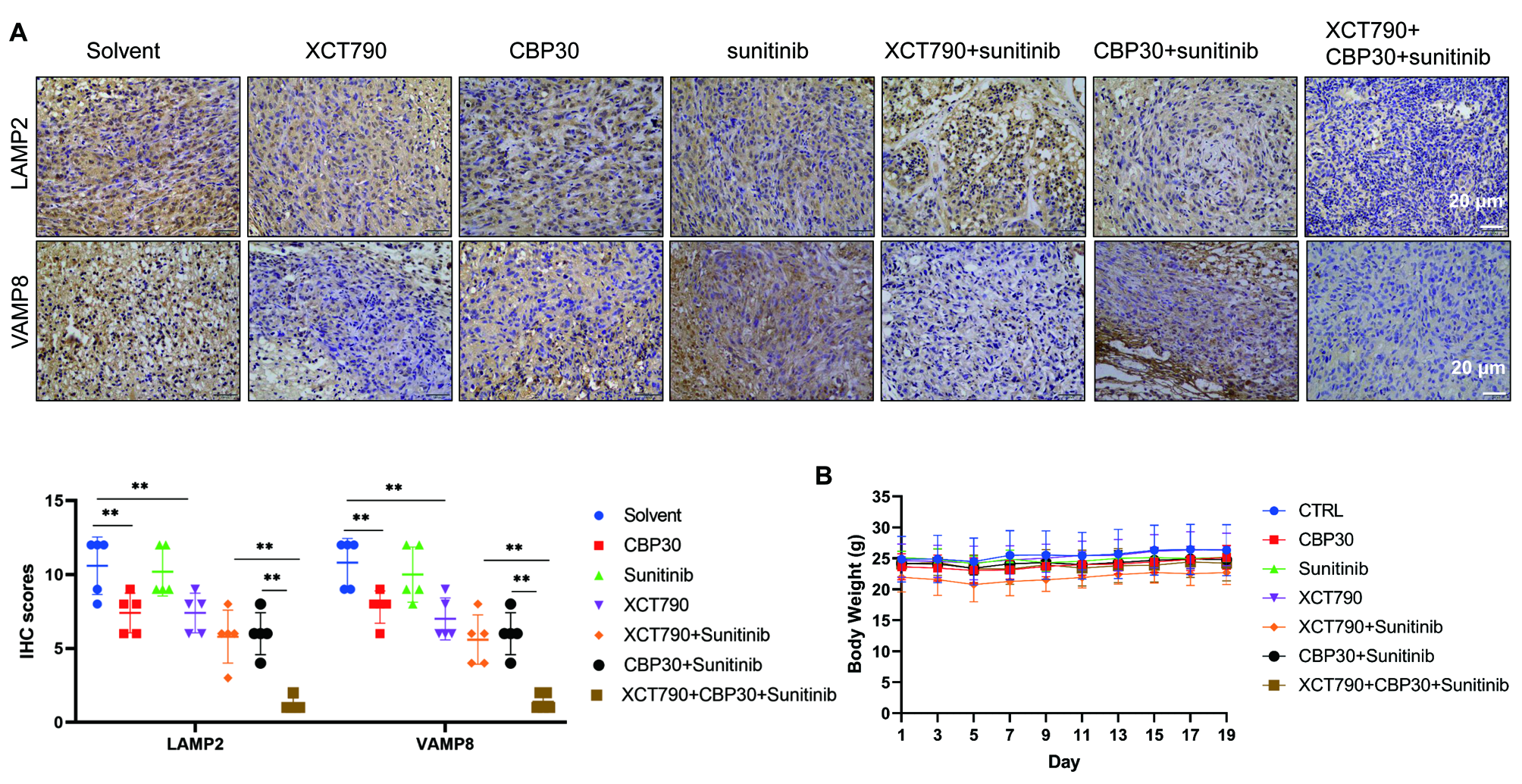


**Figure S6.** **(A)** IHC assays evaluated the expression of LAMP2 and VAMP8 in tumor tissues from indicated groups. ***p* < 0.01. **(B)** Line chart elucidated the body weights of seven indicated groups of mice.


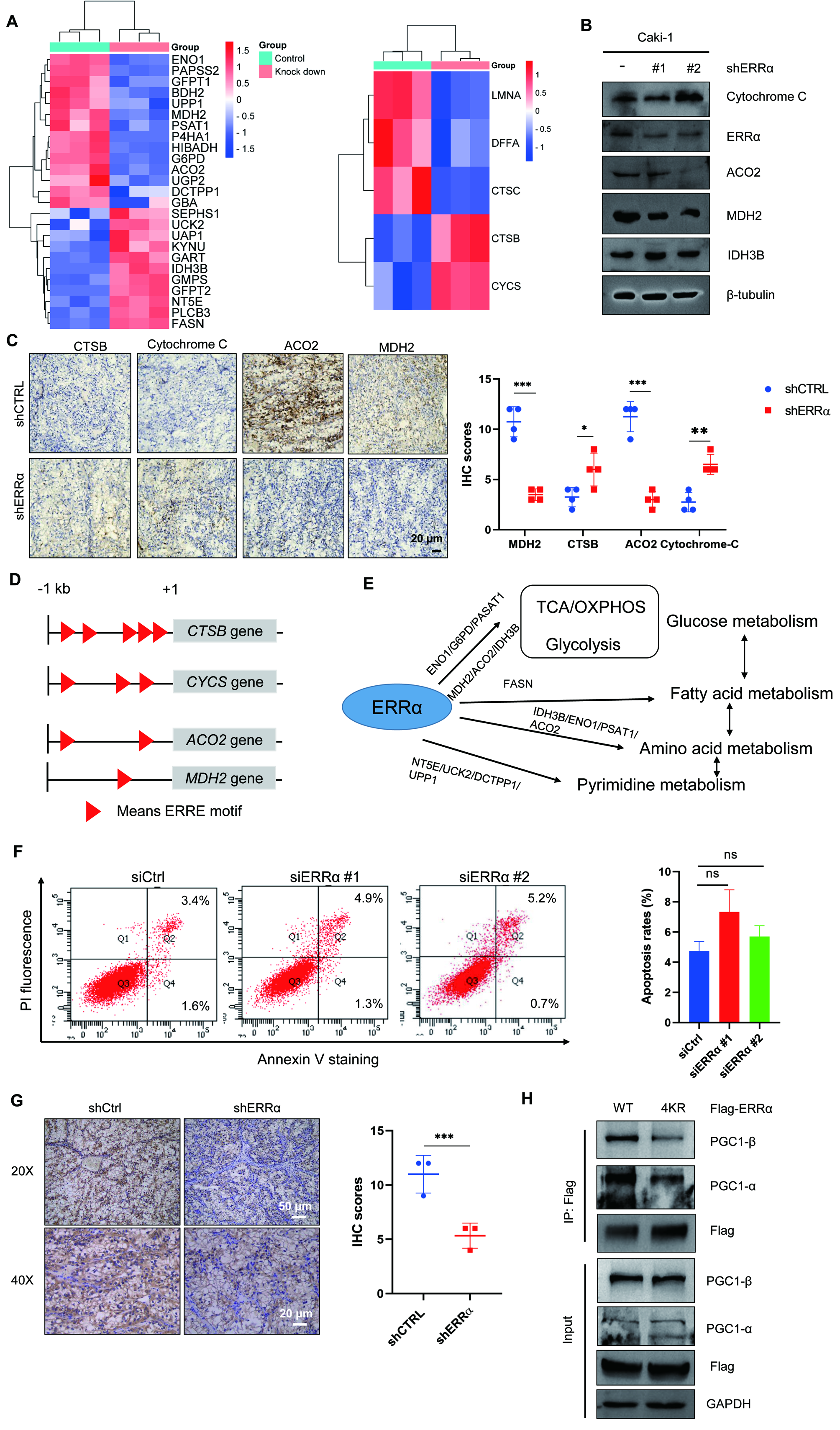


**Figure S7.** **(A)** The heatmaps showed the expression levels of metabolism- and apoptosis-related DEPs in Caki-1 with or without ERRα knockdown. **(B)** Western blot were conducted to detect the protein levels of Cytochrome C, ACO2, MDH2, and IDH3B in Caki-1 with or without shERRα. **(C)** The intensity of ACO2, MDH2, IDH3B, CTSB, and Cytochrome C in tumor tissues were determined by IHC. **p* < 0.05, ***p* < 0.01, ****p* < 0.001. **(D)** Promoter sequence analysis revealed the potential binding sites in *CTSB, CYCS, MDH2*, and *ACO2* genes. **(E)** ERRα regulated metabolism homeostasis through modulating multiple pathways. **(F)** Annexin V/PI staining evaluated the effect of shERRα on apoptosis of Caki-1. ns p＞0.05. **(G)** IHC results showed that shERRα significantly decreased the intensity of VEGF in tumor tissues. ****p* < 0.001. **(H)** The interaction between endogenous PGC1-α, PGC1-β and exogenous WT or 4KR ERRα were determined by IP.


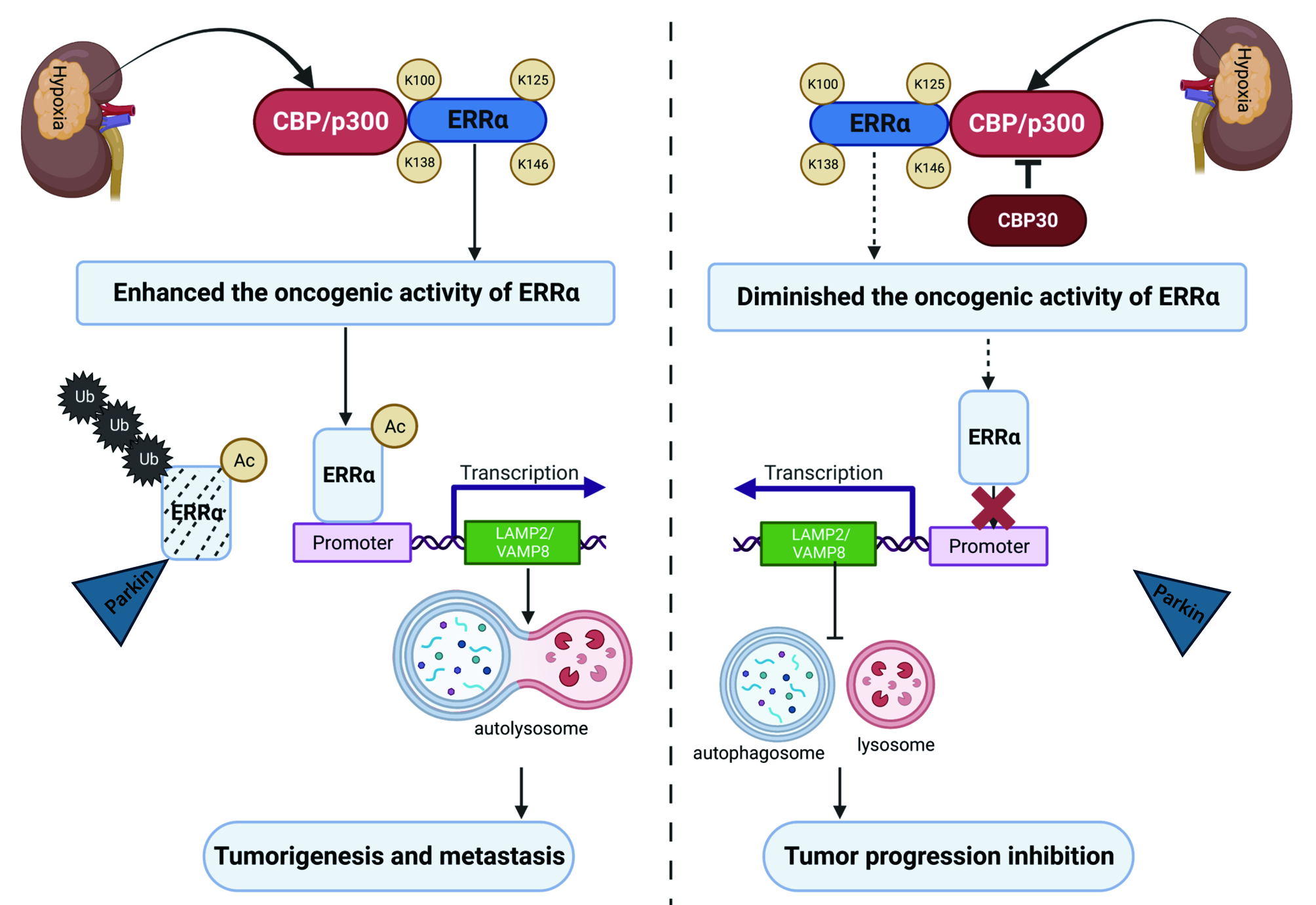


**Figure S8.** Mechanism diagram showed the regulatory role of ERRα on the autophagy flux and tumorigenesis of RCC cells.
