# Supplementary material for: Hypoxia-triggered ERRα acetylation enhanced its oncogenic role and promoted progression of renal cell carcinoma by coordinating autophagosome-lysosome fusion
Source: Cell Death Dis. 2025 Jan 16;16(1):23. doi: 10.1038/s41419-025-07345-1 (PMC11739407; doi:10.1038/s41419-025-07345-1)
Supplement: Supplementary file 2 — Supplementary Table 1-5 [file 41419_2025_7345_MOESM2_ESM.docx]

**Table S1.** Antibodies used in the experiments

| Antibody | Catalog # | Supplier | Application |
| --- | --- | --- | --- |
| ERRα | 13826S | Cell Signaling Technology | WB (1:1000) |
| GAPDH | AC033 | Abclonal | WB (1:1000) |
| LAMP2 | DF6719 | Affinity | WB (1:1000) |
| LC3 | 12741S | Cell Signaling Technology | WB (1:1000) IF (1:200) |
| Ace-K | 9441S | Cell Signaling Technology | WB (1:1000) |
| GFP | AE078 | Abclonal | WB (1:1000) |
| Ub | 10201-2-AP | Proteintech | WB (1:1000) |
| Flag (M) | AE005 | Abclonal | WB (1:2000) |
| HA | M20003 | Abmart | WB(1:2000) |
| MYC | 16286-1-AP | Proteintech | WB (1:1000) |
| p300 | 54062S | Cell Signaling Technology | WB (1:1000) IP (1:100) |
| CBP | WL02824 | Wanlebio | WB (1:1000) IP (1:100) |
| PGC1β | 22378-1-AP | Proteintech | WB (1:1000) |
| PGC1α | 66369-1-Ig | Proteintech | WB (1:1000) |
| IDH3B | PS06914 | Abmart | WB (1:1000) |
| ACO2 | 11134-1-AP | Proteintech | WB (1:1000) IHC (1:100) |
| MDH2 | 15462-1-AP | Proteintech | WB (1:1000) IHC (1:150) |
| Cytochrome C | T55734 | Abmart | WB (1:1000) |
| Parkin | 14060-1-AP | Proteintech | WB (1:1000) |
| HIF-2α | 59973S | Cell Signaling Technology | WB (1:1000) |
| VAMP8 | A4728 | Abclonal | WB (1:1000) |
| p62 | 8025S | Cell Signaling Technology | WB (1:1000) |
| Flag (R) | F9291 | Sigma-Aldrich | CHIP (1:100) |
| Flag (R) | 20543-1-AP | Proteintech | WB (1:1000) |
| ERRα | Ab137489 | Abcam | IHC (1:200) |
| ERRα | ab76228 | Abcam | IP (1:100) |
| Ki67 | 27309-1-AP | Proteintech | IHC (1:200) |
| VEGFA | 19003-1-AP | Proteintech | IHC (1:200) |
| CD31 | AF3628 | Biotechne | whole-mount (1:200) |
| LAMP2 (M) | 66301-1-1g | Proteintech | IF (1:200) |
| CTSB | YT0679 | Immunoway | WB (1:1000) |
| HRP-Anti-Rabbit | 7074 | Cell Signaling Technology | WB (1:10000) |
| HRP-Anti-Mouse | 7076 | Cell Signaling Technology | WB (1:10000) |
| Alexa Fluor 488-labeled Goat Anti-Mouse IgG | 615-545-214 | Jackson ImmunoResearch | IF (1:1000) |
| Alexa Fluor 647-labeled Goat Anti-Rabbit IgG | 111-605-144 | Jackson ImmunoResearch | IF (1:1000) |

**Table S2**. The list of reagents

| Inhibitor | Code | Manufacturer |
| --- | --- | --- |
| XCT-790 | S0407 | Selleck |
| CBP30 | HY15826 | MCE |
| CQ | NSC-187208 | Selleck |
| Sunitinib | S104211 | Selleck |
| Trehalose | C6138-23-47 | Sigma-Aldrich |
| MG132 | S2619 | Selleck |
| CHX | HY-12320 | MCE |
| Puromycin | S7417 | Selleck |

**Table S3.** The sequence of siRNAs for ERRα

| Name | Sequence |
| --- | --- |
| siRNA #1 sense | 5’-GAGAGGAGUAUGUUCUACUAATT-3’ |
| siRNA #1 antisense | 5’-UUAGUAGAACAUACUCCUCUCTT-3’ |
| siRNA #2 sense | 5’-GUGAAUGCACUGGUGUCUCAUTT-3’ |
| siRNA #2 antisense | 5’-AUGAGACACCAGUGCAUUCACTT-3’ |
| siRNA #3 sense | 5’-GAGGACUUAGUCCUGGAUGAATT-3’ |
| siRNA #3 antisense | 5’-UUCAUCCAGGACUAAGUCCUCTT-3’ |

**Table S4**. Primers used for RT-PCR

| Name | Sequence |
| --- | --- |
| RAB7A sense | 5’-GTGTTGCTGAAGGTTATCATCCT-3’ |
| RAB7A antisense | 5’-GCTCCTATTGTGGCTTTGTACTG-3’ |
| KIF5B sense | 5’-TGTGACTCCTAGAGCTATGTTCA-3’ |
| KIF5B antisense | 5’-AGACAAAAACCGAGTTCCCTATG-3’ |
| CTSB sense | 5’-ACAACGTGGACATGAGCTACT-3’ |
| CTSB antisense | 5’-TCGGTAAACATAACTCTCTGGGG-3’ |
| GBA sense | 5’-ATGGAGCGGTGAATGGGAAG-3’ |
| GBA antisense | 5’-GTGCTCAGCATAGGCATCCAG-3’ |
| VAMP8 sense | 5’-TGTGCGGAACCTGCAAAGT-3’ |
| VAMP8 antisense | 5’-CTTCTGCGATGTCGTCTTGAA-3’ |
| CTSC sense | 5’-CCAACTGCACCTATCTTGACC-3’ |
| CTSC antisense | 5’-AAGGCAAACCACTTGTAGTCATT-3’ |
| CTSD sense | 5’-TGCTCAAGAACTACATGGACGC-3’ |
| CTSD antisense | 5’-CGAAGACGACTGTGAAGCACT-3’ |
| TPP1 sense | 5’-GTTTCATCACTATGTGGGAGGAC-3’ |
| TPP1 antisense | 5’-GTATCGCTTACGGATCACAGAG-3’ |
| PSAP sense | 5’-GGGAGGTAGGAGTCCACTATCT-3’ |
| PSAP antisense | 5’-ATGCAAAGACGTTGTCACCG-3’ |

**Table 5.** Primers used for ChIP

| Name | Sequence |
| --- | --- |
| VAMP8-1 sense | 5’-AACCAAATTGCTCCCACCCATAAAC-3’ |
| VAMP8-1 antisense | 5’-GCAGGACAGCCGAATAGGGAAA-3’ |
| VAMP8-2 sense | 5’-CAGTGCAGCCCCCTCTCCACGAT-3’ |
| VAMP8-2 antisense | 5’-TCTGTGGGGAAGGGGGCGTGC-3’ |
| LAMP2 sense | 5’-GACACAGGGAGGGGAACAACACACA-3’ |
| LAMP2 antisense | 5’-ACTGCACCTATCAACCCGTCATCTA-3’ |
