# Supplementary material for: Hypoxia-triggered ERRα acetylation enhanced its oncogenic role and promoted progression of renal cell carcinoma by coordinating autophagosome-lysosome fusion
Source: Cell Death Dis. 2025 Jan 16;16(1):23. doi: 10.1038/s41419-025-07345-1 (PMC11739407; doi:10.1038/s41419-025-07345-1)
Supplement: Supplementary file 5 — Original images of WB [file 41419_2025_7345_MOESM5_ESM.pdf]

Figure1A

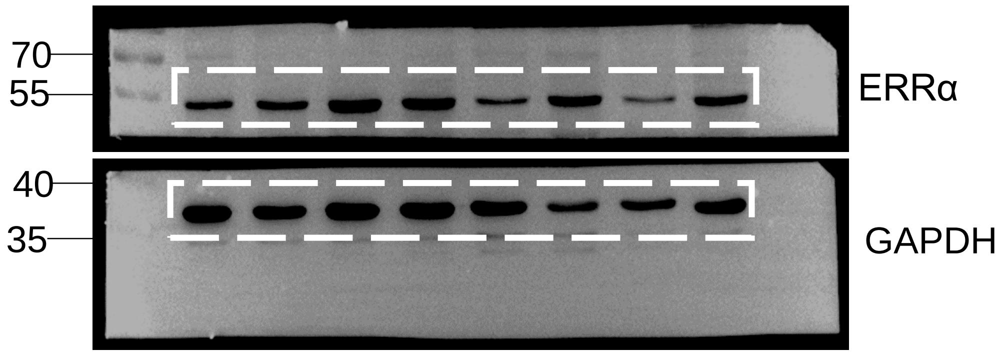

Figure1B

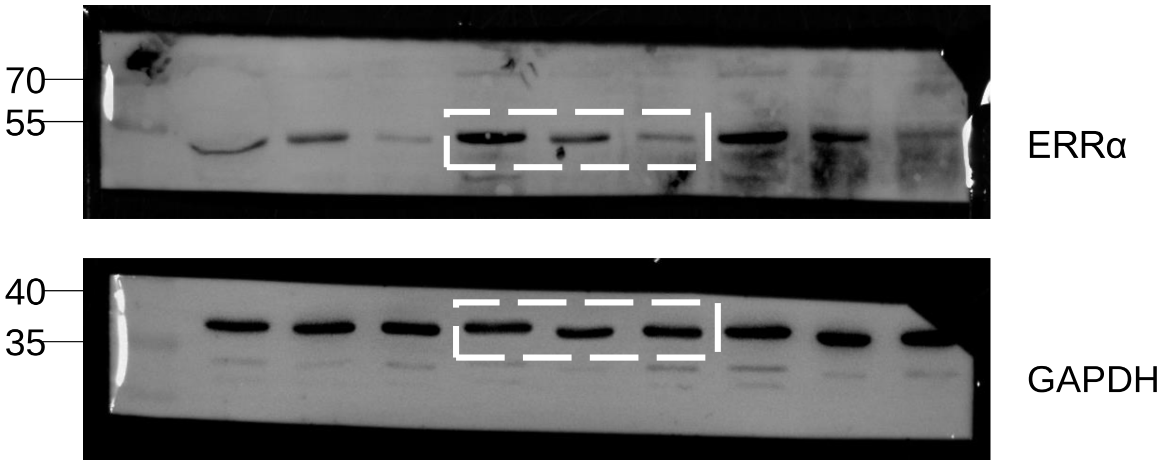

Figure1E

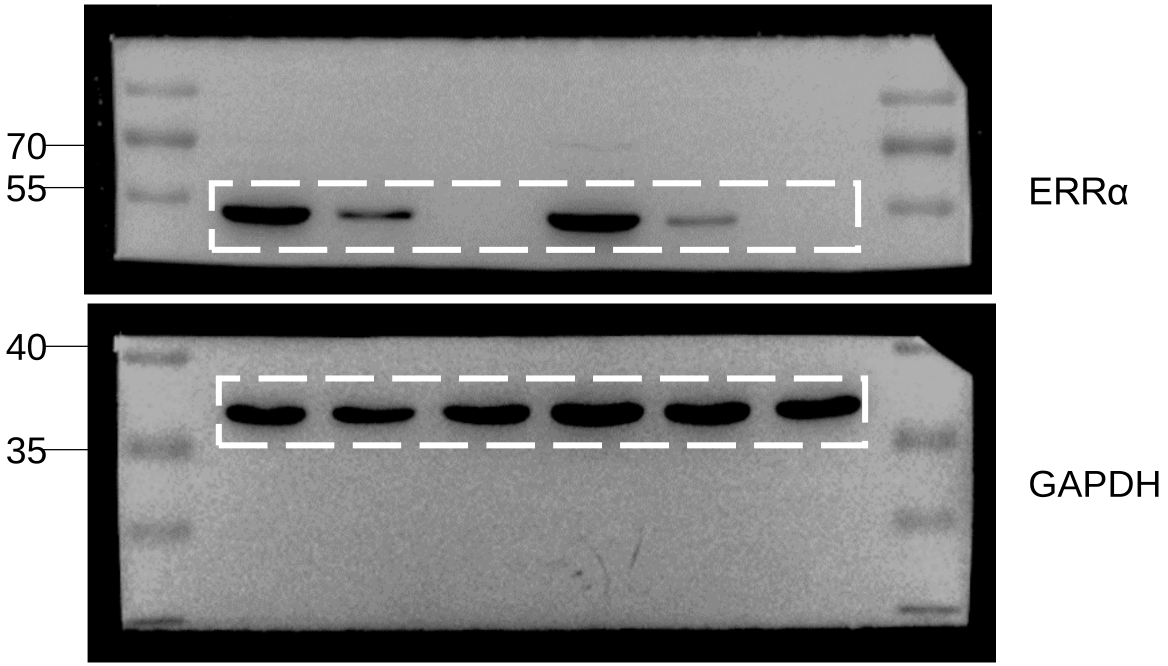

Figure2E

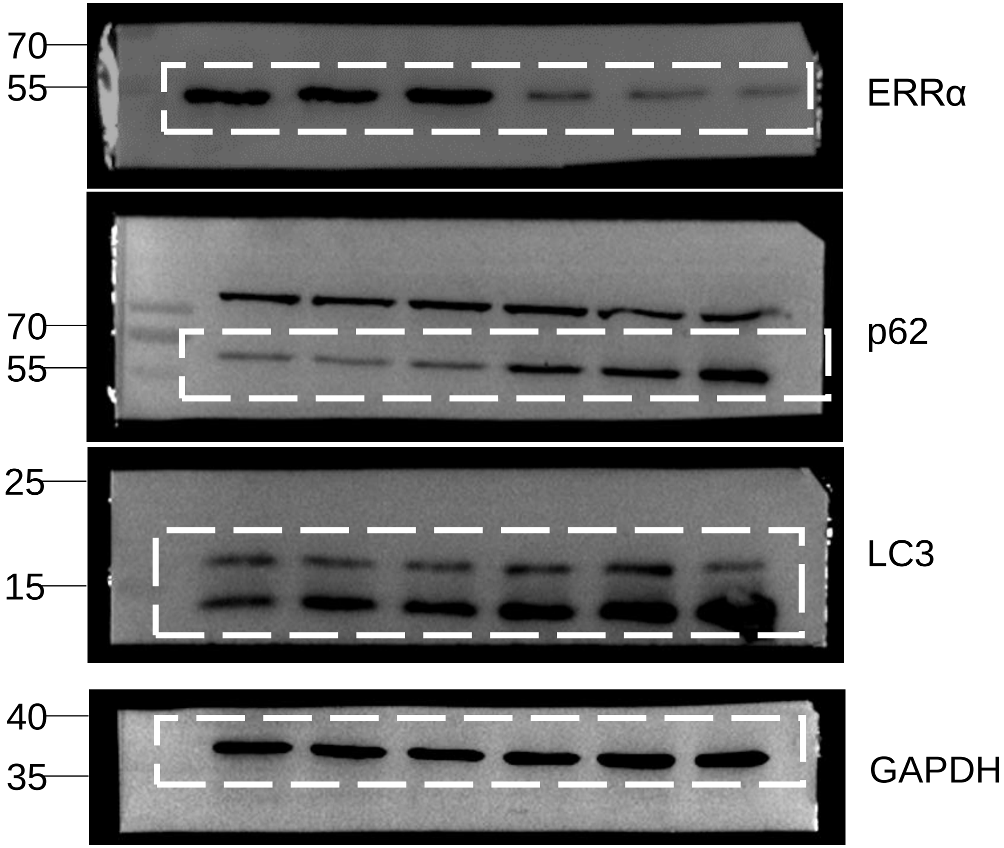

Figure2H

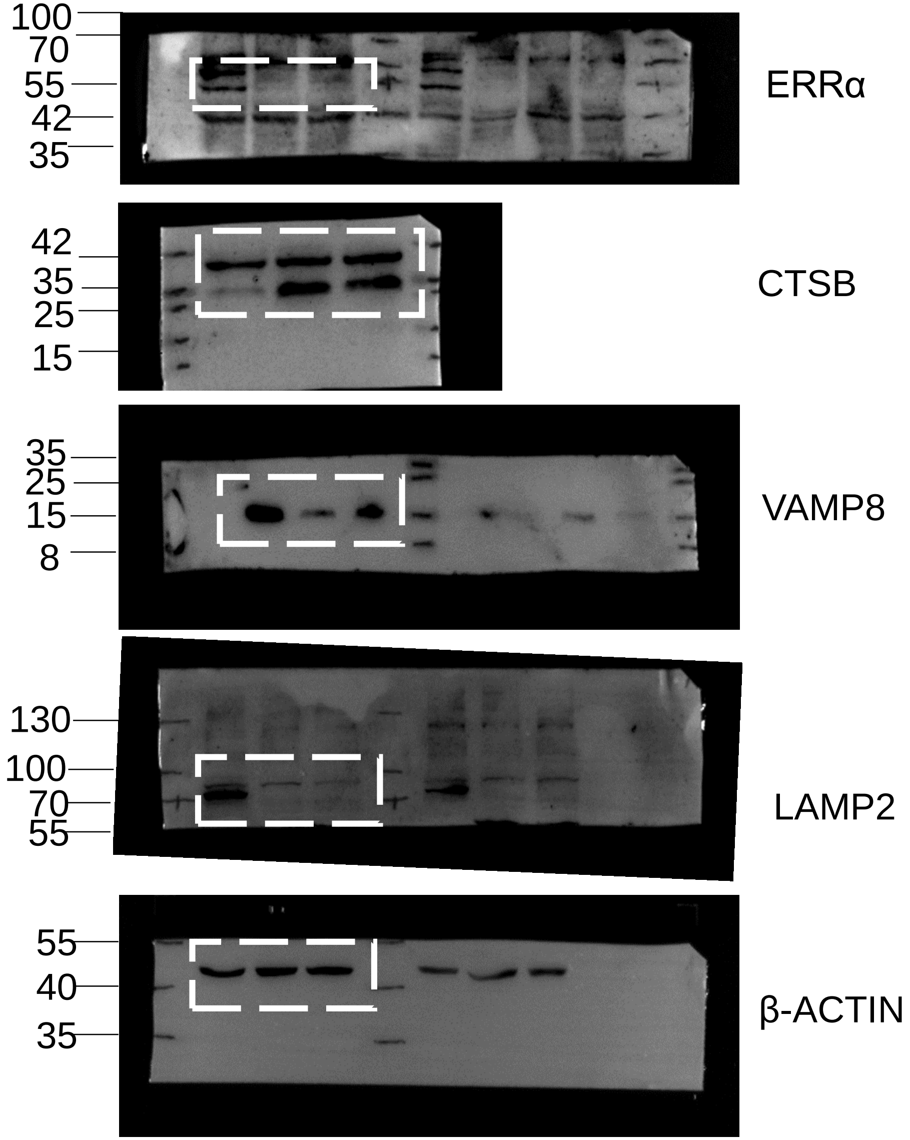

FigureS2A CQ

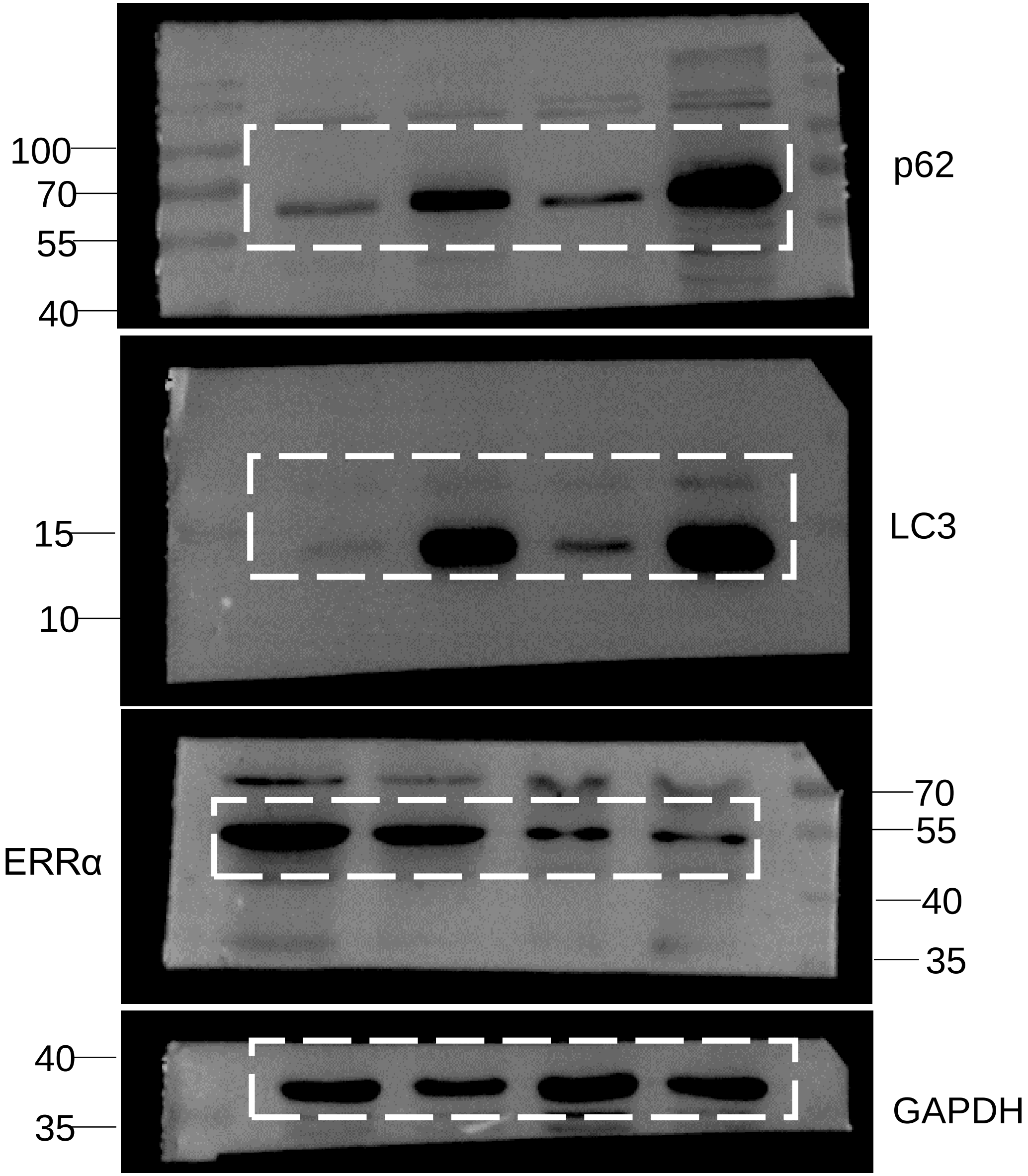

FigureS2A Baf-A1

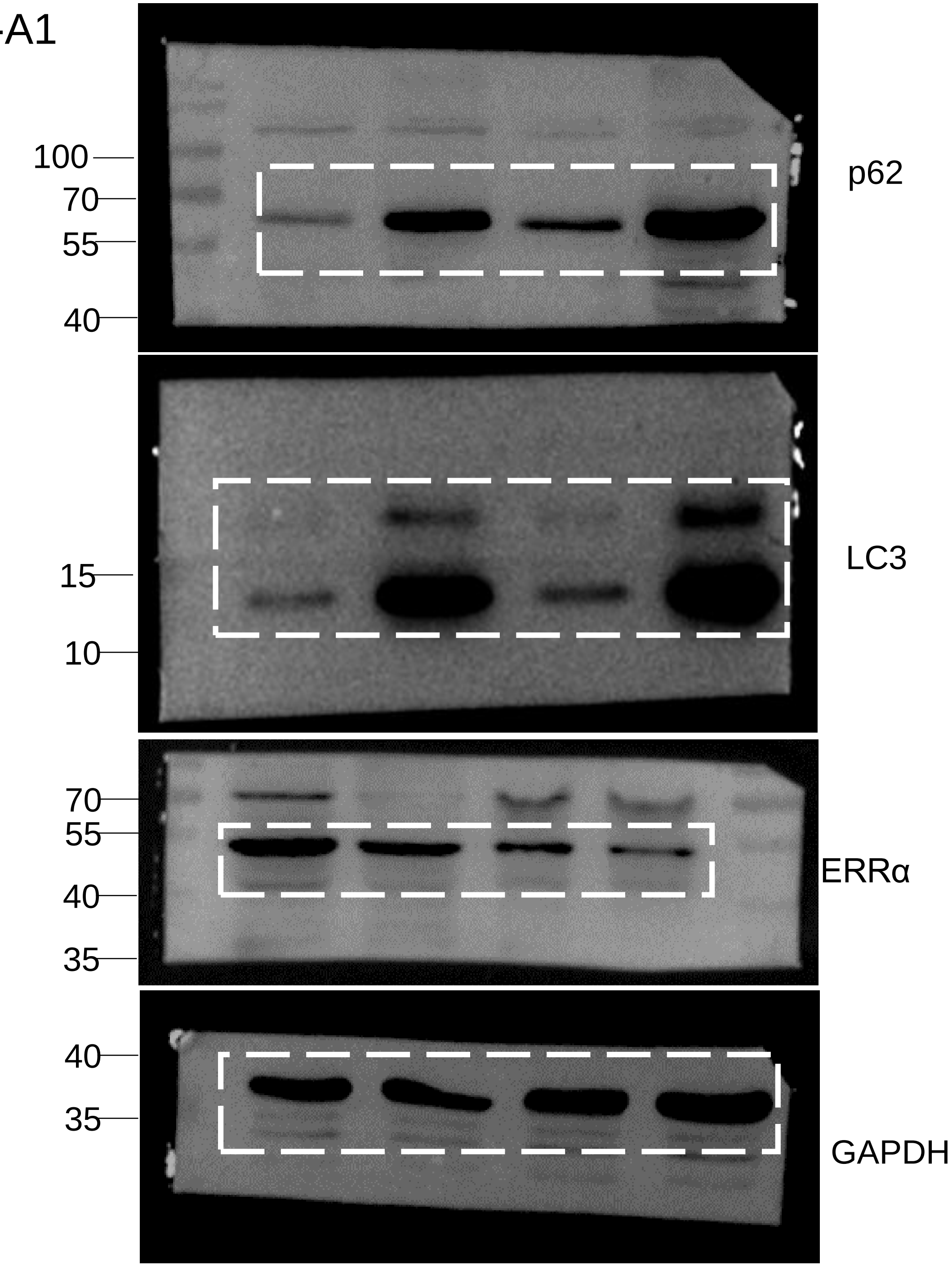

Figure4A

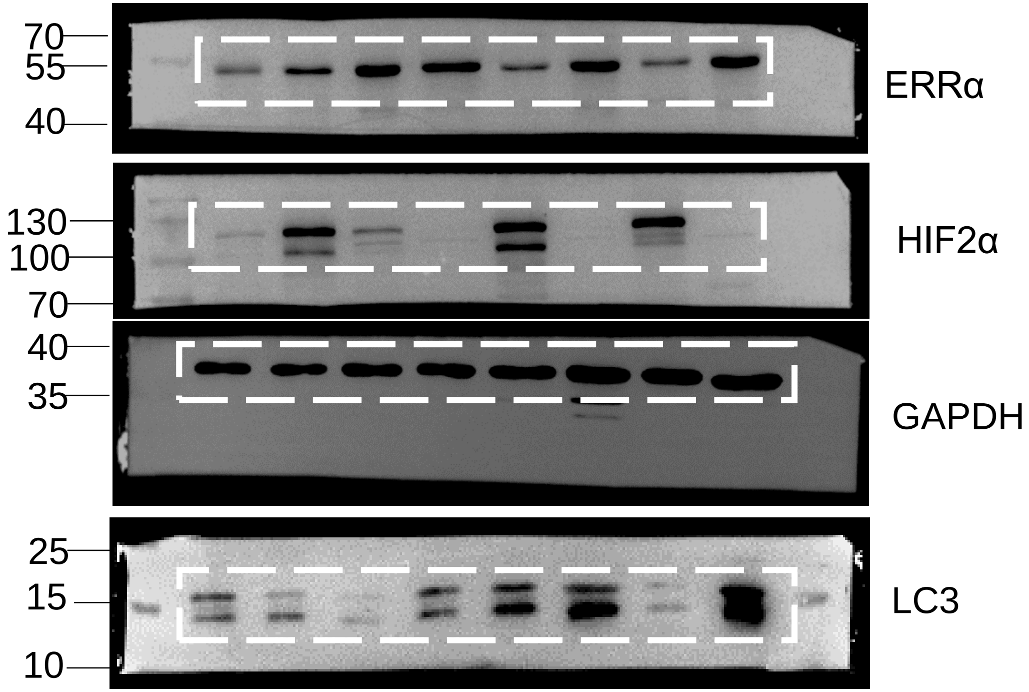

Figure4B

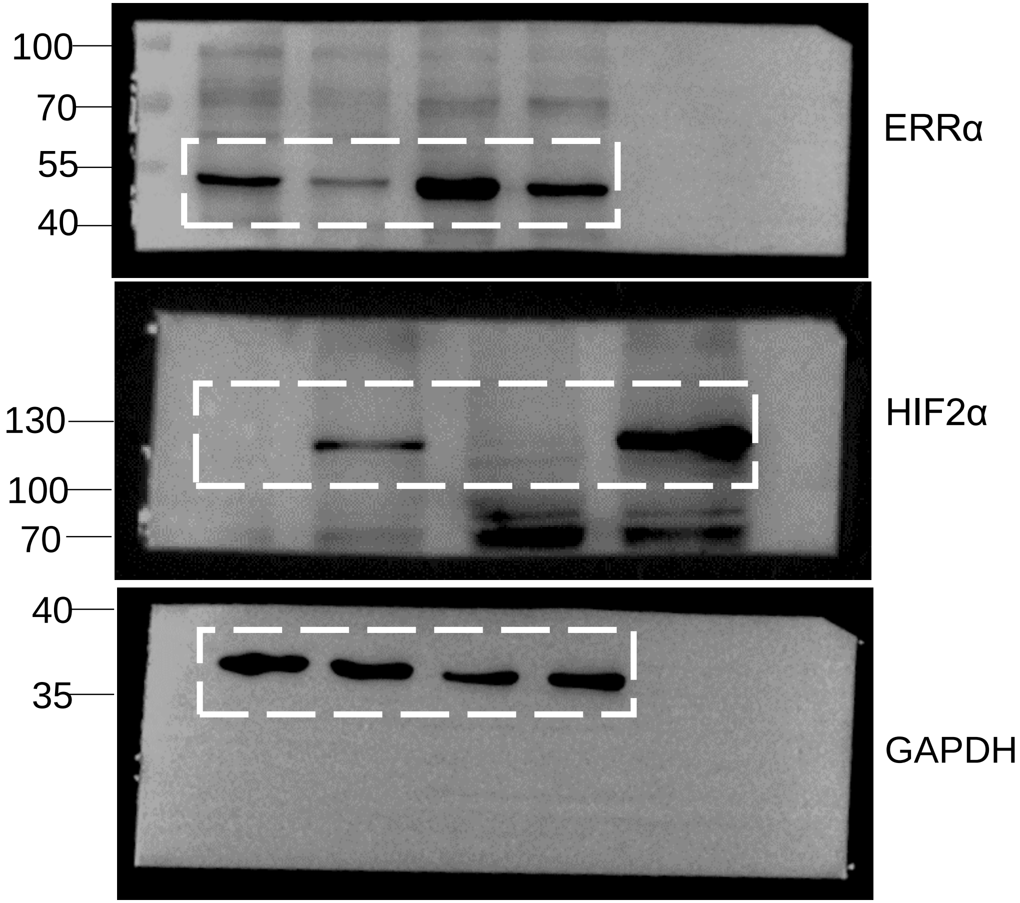

Figure4C

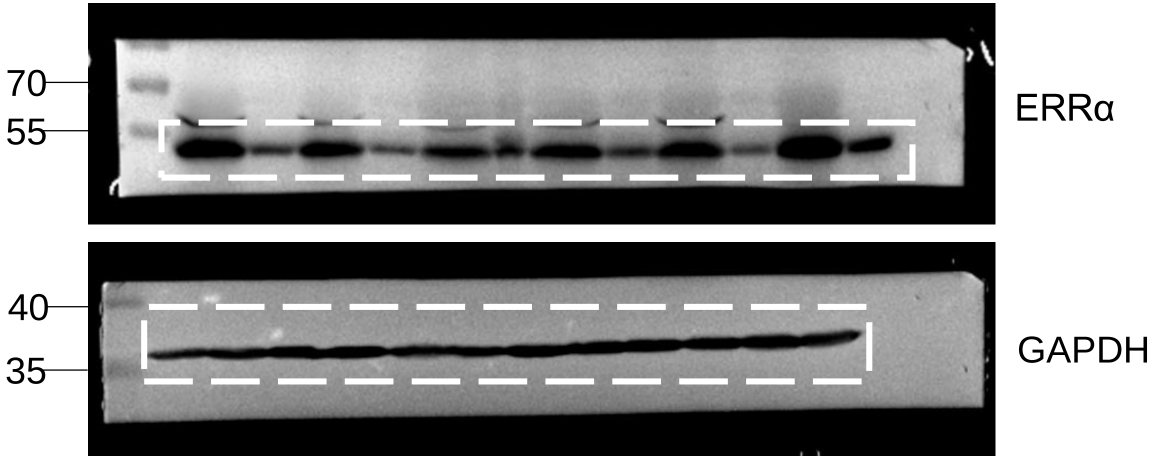

Figure4E

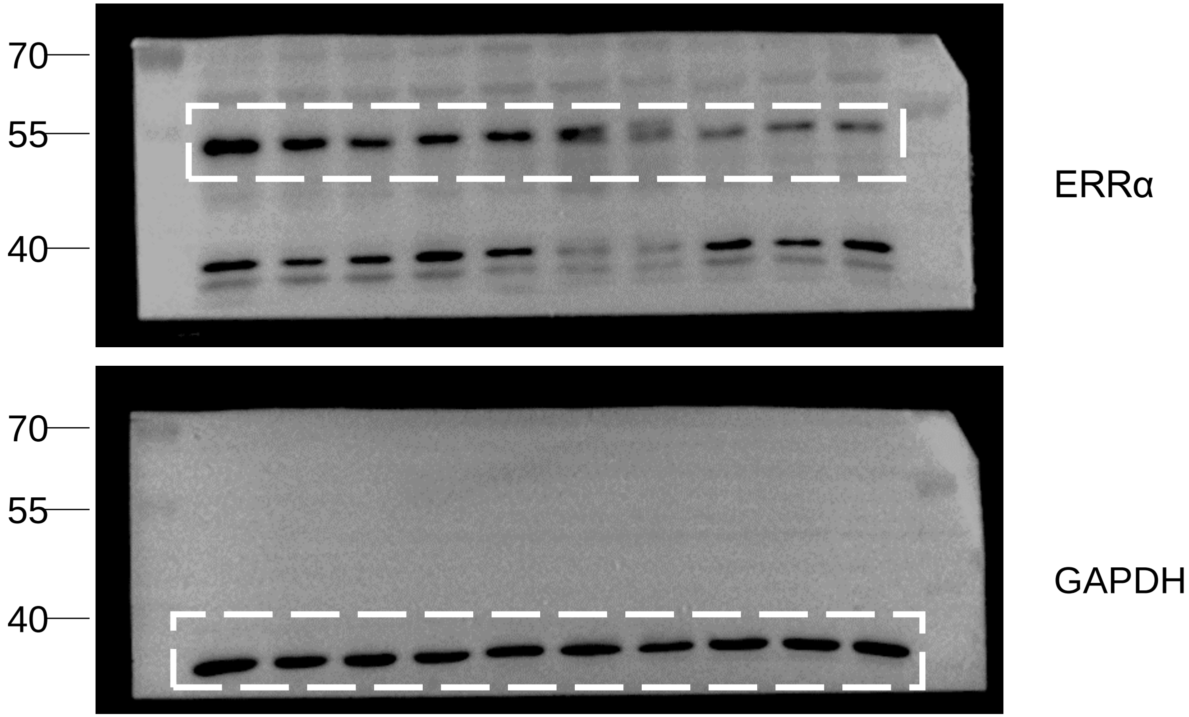

Figure4F

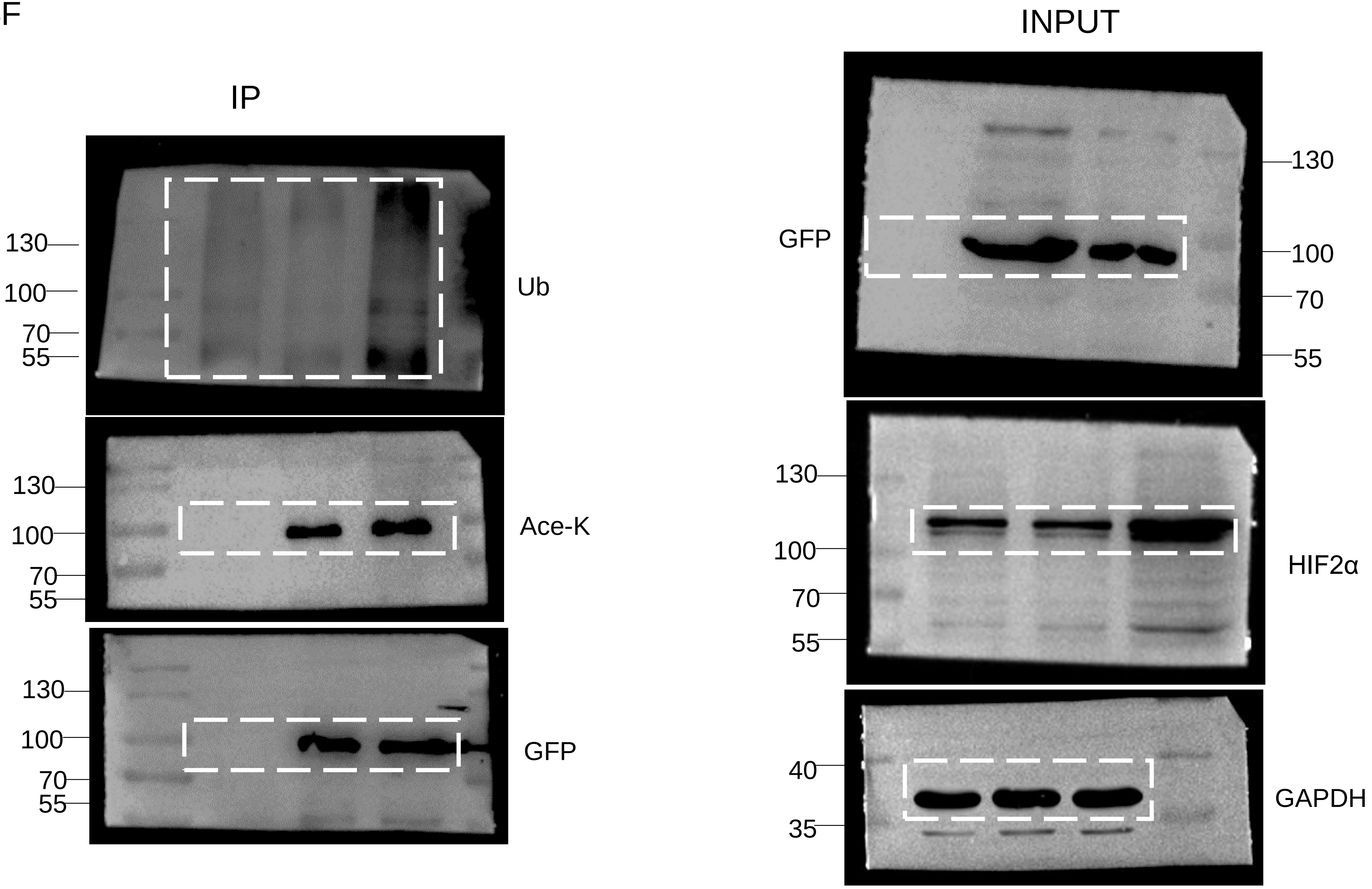
IP
130
100
70
55
Ub
130
100
70
55
Ace-K
130
100
70
55
GFP
INPUT
GFP
130
100
70
55
130
100
70
55
HIF2α
40
35
GAPDH

Figure 4H

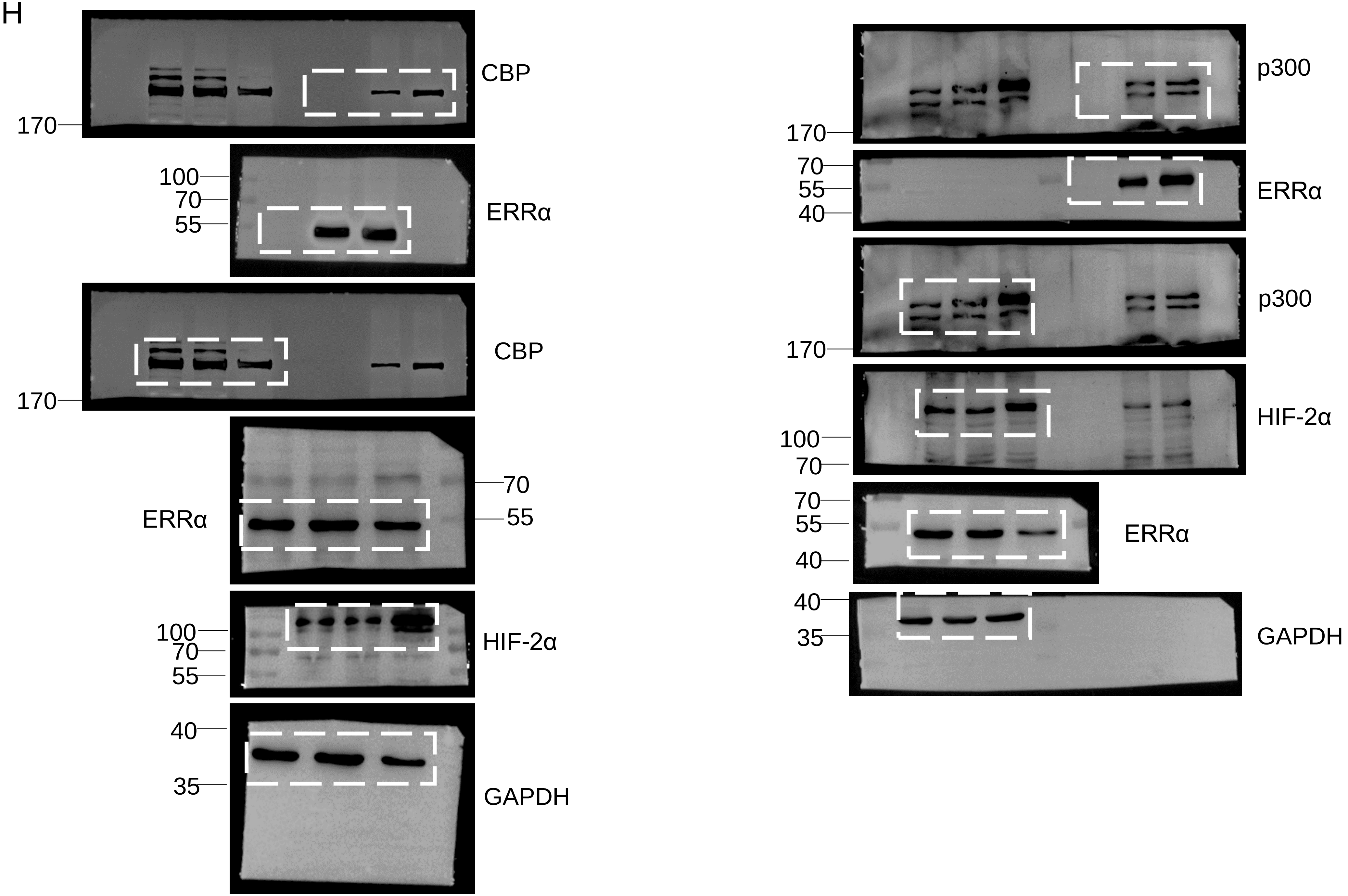

CBP
170
100
70
55
ERRα
CBP
170
ERRα
70
55
100
70
55
HIF-2α
40
35
GAPDH
p300
170
70
55
40
ERRα
p300
170
HIF-2α
100
70
70
55
40
ERRα
40
35
GAPDH

Figure4I

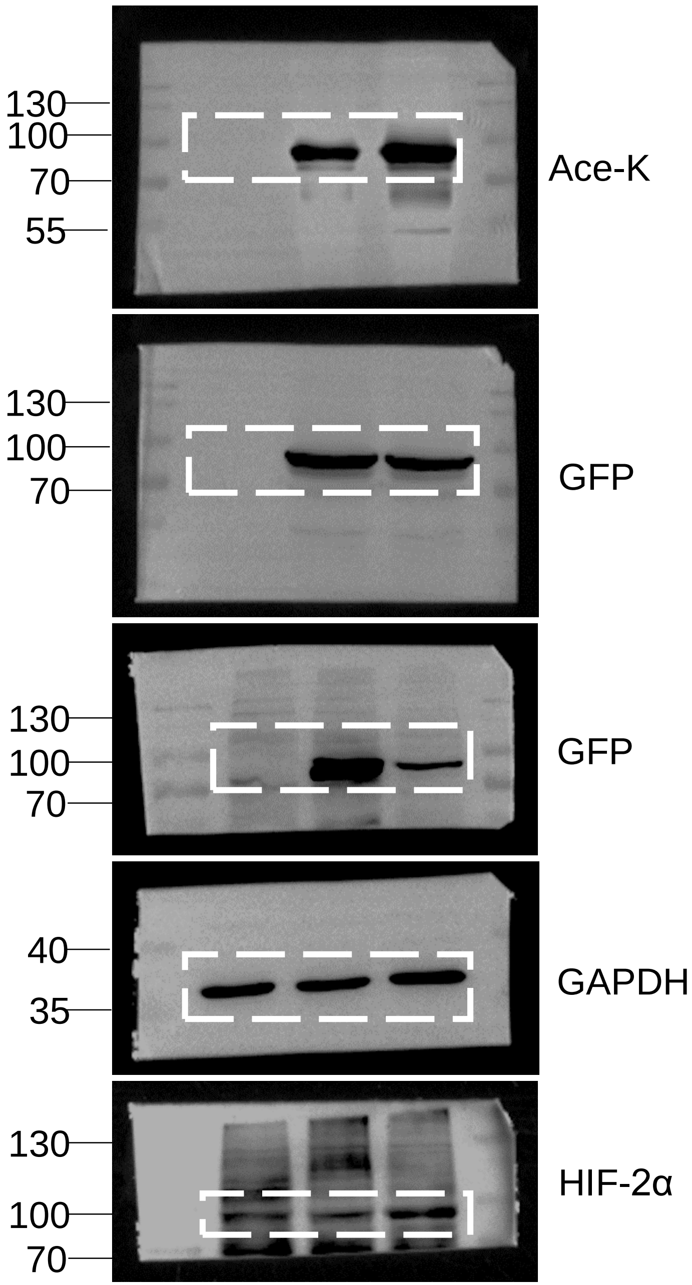

Figure4J

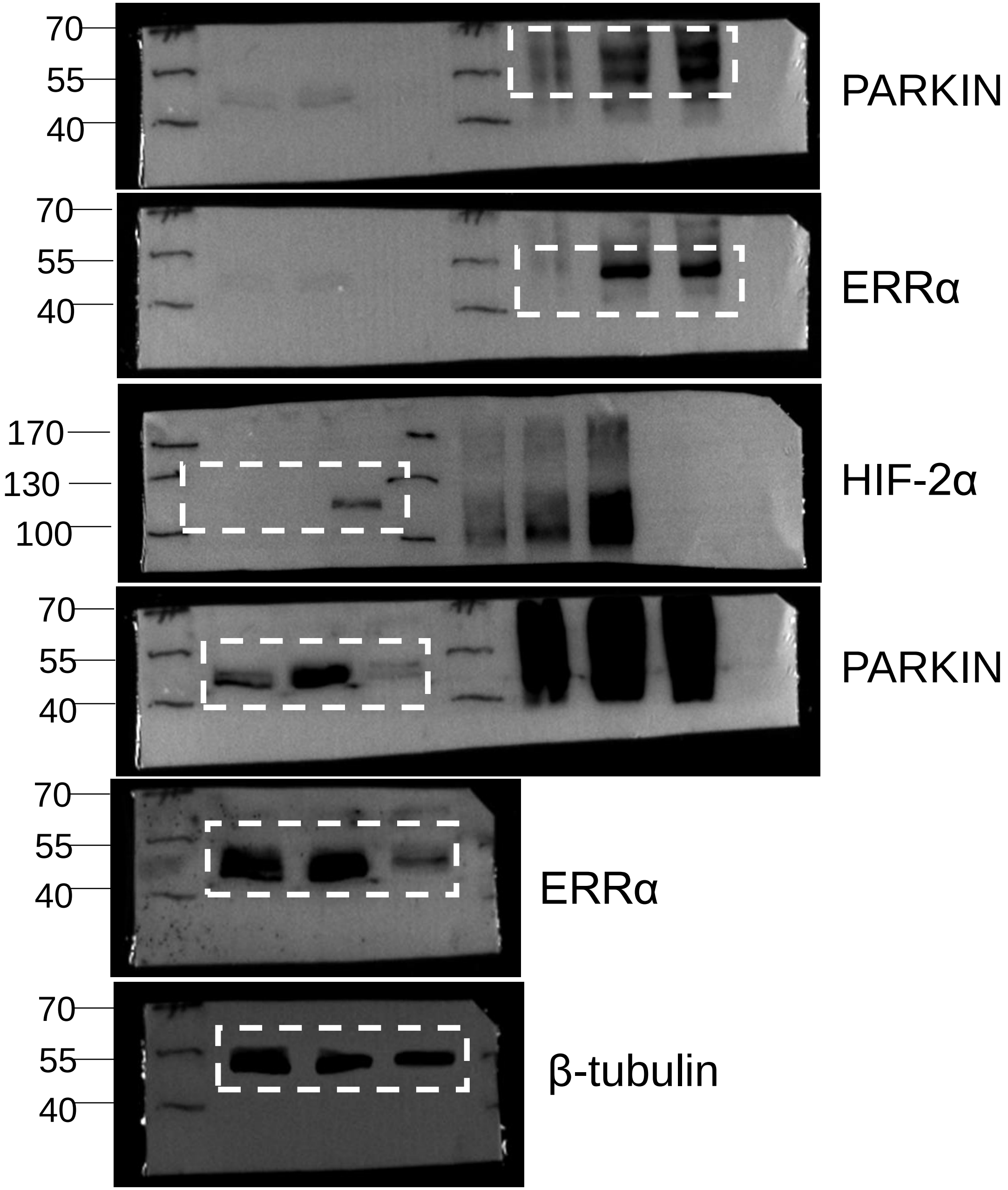

Figure5A

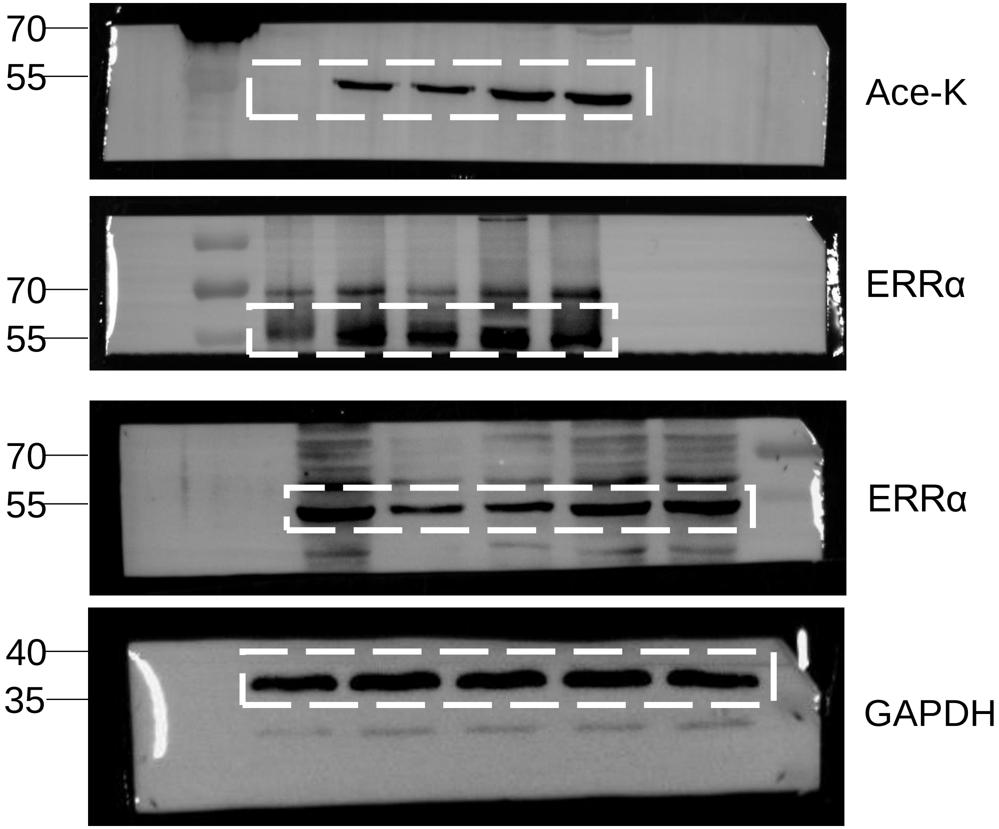

Figure5B

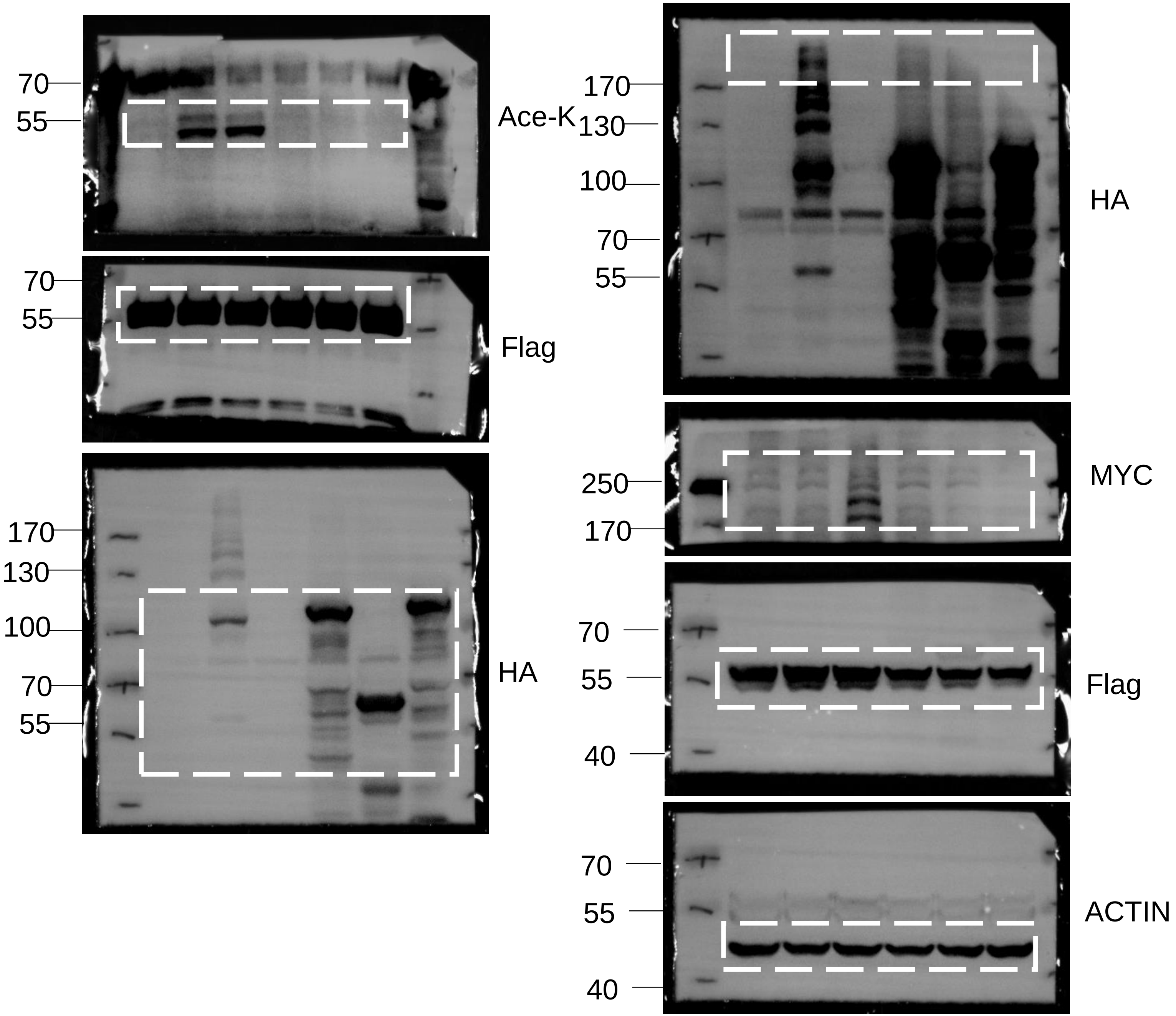
70
55
Ace-K
70
55
Flag
170
130
100
70
55
HA
170
130
100
70
55
HA
250
170
MYC
70
55
40
Flag
70
55
40
ACTIN

Figure5C

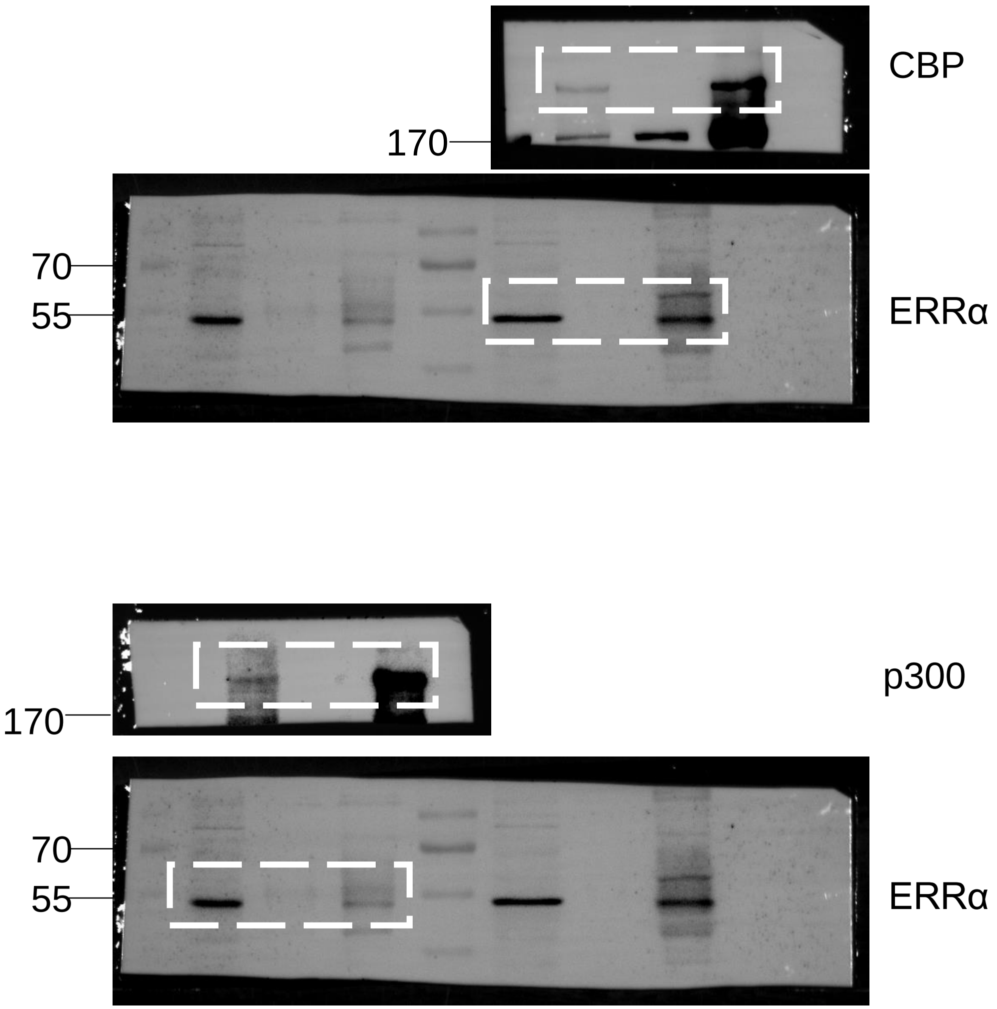

Figure5D

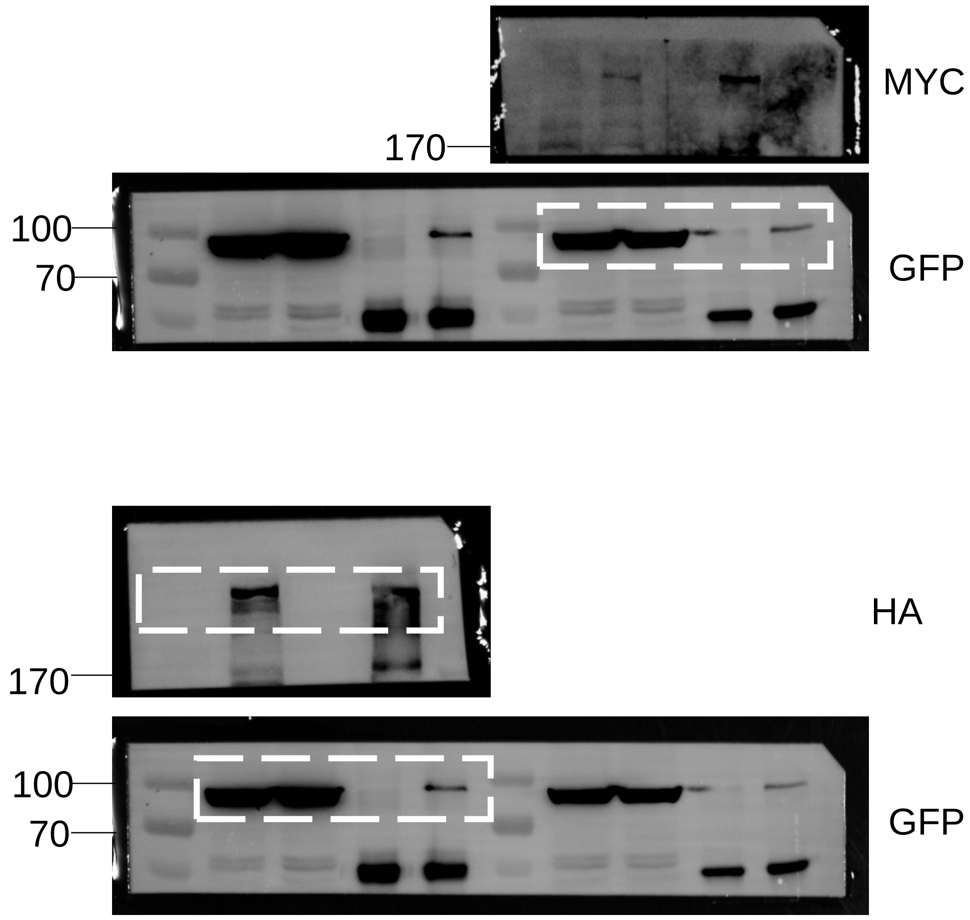

Figure5E

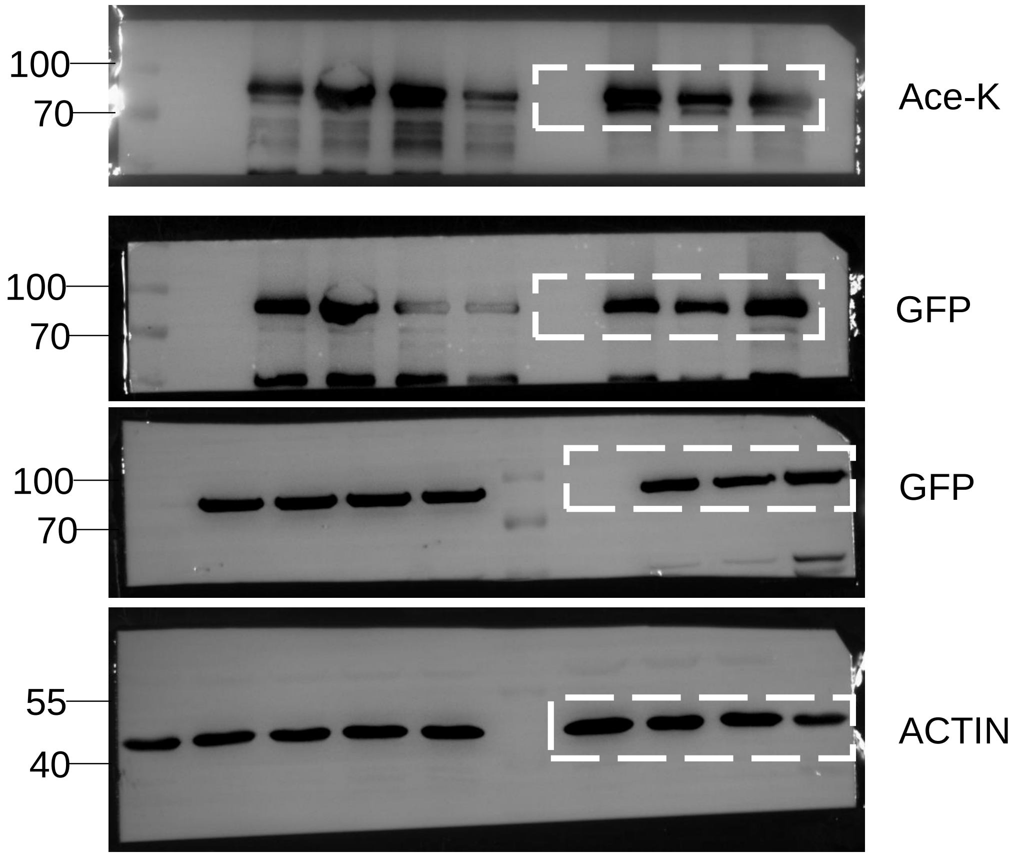

Figure5G

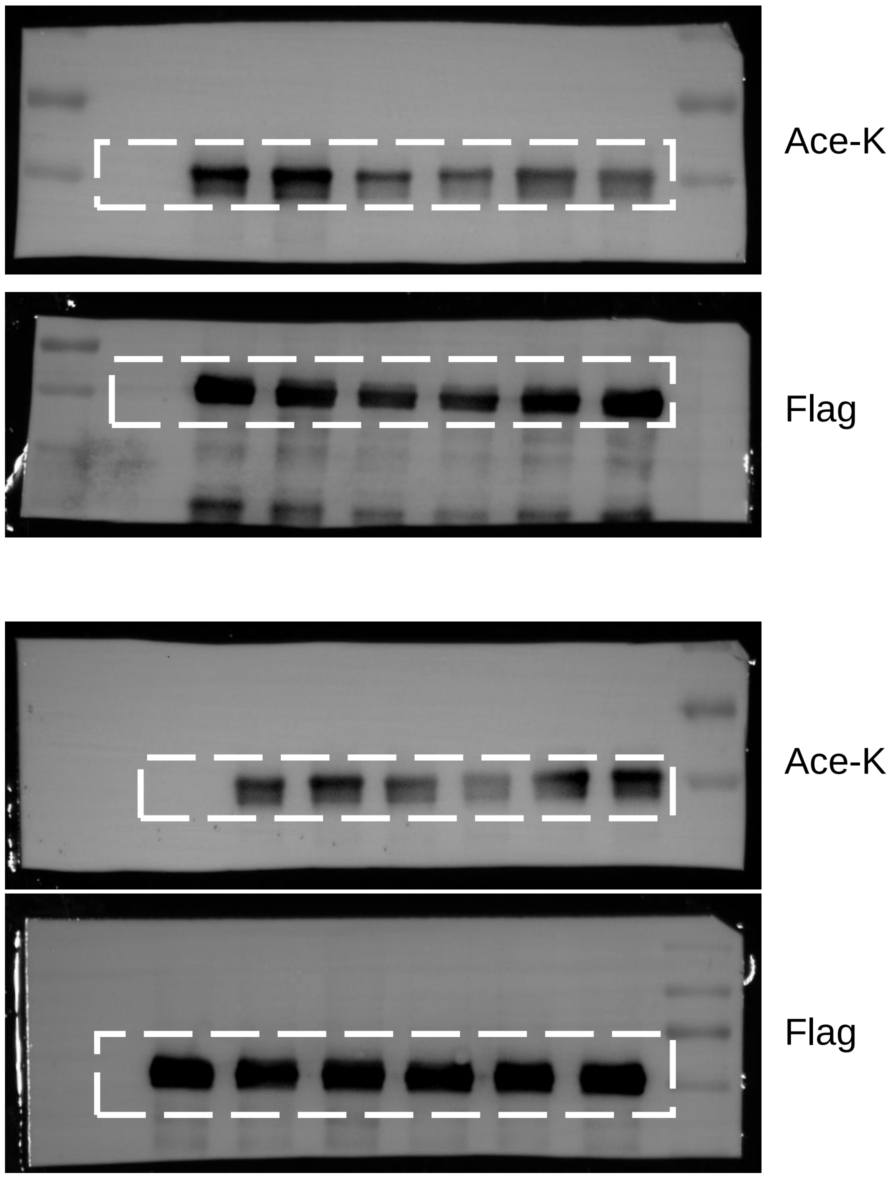

Figure5H

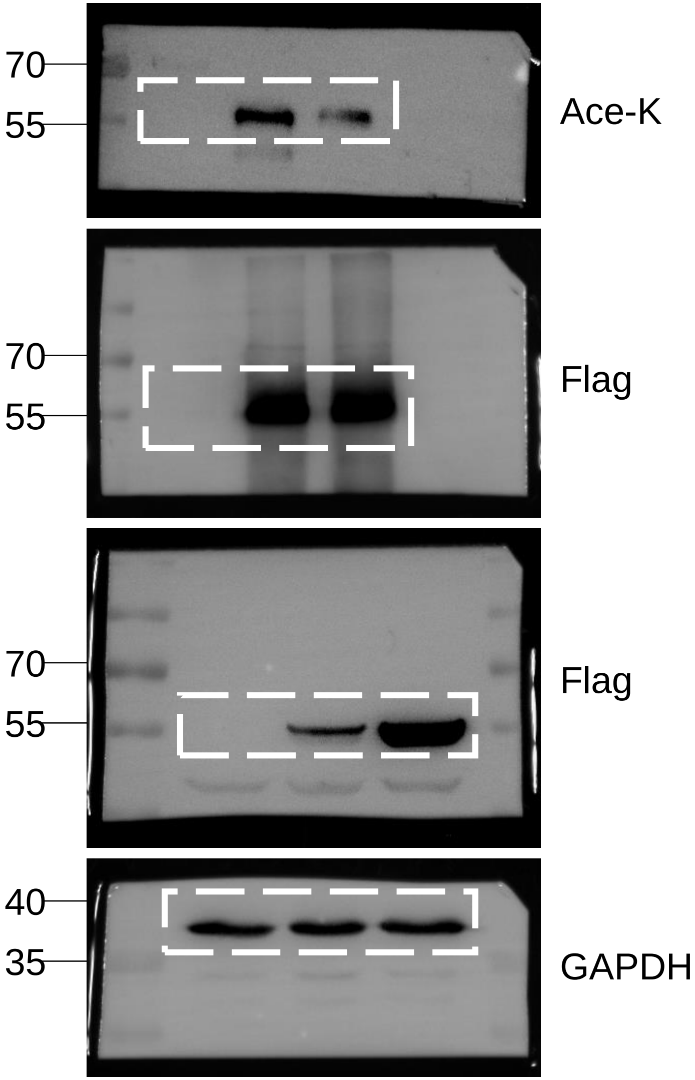

Figure S3B

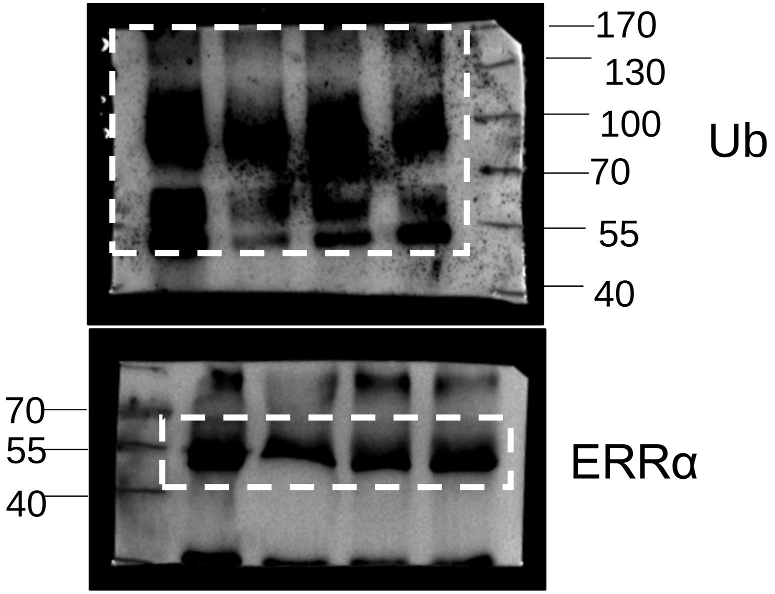
170
130
100
70
55
40
Ub
70
55
40
ERRα

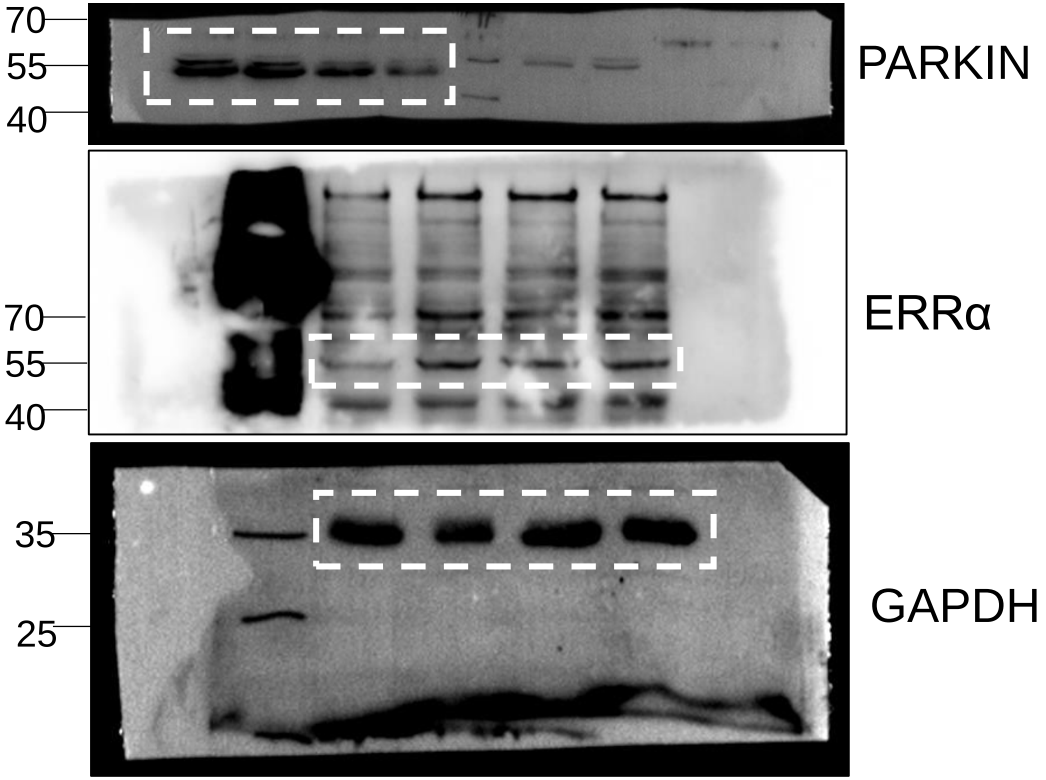
70
55
40
PARKIN
70
55
40
ERRα
35
25
GAPDH

Figure S3E

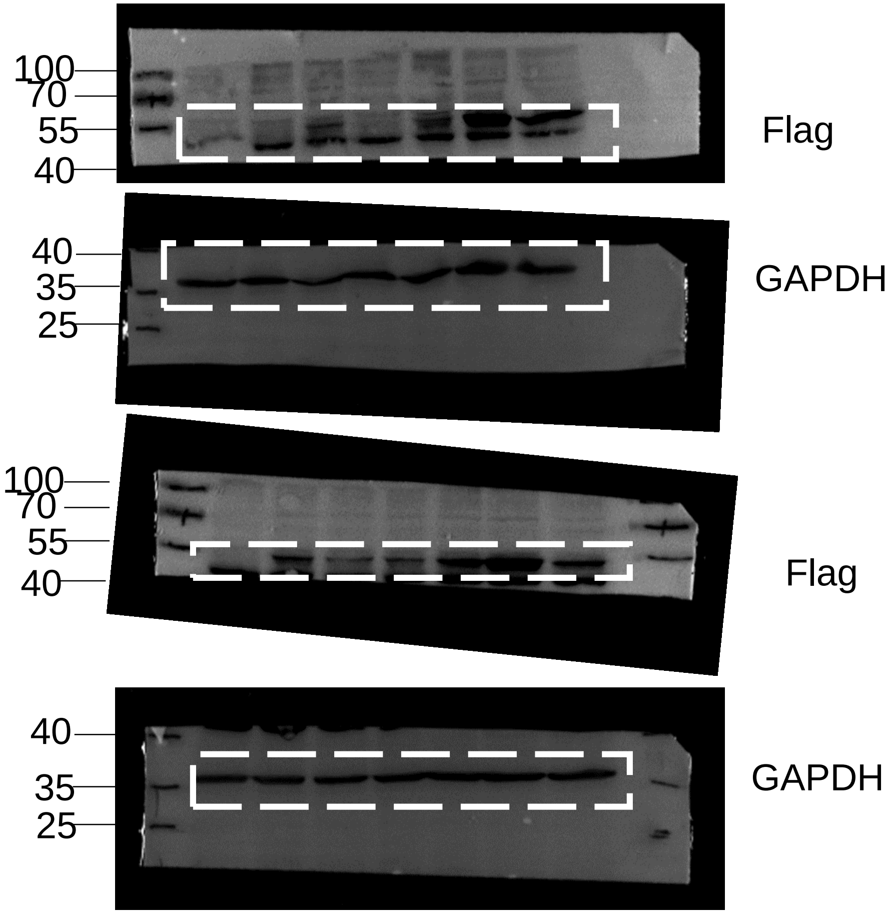

Figure6A

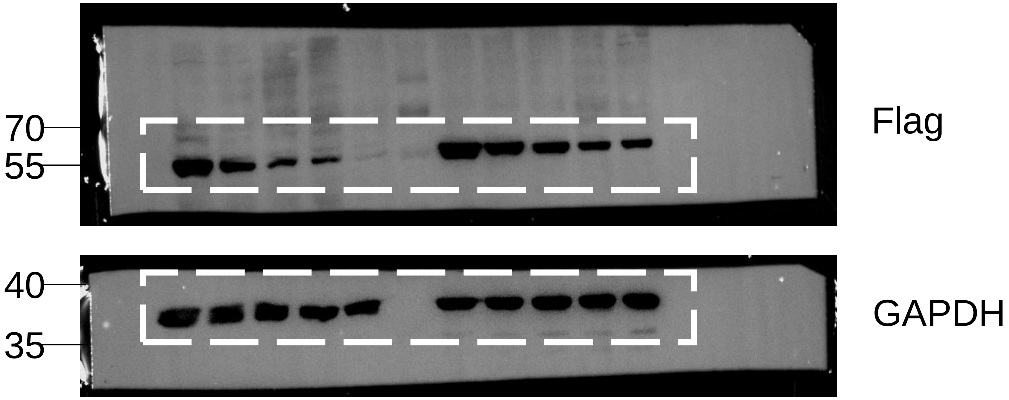

Figure6B

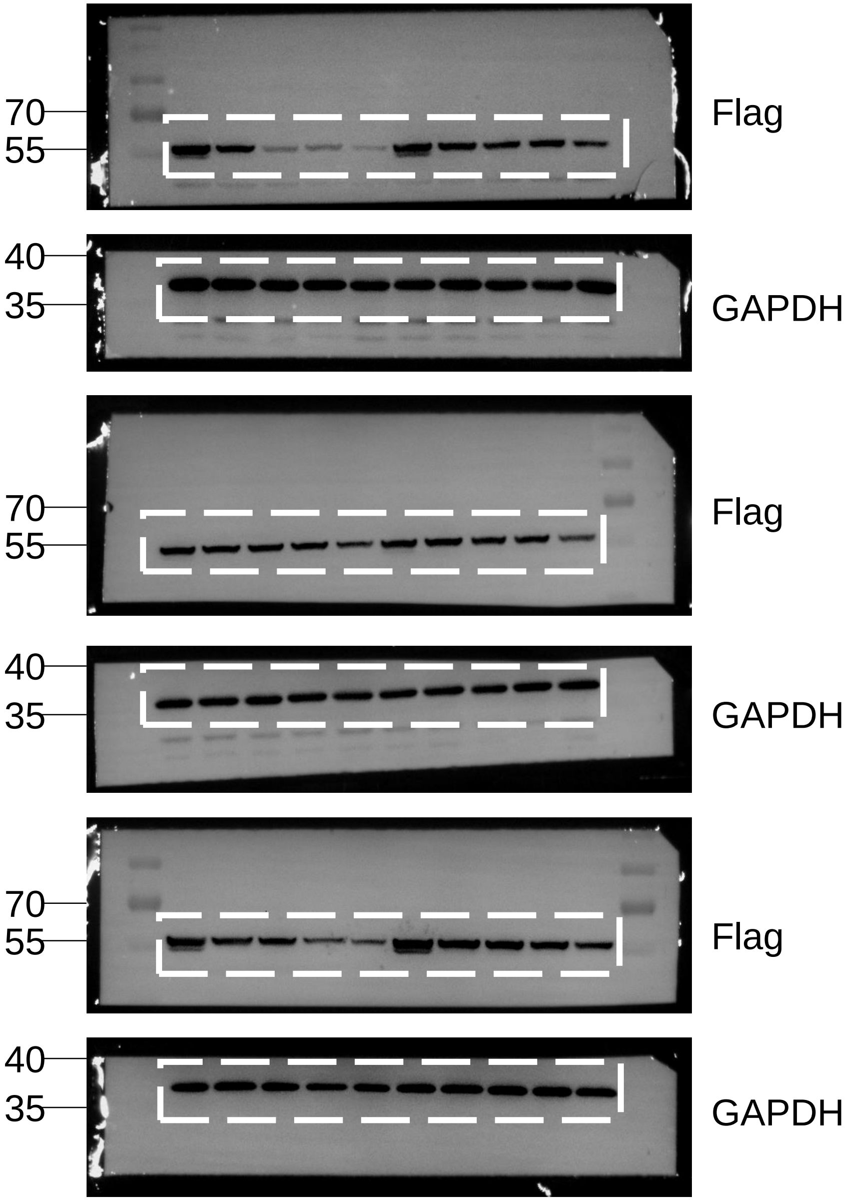

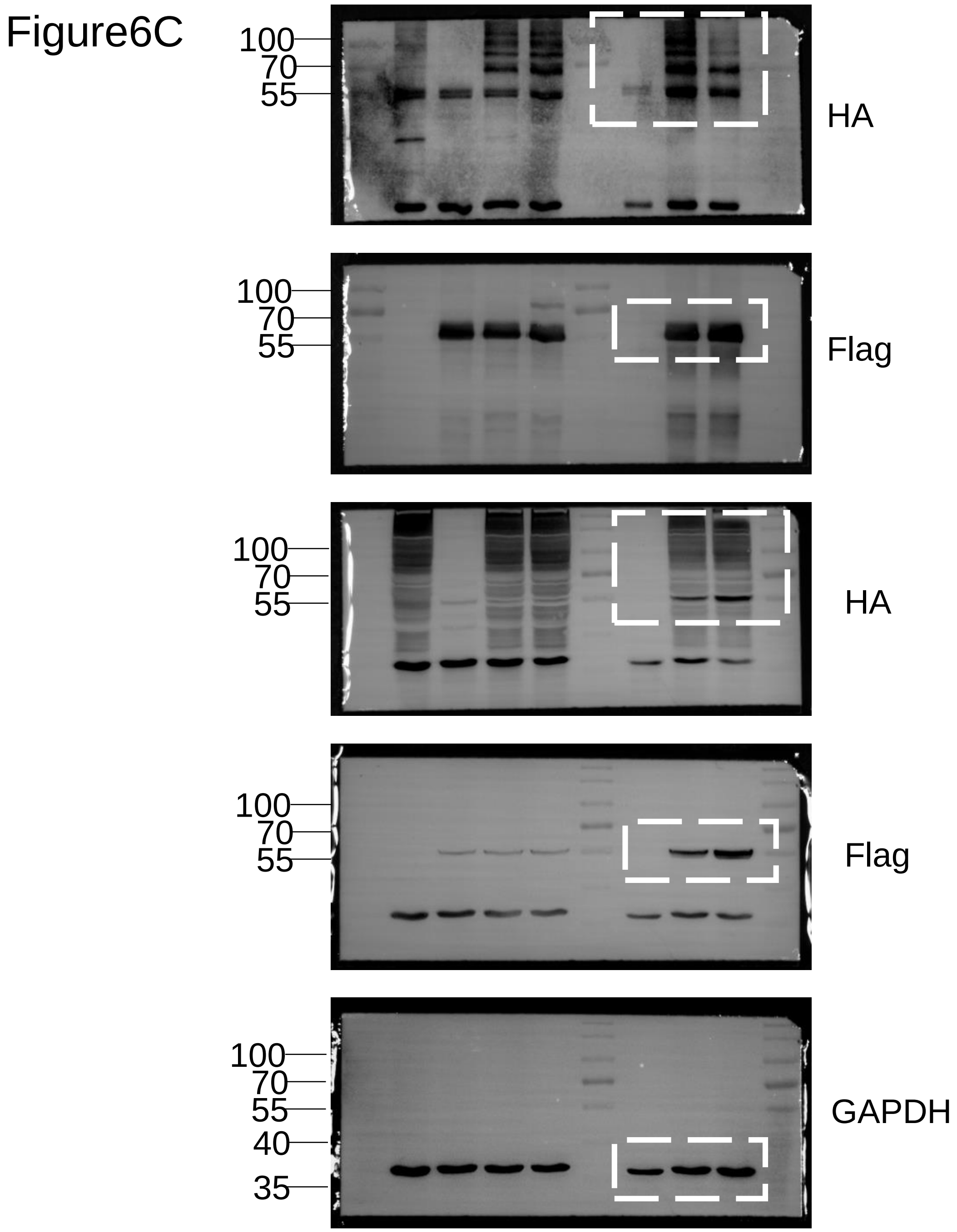
Figure6C
100
70
55
HA
100
70
55
Flag
100
70
55
HA
100
70
55
Flag
100
70
55
40
35
GAPDH

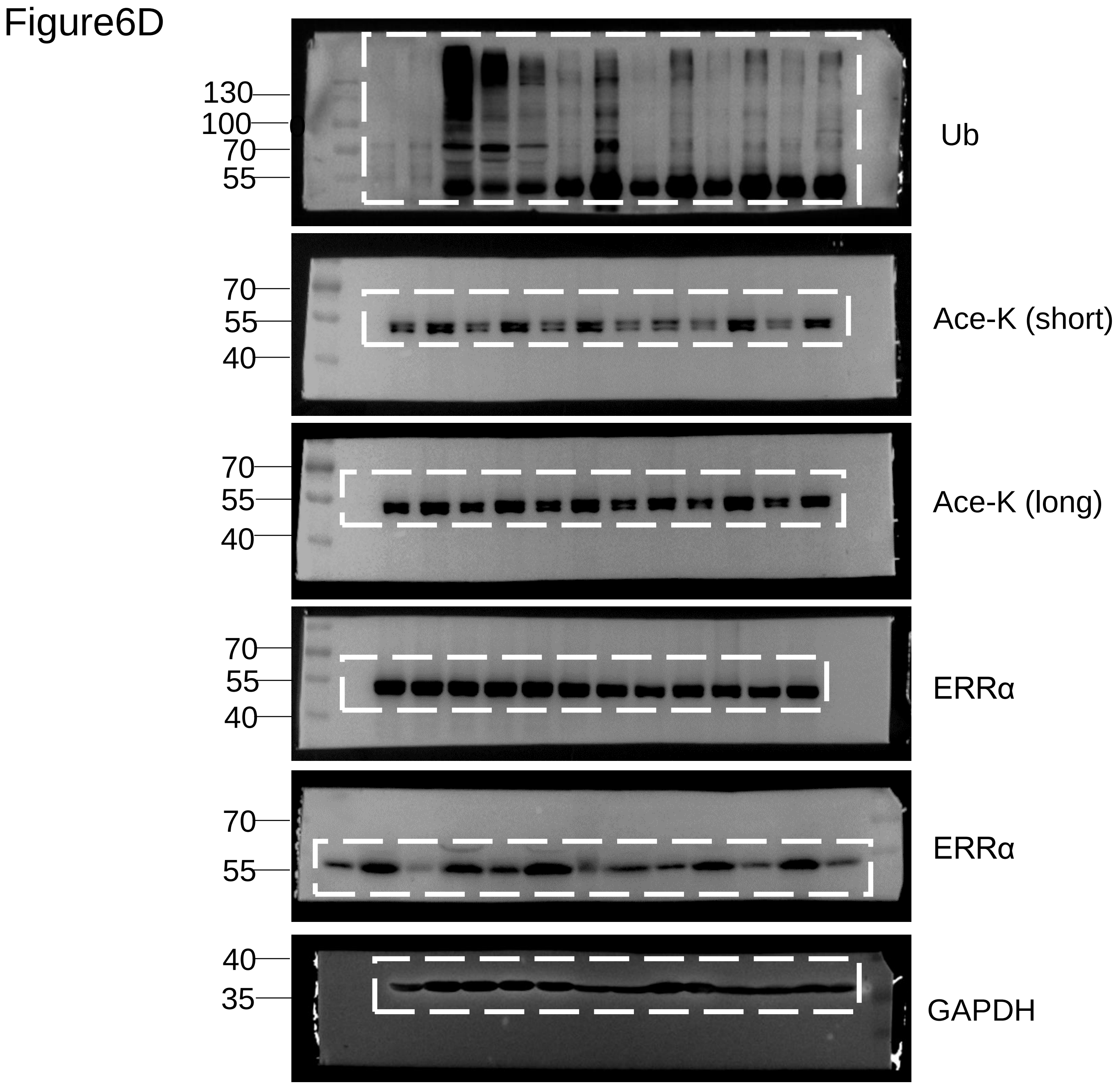
Figure6D
130
100
70
55
Ub
70
55
40
Ace-K (short)
70
55
40
Ace-K (long)
70
55
40
ERRα
70
55
ERRα
40
35
GAPDH

Figure6H

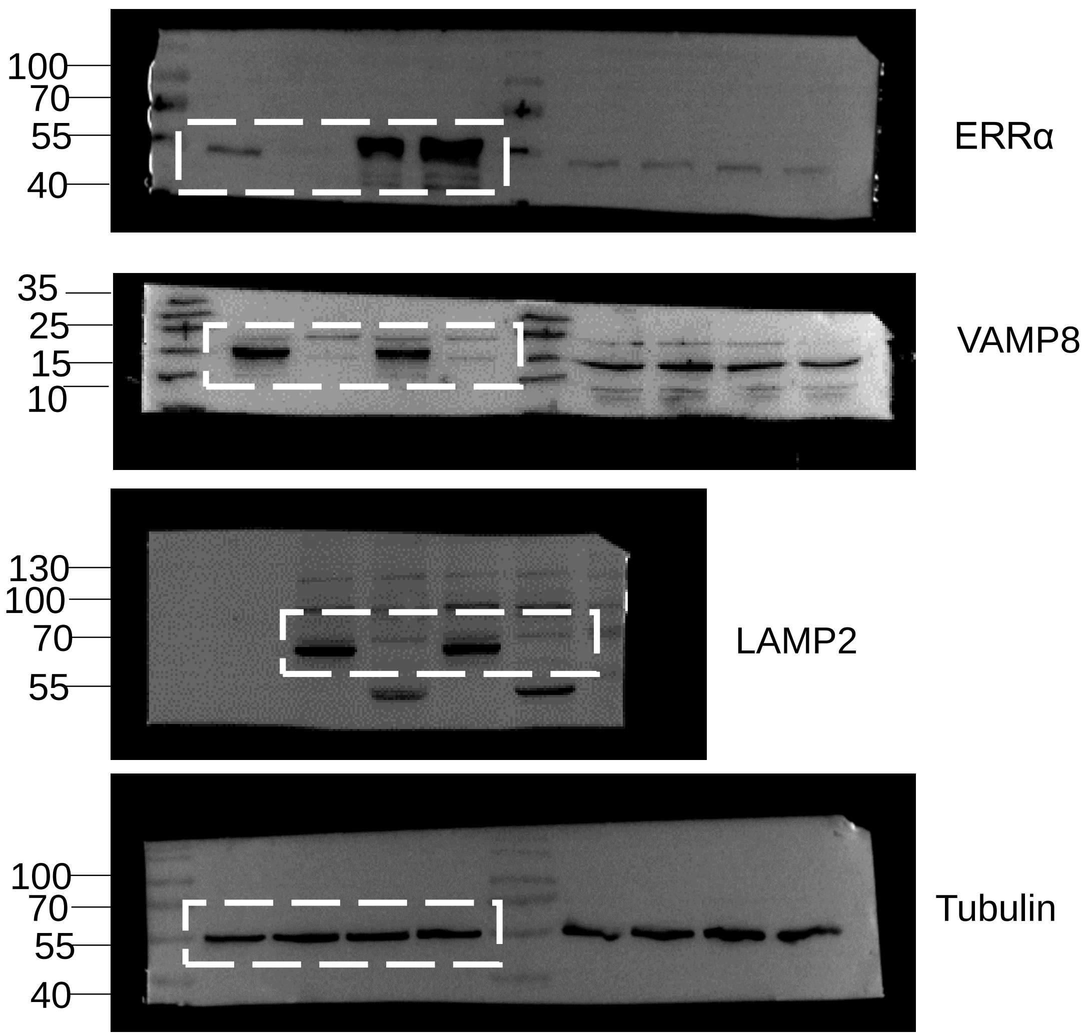

Figure 6I

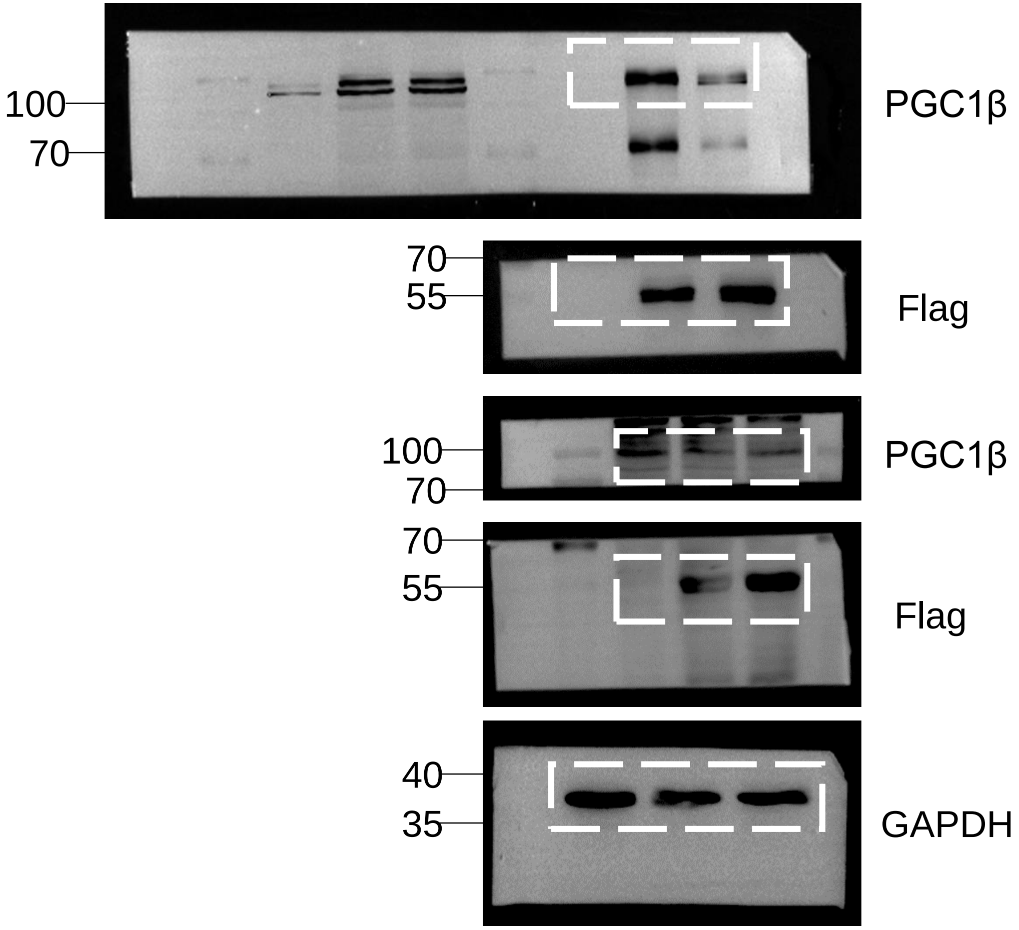

Figure S4A

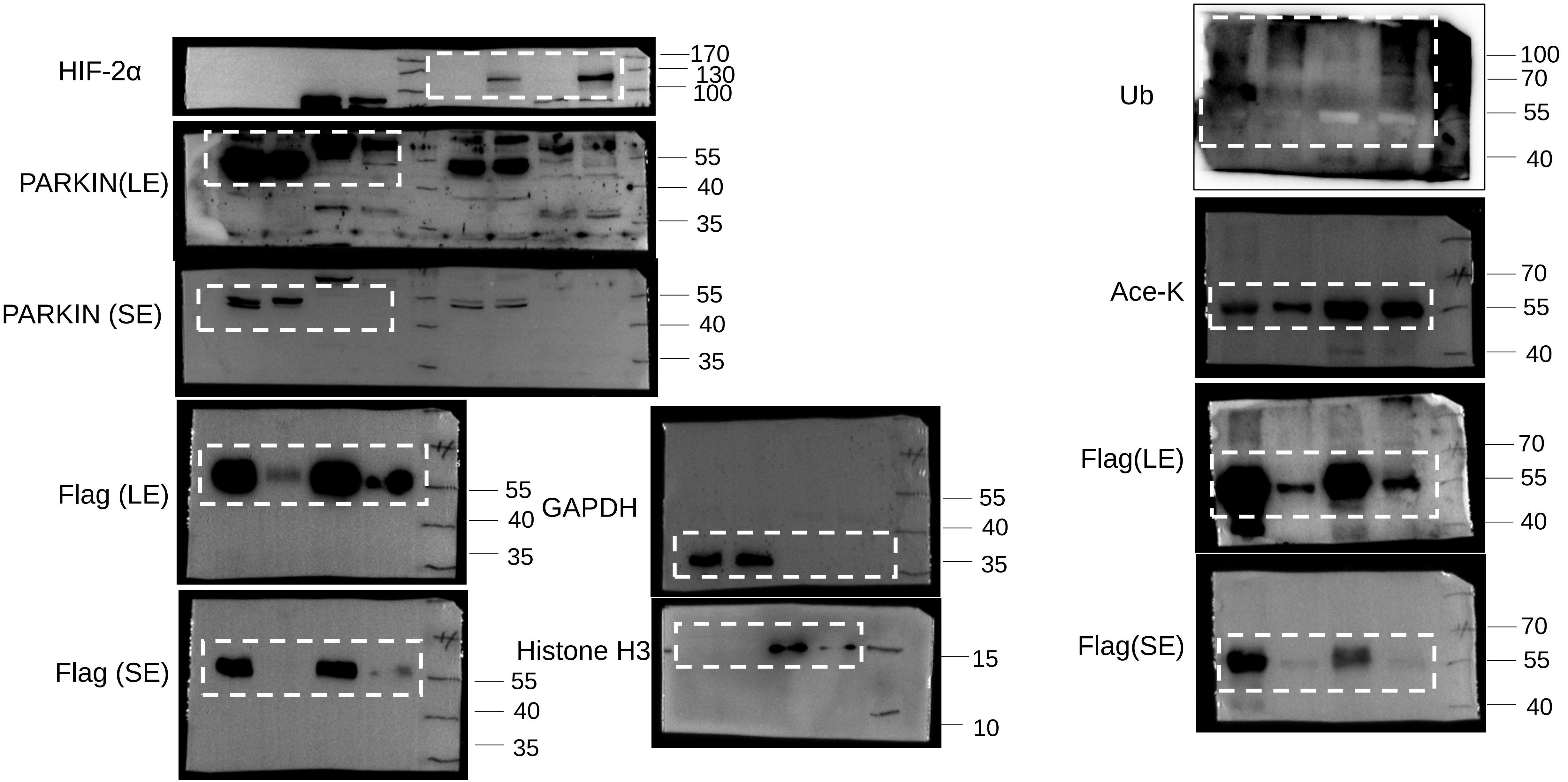

HIF-2α
170
130
100
PARKIN(LE)
55
40
35
PARKIN (SE)
55
40
35
Flag (LE)
55
40
35
Flag (SE)
55
40
35
GAPDH
55
40
35
Histone H3
15
10
Ub
100
70
55
40
Ace-K
70
55
40
Flag(LE)
70
55
40
Flag(SE)
70
55
40

Figure S4B

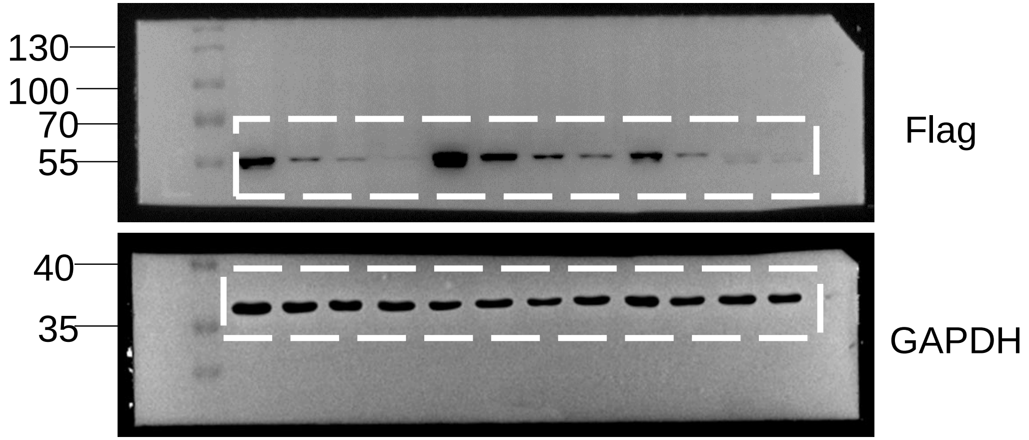

Figure S4D

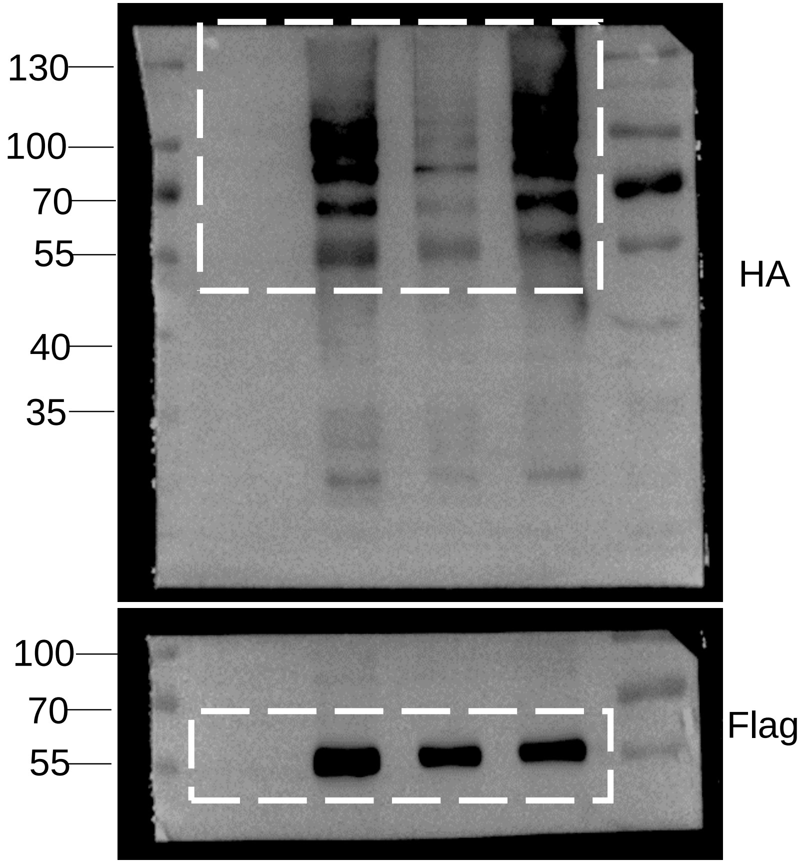

130
100
70
55
40
35
HA
100
70
55
Flag

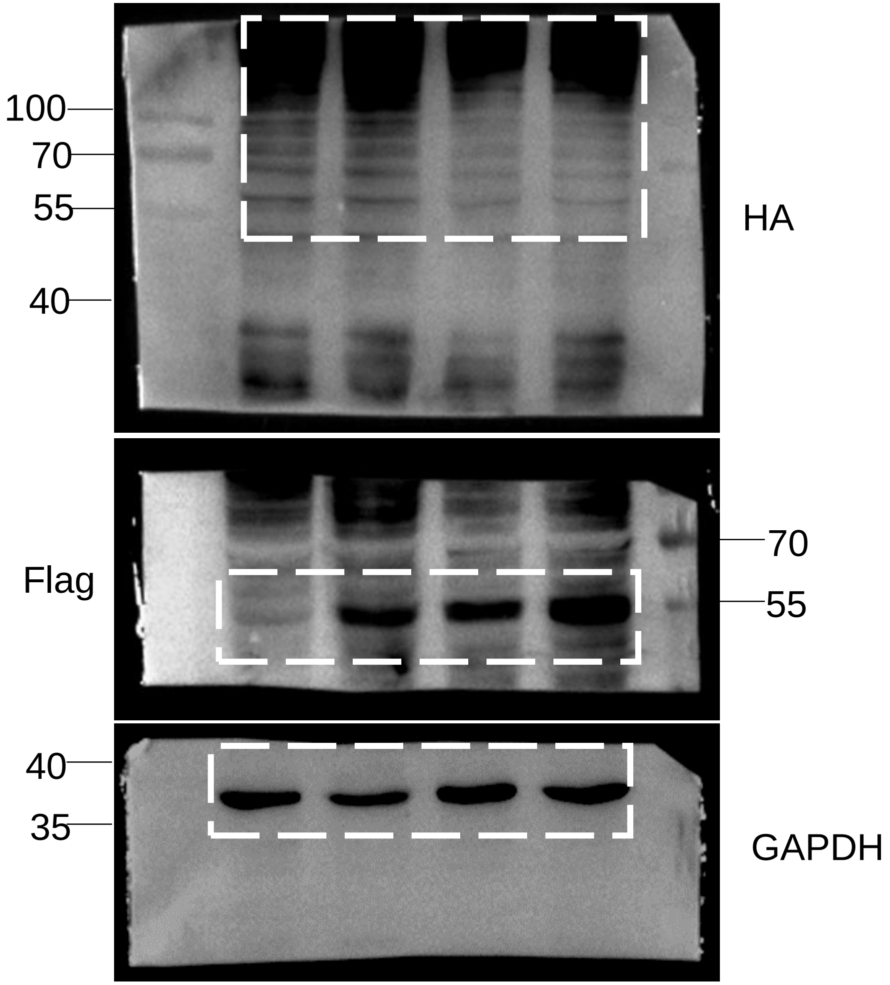

100
70
55
40
HA
Flag
70
55
40
35
GAPDH

Figure S7B

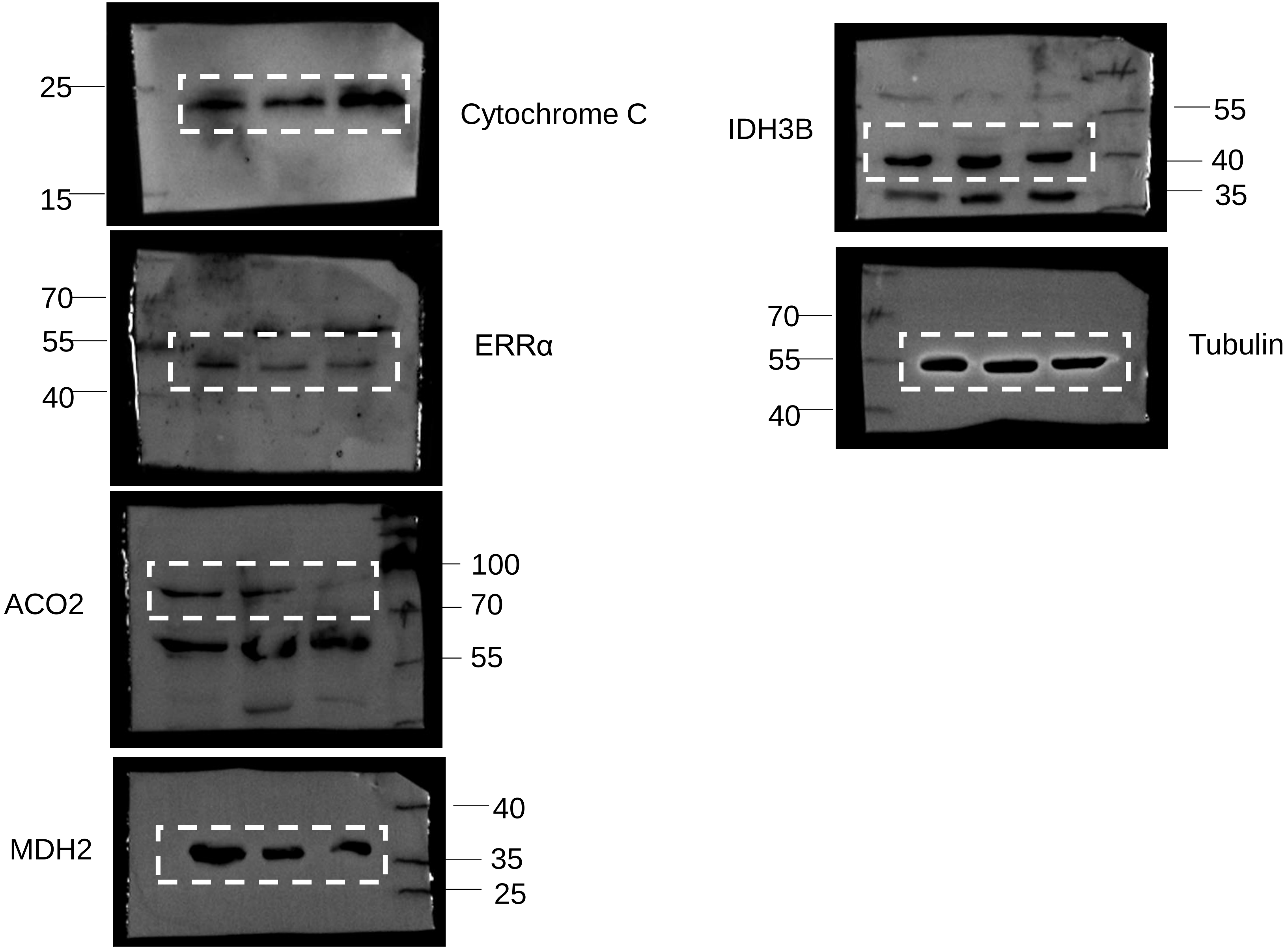
25
15
Cytochrome C
70
55
40
ERRα
ACO2
100
70
55
MDH2
40
35
25
IDH3B
55
40
35
70
55
40
Tubulin

Figure S7H

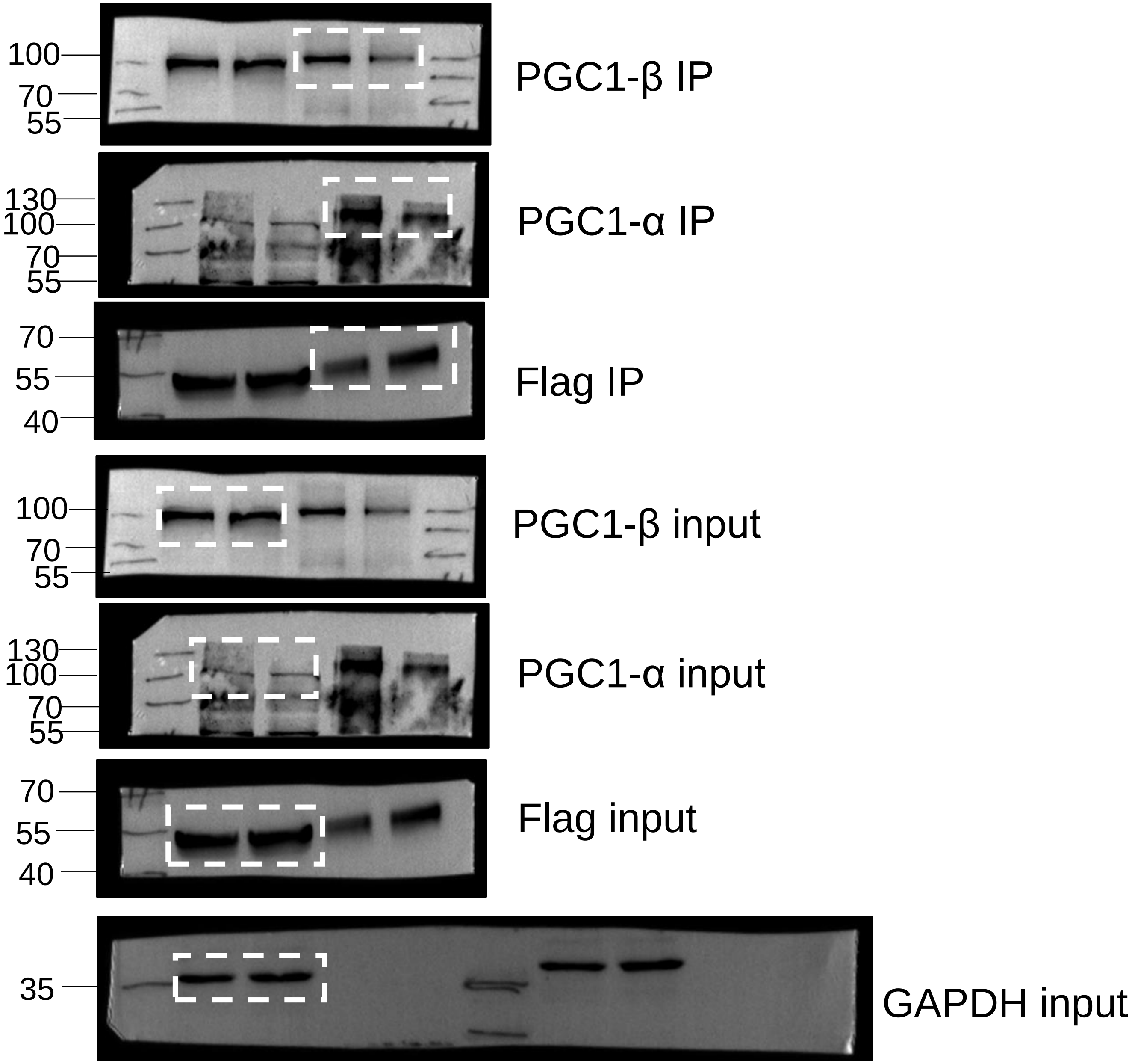
100
70
55
PGC1-β IP
130
100
70
55
PGC1-α IP
70
55
40
Flag IP
100
70
55
PGC1-β input
130
100
70
55
PGC1-α input
70
55
40
Flag input
35
GAPDH input
